# Sputum Liquid Biopsy for Lung Cancer Screening, Diagnosis, Subtyping, Surveillance, Response Prediction, and Prognostication: A Scoping Review

**DOI:** 10.3390/medsci14020231

**Published:** 2026-04-30

**Authors:** Abdul Rehman, Muhammad Awais, Hafiza Noor Ul Ain Baloch, Muhammad Omer Leghari, Arfa Ahmad, Hafiz Javed

**Affiliations:** 1 Department of Medicine, TidalHealth Peninsula Regional, Salisbury, MD 21801, USA; noor2000_2004@hotmail.com (H.N.U.A.B.); muhammad-omer.leghari@tidalhealth.org (M.O.L.); arfa.ahmad@tidalhealth.org (A.A.); hafiz.javed@tidalhealth.org (H.J.); 2 Department of Radiology, Aga Khan University Hospital, Karachi 74800, Sindh, Pakistan; awais_aku@yahoo.com

**Keywords:** lung neoplasms, liquid biopsy, tumor biomarkers, neoplasm genetic markers, sputum, circulating tumor DNA, cell-free nucleic acids, circulating tumor cells

## Abstract

**Background/Objectives**: Liquid biopsy (LB) is transforming cancer care by enabling minimally invasive tumor profiling. While current research and clinical pathways mostly focus on blood LB, sputum represents a non-invasive, readily available respiratory specimen that may offer unique advantages for lung cancer (LC) care. Despite its potential, the maturity, breadth, and clinical applicability of sputum-based LB remain elusive. **Methods**: We conducted a scoping review to systematically map the existing literature on sputum LB in LC. Electronic databases were searched for studies evaluating sputum-derived biomarkers—cytologic, genomic, epigenetic, transcriptomic, proteomic, metabolomic, metagenomic, and extracellular vesicle–derived products—across the LC care continuum. Study designs, technologies, clinical contexts, and reported outcomes were extracted and synthesized qualitatively. **Results**: The literature demonstrated substantial heterogeneity in sputum collection, processing, and analytical platforms. Early work focused on cytometry and genetic alterations, while recent studies increasingly explore DNA methylomics, microRNAs, extracellular vesicle-derived products, and multi-omics approaches. The evidence suggests potential utility of sputum biomarkers for early detection and risk stratification, particularly in high-risk populations, with emerging data supporting roles in molecular subtyping, response monitoring, prognostication, and surveillance. However, few studies report prospective validation, direct comparison with blood-based LB, or impact on actual patient outcomes. **Conclusions**: Sputum LB is a promising yet underdeveloped modality in LC care. This scoping review highlights technological innovations alongside significant methodological heterogeneity and translational gaps. Future research should focus on standardization, prospective validation, impact on patient outcomes, and integration with blood- and other body fluid–based LB, as well as imaging biomarkers. This will enable incorporation of sputum-based LB into actual clinical pathways of LC care.

## 1. Introduction

Lung cancer (LC) remains the third most commonly diagnosed cancer in adults and is the most common cause of cancer-related deaths worldwide [1,2,3]. The American Cancer Society estimates that, during the year 2026, 229,410 new cases of LC will be diagnosed and 124,990 deaths will be caused by LC—which is more than the cancer-related deaths caused by colorectal cancer and pancreatic cancer combined [2]. Similar trends are reported for European countries: it is estimated that LC will cause 215,300 deaths in European Union countries and 31,600 deaths in the United Kingdom during the year 2026 [3]. Trends for Asian countries are likely worse, based on extrapolation from data from the Global Cancer Observatory (GLOBOCAN) 2022: age-standardized mortality rates for Turkey, China, and East Asian countries were 35.06 (per 100,000), 32 (per 100,000), and 25.13 (per 100,000), respectively [1]. LC survival rates remain poor, despite substantial advances in imaging, molecular diagnostics, and systemic therapies [4].

Early detection, accurate molecular subtyping, longitudinal surveillance, and timely prediction of treatment response represent central priorities in contemporary LC research [5]. Low-dose computed tomography (LDCT) screening demonstrated a mortality benefit in high-risk populations in the National Lung Screening Trial (NLST), the NELSON trial, and the ITALUNG trial [6,7,8]. However, LDCT screening is fraught with false-positive findings, overdiagnosis, cumulative radiation exposure, and restricted accessibility in many healthcare systems [9,10,11]. Although tissue biopsy remains the reference standard for diagnosis and molecular profiling of LC, it is an invasive procedure with significant risk for complications, can be technically challenging at times, and is not feasible for repeated sampling during surveillance or response monitoring [10,12]. These limitations drove growing interest in minimally invasive diagnostic approaches, particularly liquid biopsy [12].

Liquid biopsy (LB) is a non-invasive method of detecting tumor cells, tumor-associated products, or other cargo embedded within extracellular vesicles (EVs) inside bodily fluids [13]. Blood-based LB has been extensively studied in the literature and is already in clinical use for detection of actionable genomic alterations (AGAs), as well as for identifying resistance in patients with non–small cell LC (NSCLC) [14,15,16]. Commercially available blood-based LB tests that can be used for comprehensive biomarker profiling in patients with NSCLC include FoundationOne^®^ Liquid CDx (Foundation Medicine, Inc.; Boston, MA, USA), Guardant360^®^ CDx (Guardant Health, Inc.; Palo Alto, CA, USA), Agilent Resolution ctDx^®^ FIRST (Agilent, Inc.; Santa Clara, CA, USA), and Neo PanTracer^®^ LBx (NeoGenomics Laboratories, Inc.; Fort Myers, FL, USA). Additionally, a blood-based LB test, Shield^®^ (Guardant Health, Inc.; Palo Alto, CA, USA), received FDA (Food and Drug Administration) approval for colorectal cancer screening in 2024 [17]. Blood-based LB is invaluable in the care of patients with advanced or metastatic LC, but its role in detecting and evaluating early-stage cancers is often limited, given that shedding of tumor cells and tumor-associated products in the systemic circulation may be minimal [18]. Perhaps this explains why the NHS Galleri trial—the largest clinical trial of multicancer early detection tests—failed to meet its primary clinical endpoint of reducing the incidence of stage III–IV cancers [19], even though the overall results were generally encouraging [20]. Having said this, the Nodify Lung^®^ (Biodesix, Inc.; Louisville, CO, USA) nodule risk assessment strategy, which combines the results of two blood tests (Nodify CDT^®^ and Nodify XL2^®^; Biodesix, Inc.; Louisville, CO, USA) alongside clinical information, can potentially reduce unnecessary procedures and improve early LC diagnosis [21]. These blood-based proteomics LB tests were validated in the PANOPTIC, FHCC, CLARIFY, and ORACLE cohorts [22].

**Figure 1:**
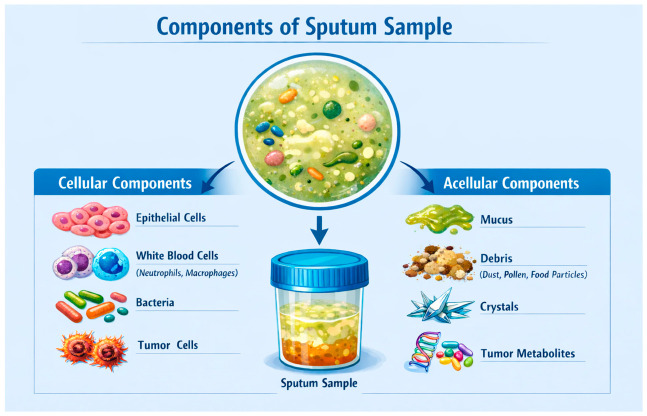
Cellular and acellular components of a sputum sample. A schematic diagram demonstrating the heavy biological noise contained in sputum samples from inflammatory cells and non-cancerous elements.

Sputum-based LB represents a biologically attractive yet comparatively underexplored modality for the care of patients with LC [23]. As a respiratory tract specimen derived directly from the airways, sputum can provide an indirect but up-close snapshot of the tumor microenvironment, potentially better than peripheral blood can [24]. Tumor cells and tumor-derived products from central and peripheral LCs are exfoliated into airway secretions, potentially enabling earlier detection and higher analytical sensitivity [25]. Moreover, sputum sampling affords a cheap, painless, and non-invasive means for LB, which can be monitored longitudinally over time [24,25]. However, sputum poses its own unique challenges as a medium for LB, including potential contamination with oral microbes, the barrier posed by viscous mucus film, variable quality based on collection and processing methods, and heavy biological noise from non-tumor cells [26] (see Figure 1).

Over the preceding two decades, multiple preclinical feasibility and clinical studies have evaluated sputum-based assays for diverse applications across the LC care continuum, including screening, diagnosis, molecular subtyping, prognostication, prediction of therapeutic response, and surveillance for recurrence [27]. Historically, sputum cytology (in conjunction with plain radiography) played a role in LC diagnosis, particularly for centrally located tumors, but its limited sensitivity curtailed widespread adoption [28]. Advances in molecular technologies—including next-generation sequencing (NGS), digital polymerase chain reaction (PCR), DNA methylation profiling, transcriptomics, proteomics, and EV analysis—renewed interest in sputum as a rich source of biomarkers [29].

Despite a growing body of literature, the evidence base for sputum LB has remained fragmented. Studies vary widely in terms of patient populations, sputum collection techniques, analytical platforms, biomarker targets, clinical endpoints, and study design. Furthermore, sputum-based investigations are distributed across multiple disciplines, including pulmonology, oncology, pathology, molecular diagnostics, and bioengineering, complicating a coherent synthesis. Scoping reviews are particularly well suited to emerging and heterogeneous research domains, where the objective is to map available evidence, clarify concepts, and identify gaps, rather than to generate pooled effect estimates [30]. Accordingly, we performed this scoping review to comprehensively map the published literature on sputum LB for LC. By systematically mapping the evidence available on sputum LB across the LC care continuum—screening, diagnosis, subtyping, response prediction, response monitoring, surveillance, and prognostication—we aimed to assess the translational readiness of the available evidence, identify methodological strengths and limitations, reveal gaps in the current literature, and delineate priorities for future research.

## 2. Materials and Methods

### 2.1. Scoping Review Methodology and Reporting Framework

This study was conducted as a scoping review of the published literature following guidance from the Joanna Briggs Institute (JBI) for scoping reviews [30] and was reported in accordance with the Preferred Reporting Items for Systematic Reviews and Meta-Analyses extension for Scoping Reviews (PRISMA-ScR) [31]. The review protocol was prospectively registered on the Open Science Framework prior to study initiation and is freely available online [32].

### 2.2. Eligibility Criteria

Eligibility criteria were defined a priori and were aligned with the Population–Concept–Context framework recommended for scoping reviews. All original research studies and conference abstracts that evaluated sputum-based testing for diagnosed or suspected LC were eligible for inclusion. Randomized trials, non-randomized studies, diagnostic accuracy studies, cohort and case–control studies, cross-sectional studies, and feasibility studies were included. Systematic reviews, meta-analyses, and scoping reviews with reproducible methodologies were also included for contextual support. Editorials, commentaries, narrative reviews, and opinion pieces were excluded, although their reference lists were manually screened to identify potentially eligible studies. Our primary focus was on clinical studies performed on sputum samples for screening, diagnosis, subtyping, prognostication, response prediction, surveillance of recurrence, and monitoring of response to therapy for patients with suspected or diagnosed LC. Although we included studies evaluating spontaneously expectorated or induced sputum, we did not include studies pertaining to other respiratory tract samples (such as tracheal aspirate, bronchial washing, bronchial brushings, or bronchoalveolar lavage) unless they included sputum specimens as well. Although our main focus was on studies of human sputum, we also included studies utilizing bronchial cell cultures or LC cell lines if they included testing or experiments utilizing sputum samples. With respect to LB, we planned to include studies testing for any type of products in the sputum, including circulating tumor cells (CTCs), EV/exosomes, circulating tumor DNA (ctDNA), cell-free DNA (cfDNA), microRNAs (miRNAs), tumor metabolites, autoantibodies, peptides, and so forth.

### 2.3. Information Sources and Search Strategy

A comprehensive literature search was conducted using PubMed/MEDLINE, Scopus, Embase, and Google Scholar, supplemented by searches of grey literature sources including OpenGrey and OAIster. Clinical trial registries searched included CENTRAL, ClinicalTrials.gov, WHO ICTRP, and the EU Clinical Trials Register. Search strategies combined terms related to sputum or respiratory specimens, LC, and LB- or tumor-derived biomarkers, using database-specific controlled vocabulary and free-text terms. The complete search strategies for each major database were predefined and implemented as registered in the online protocol [32].

### 2.4. Study Selection and Data Charting

Study selection was performed independently by two reviewers following removal of duplicate records. Titles and abstracts were screened, followed by full-text review of potentially eligible studies. Disagreements were resolved by consensus amongst the authors.

Data were extracted using a standardized charting form developed a priori and pilot-tested on a subset of studies. Extracted variables included study identification, study design, population characteristics, definition of cases and controls (if applicable), sputum collection methodology, biomarkers evaluated, analytical platforms, reference standard, histologic subtypes and stages of LC, clinical applications, performance measures (such as area under the receiver operating characteristic curve [AUROC], sensitivity, and specificity), and authors’ conclusions.

### 2.5. Risk of Bias Assessment and Critical Appraisal

Methodological quality was assessed to contextualize findings. Diagnostic accuracy studies were evaluated using QUADAS-2 (Quality Assessment of Diagnostic Accuracy Studies), and prognostic biomarker studies were evaluated using QUIPS (Quality in Prognostic Studies) [33]. Other observational studies were assessed using BIOCROSS (Biomarker-based Cross-Sectional Studies), ROBINS-I, the Newcastle–Ottawa Scale, or other JBI critical appraisal tools, as deemed appropriate [34]. We planned to appraise randomized trials using the RoB 2 tool, although no such studies were included in the final synthesis.

### 2.6. Data Synthesis

The findings were synthesized descriptively and presented in narrative and tabular formats, stratified by clinical application, biomarker class, and study design. Quantitative meta-analysis was not planned a priori due to anticipated heterogeneity. During the preparation of this manuscript, the authors used ChatGPT version 5.4 (OpenAI, San Francisco, CA, USA) and Google Gemini’s Nano Banana version 2.0 (Google LLC, Mountain View, CA, USA) to assist in generating figures. The authors have reviewed and edited the output and take full responsibility for the content of this publication.

## 3. Results

### 3.1. Study Selection and Inclusion

**Figure 2:**
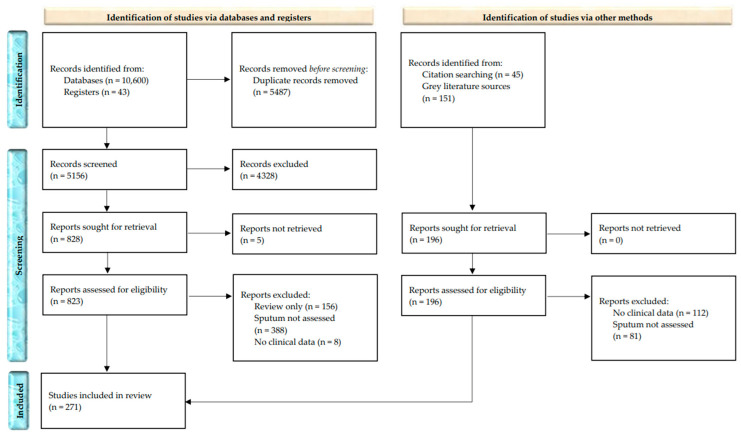
PRISMA-ScR flow diagram. A flow diagram depicting the inclusion and exclusion of various data sources within this scoping review in line with PRISMA-ScR guidance [31].

A total of 270 studies were included in this scoping review of sputum LB for screening, diagnosis, subtyping, response prediction, response monitoring, surveillance, and prognostication in LC (see Figure 2).

### 3.2. Characteristics of Sources

**Figure 3:**
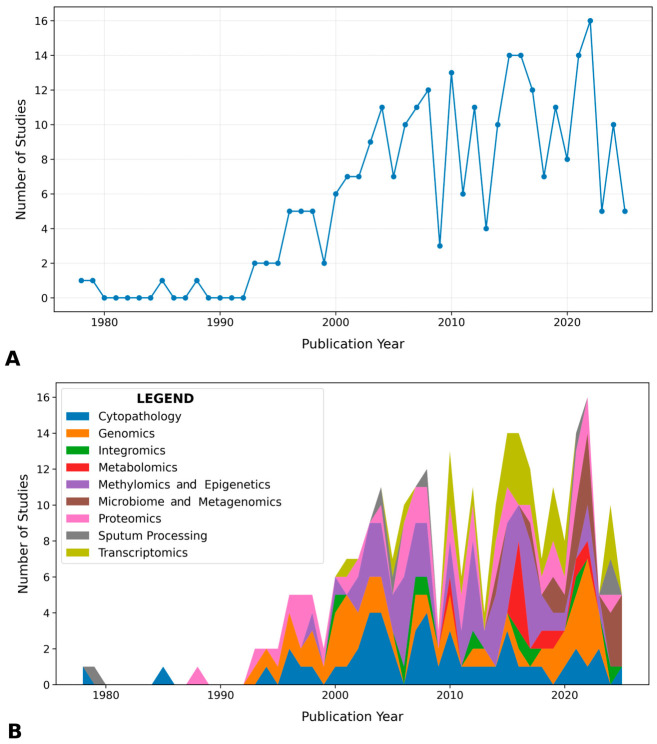
Number of relevant studies published over time. (**A**) A graph depicting the overall number of studies published over time. (**B**) A breakdown of studies by biomarker category over time.

The included literature spanned 1978 to 2026, demonstrating sustained interest over nearly five decades and a marked increase in publications over time (see Figure 3). Only 4 studies were published before 1990, compared with 23 in the 1990s, 83 in the 2000s, 102 in the 2010s, and 58 from 2020 onward.

**Figure 4:**
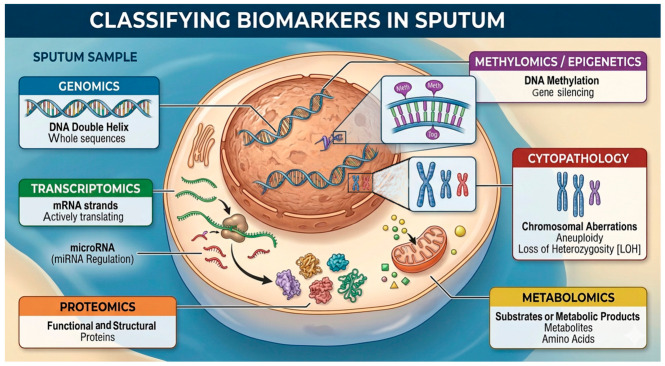
Classification of biomarkers within sputum. A figure depicting the various categories of biomarkers assessed within sputum samples in the published literature.

**Figure 5:**
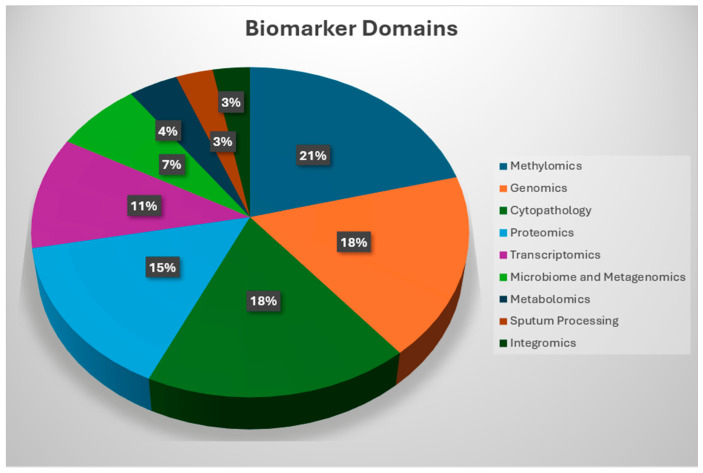
Proportion of included studies by biomarker categories. A pie chart depicting the proportion of studies included in this scoping review by their biomarker category.

The evidence base was distributed across nine predefined domains (Figure 4). Methylomics represented the largest category (57/270, 21%), followed by genomics (49/270, 18%), cytopathology (49/270, 18%), proteomics (41/270, 15%), and transcriptomics (29/270, 11%). Smaller but increasingly contemporary bodies of literature were identified in metagenomics (19/270, 7%), metabolomics (10/270, 4%), integromics (9/270, 3%), and sputum processing (7/270, 3%), as shown in Figure 5.

Among included studies, the literature was geographically diverse: China and the United States were the most frequently represented countries, with additional contributions from India, Japan, Italy, France, South Korea, Canada, Spain, Brazil, Iran, and other countries. Most studies were observational in design and were primarily framed as diagnostic biomarker investigations. Cohort sizes were generally modest, with a median reported total sample size of 91 participants.

### 3.3. Reporting Quality and Risk of Bias

Reporting of diagnostic performance metrics was inconsistent across studies. Explicit diagnostic performance metrics (such as sensitivity, specificity, and AUROC) were reported in 173 studies (64.1%). Positive and negative predictive values were rarely reported, even in prospective cohort studies with an intended screening use-case. The limited and uneven reporting of performance metrics, together with substantial heterogeneity in study populations, assay methods, comparators, and thresholds, precluded a robust cross-study quantitative synthesis. In view of this, we adopted a descriptive evidence mapping approach.

**Figure 6:**
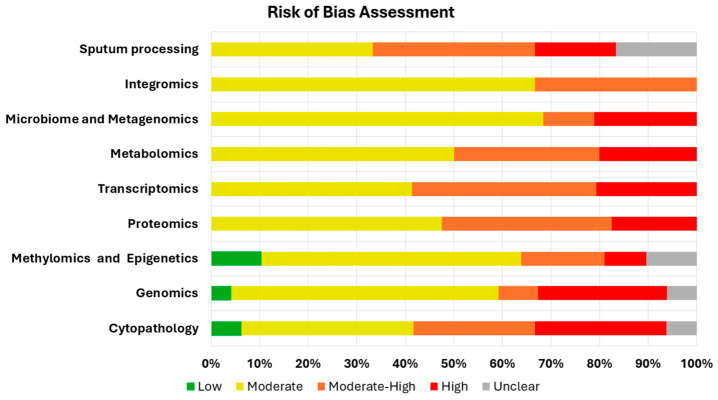
Risk of bias assessments by biomarker categories. A stratified bar chart depicting the risk of bias among the studies included within each biomarker category.

Based on risk-of-bias assessments, 11 (4%), 132 (49%), 61 (23%), and 50 (19%) studies were deemed to be at low, moderate, moderate-high, and high risk of bias, respectively (see Figure 6). Overall, the evidence base was characterized by substantial methodological limitations. Recurrent concerns included selective or clinically enriched patient populations (introducing spectrum bias), non-representative healthy controls, insufficient detail on index test conduct and threshold derivation, incomplete reporting of flow and timing, incomplete reporting of blinding, lack of multivariable adjustment for confounders, and limited external validation. These issues were especially common in case–control–style biomarker discovery studies.

### 3.4. Evidence Mapping and Synthesis by Biomarker Domain

**Figure 7:**
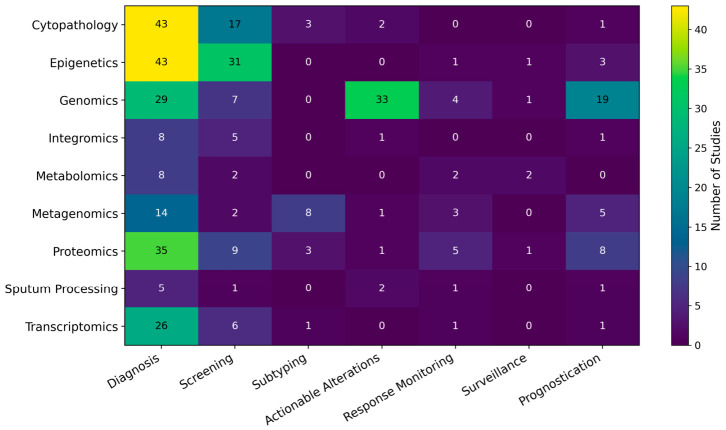
Studies with implications for specific clinical use cases, stratified by biomarker categories. A heatmap depicting the volume of published studies, stratified by biomarker category, with implications for each clinical use case. Each study could contribute to multiple clinical use cases.

The predominant clinical application was diagnosis, which was identified in 211/270 studies (78%). Many studies were also classified as relevant to screening (80/270, 30%), detection of AGAs (40/270, 15%), and prognostication (39/270, 14%). Fewer studies addressed response monitoring (17/270, 6%), subtyping (15/270, 5%), and surveillance (5/270, 2%). Because individual studies could contribute to more than one use case, these categories were not mutually exclusive (see Figure 7). Nonetheless, the overall pattern was clear: the sputum LB literature has focused predominantly on early detection and diagnostic classification, whereas applications in longitudinal monitoring, histologic subtyping, and surveillance remain comparatively underdeveloped.

#### 3.4.1. Cytopathology

Cytopathology comprised 49 published studies (see Appendix A.1) and represented one of the oldest streams of literature, with a median publication year of 2007. These studies compromised two major groups: (a) morphologic assessment of exfoliated cells using cytology, flow cytometry, and/or automated DNA cytometry; and (b) FISH (fluorescent in situ hybridization)-based techniques for detection of chromosomal aberrations and microsatellite alterations, such as LOH (loss of heterozygosity) and MSI (microsatellite instability). An overall summary of the results from these studies is presented in Table 1.

Traditional cytology (Papanicolaou smear) formed an important historical foundation for the field and established sputum as a clinically relevant respiratory specimen. However, the literature also reflected longstanding limitations of conventional sputum cytology, including variable sensitivity, dependence on specimen adequacy, and susceptibility to operator- and interpreter-related variability [35].

Sputum-based automated DNA cytometry in conjunction with flow cytometry was validated in multiple case–control and cohort studies [36,37,38,39]. Current clinical trials are exploring the role of sputum-based automated DNA cytometry for screening of LC. In particular, CyPath^®^ Lung (bioAffinity Technologies, Inc.; San Antonio, TX, USA) is being evaluated in a multicenter trial that started recruitment in early 2026 and is anticipated to be completed in 2029 [40]. CyPath^®^ Lung (bioAffinity Technologies, Inc.; San Antonio, TX, USA) uses meso-tetra (4-carboxyphenyl)-porphyrin-labeled sputum in conjunction with flow cytometry and artificial intelligence to detect malignant cells in an automated manner [36]. Another study by Qiu et al. explored magnetic-activated cell sorting (MACS) for improving sputum-based cytologic diagnosis of LC [41].

FISH-based approaches for cytopathologic diagnosis of LC in sputum specimens focused on chromosomal aberrations and microsatellite alterations. Sputum microsatellite analyses appeared technically feasible and biologically informative, with signals detectable in some patients with established LC, and occasionally in heavy smokers, but the studies were limited by small numbers, proof-of-concept designs, lack of standardized marker panels, and generally moderate to high risk of bias [42,43,44,45]. Across studies evaluating chromosomal aneusomies and related genomic aberrations in sputum, FISH-based assays generally demonstrated higher sensitivity than conventional sputum cytology, although performance varied substantially by clinical setting, assay platform, and study design [28,46,47]. Beyond targeted FISH assays, Arvanitis et al. [48] showed, using 48 microsatellite markers, that sputum fractional allele loss was approximately 10-fold higher in LC than in controls. Overall, chromosomal aberration assays in sputum consistently outperformed sputum cytology, where compared, and appeared most promising in high-risk or diagnostically enriched populations, but interpretation is tempered by frequent case–control designs, small sample sizes, inadequate-specimen exclusions, and generally moderate to high risk of bias.

Telomerase activity was explored across three prospective diagnostic studies from India [49,50,51]. In all studies, a TRAP (Telomerase Repeat Amplification Protocol) assay was used, and generally favorable but variable performance was reported. However, all of these studies were conducted in clinically suspected, predominantly advanced-stage, populations, rather than in screening cohorts. Moreover, TRAP assay sensitivity was inconsistent across studies, and false-positive results were observed in some non-malignant inflammatory pulmonary conditions.

**Table 1:** Overall summary of sputum-based cytopathology studies.

Cytopathology Approach	Representative Techniques	Representative Studies	Main Findings	Typical Setting	Key Limitations
Conventional sputum cytology	Papanicolaou smear; morphologic assessment of exfoliated cells	Historical cytology literature, including Payne et al. (1997) [35]	Established sputum as a clinically relevant respiratory specimen; historical foundation for LC detection, but diagnostic yield variable	Historical screening and diagnostic studies	Variable sensitivity; dependence on specimen adequacy; operator- and interpreter-related variability
Automated DNA cytometry/flow cytometry	Quantitative microscopy; automated DNA cytometry; flow cytometry; porphyrin-labeling of sputum; AI-assisted classification	Patriquin et al. (2015) [36] Rebel et al. (2021) [37] Bederka et al. (2022) [38] Bauta et al. (2023) [39]	Promising discrimination between malignant and non-malignant sputum samples; supported contemporary assay development, including CyPath^®^ Lung (bioAffinity Technologies, Inc.; San Antonio, TX, USA)	Case–control and cohort studies, with evolving interest in screening applications	Heterogeneous designs and platforms; limited prospective screening validation; incomplete standardization across studies
Cell enrichment for cytologic diagnosis	Magnetic-activated cell sorting (MACS)	Qiu et al. (2008) [41]	Magnetic enrichment of bronchial epithelial cells appeared to improve recovery of diagnostically relevant cells from sputum	Diagnostic enrichment setting	Limited external validation and sparse follow-up literature
FISH/chromosomal aberration assays	FISH for chromosomal aberrations; aneusomy panels; chromosomal copy number abnormalities	Jia et al. (2000) [44] Baron et al. (2017) [47] Shlomi et al. (2018) [46]	FISH-based chromosomal aberration assays generally demonstrated higher sensitivity than conventional sputum cytology in head-to-head comparisons; appeared especially promising in high-risk populations	High-risk or diagnostically enriched cohorts	Performance varied by assay platform, clinical setting, and study design; small or selected samples; moderate to high risk of bias
Microsatellite alteration assays	Loss of heterozygosity (LOH); microsatellite instability (MSI); fractional allele loss panels	Mao et al. (1994) [42] Miozzo et al. (1996) [43] Castagnaro et al. (2007) [45] Arvanitis et al. (2003) [48]	Microsatellite-based analyses were technically feasible and biologically informative; abnormal signals detected in some patients with LC and occasionally in heavy smokers, with fractional allele loss markedly higher in cases than controls	Mostly proof-of-concept and small case–control studies	Small sample sizes; proof-of-concept designs; non-standardized marker panels; limited reproducibility data; generally moderate-high or high risk of bias
Telomerase-based assays	Telomerase repeat amplification protocol (TRAP) assay	Sen et al. (2001 and 2002) [49,50] Pasrija et al. (2007) [51]	Prospective diagnostic studies reported generally favorable but inconsistent performance for telomerase activity in sputum	Clinically suspected, predominantly advanced-stage, populations	Sensitivity inconsistent across studies; false-positive results in some non-malignant inflammatory conditions

Abbreviations: AI, artificial intelligence; FISH, fluorescence in situ hybridization; LC, lung cancer; LOH, loss of heterozygosity; MACS, magnetic-activated cell sorting; MSI, microsatellite instability; TRAP, telomerase repeat amplification protocol.

#### 3.4.2. Genomics

The genomics category included 49 published studies with a median publication year of 2008 (see Appendix A.2). This body of literature evaluated DNA-level alterations, including AGAs and related genomic signals, using PCR-based and sequencing-based approaches. Genomic studies were largely directed toward diagnostic discrimination, although some also explored screening and surveillance contexts. Across this category, the literature reflected a transition from single-analyte investigations toward broader panel-based strategies, but reporting remained heterogeneous with respect to patient spectrum, assay standardization, and clinical validation. An overall summary of the results from the genomics studies is given in Table 2.

Earlier work focused on the detection of *TP53* and *K-RAS* mutations in sputum samples from high-risk smokers and patients with suspected LC. These studies were predominantly PCR-based feasibility or diagnostic studies and showed that tumor-related mutations could be detected noninvasively, although performance varied considerably, and specificity was sometimes limited by field cancerization or mutation-positive non-cancer controls. For *K-RAS*, the earliest proof-of-concept study by Takeda et al. [52] detected a codon 12 mutation in one of five sputum samples from LC patients, establishing technical feasibility. In a case–control study, Ronai et al. [53] found *K-RAS* mutations in 48.6% (18/37) of NSCLC sputum samples versus 12.5% (5/40) of non-cancer lung disease controls, indicating modest sensitivity and imperfect specificity. Zhang et al. [54] later reported *K-RAS* codon 12 mutations in 10/22 (45.5%) sputum samples from LC patients, with significant tumor–sputum concordance and identical mutations in nine patients, supporting the biologic validity of sputum mutation analysis. However, Nakajima et al. [55] highlighted important limitations, showing *K-RAS* mutations in 15–19% of sputum samples from LC patients but also in 20% of non-oncologic controls and observing discordance between sputum and tumor in several paired cases. Chen et al. [56] detected tumor-specific *TP53* mutations in only 1 of 10 pre-diagnostic sputum smears from patients with known tumor mutations, indicating low sensitivity with early assays. By contrast, Wang et al. [57] reported *TP53* mutations in sputum from 30/54 LC patients (55.6%) and 2/114 benign pulmonary disease controls, corresponding to 55.6% sensitivity and 98.3% specificity, outperforming sputum cytology alone (35.2%). Similarly, Guo et al. [58] found *TP53* mutations in 27.6% of post-bronchoscopic sputum samples, which was significantly higher than sputum cytology (6.9%) in patients suspected of LC. Anderson et al. [59] also showed p53 protein overexpression in sputum collected before diagnosis in all six patients whose tumors harbored mutant *TP53*, but also in two of five patients without tumor *TP53* mutations, suggesting that p53-related abnormalities may reflect high-risk field changes rather than strictly tumor-specific events. Overall, sputum detection of *K-RAS* and *TP53* alterations provided early evidence that airway-exfoliated genetic abnormalities could be captured noninvasively and often improved upon cytology, but the literature was limited by small sample sizes, older low-sensitivity assays, heterogeneous designs, and generally moderate to high risk of bias.

Eleven studies focused on the detection of *EGFR* (epidermal growth factor receptor) mutations, which are considered AGAs in patients with NSCLC. Across studies evaluating *EGFR* mutations in sputum, assay performance varied markedly in terms of specimen quality, disease stage, and molecular platform, but newer approaches generally showed that sputum can serve as a feasible noninvasive substrate for tumor genotyping in selected patients. A prospective paired study of late-stage LC by Su et al. [60] reported high concordance between sputum and tissue *EGFR* testing using ARMS-PCR, with a sensitivity of 90.9%, a specificity of 96%, and an overall accuracy of 97.1%, albeit after cytologic enrichment and exclusion of tumor-cell–negative sputum samples. Studies focusing on sputum supernatant cfDNA reported more moderate overall sensitivity but consistently high specificity. For instance, Wang et al. [61] reported 46.2% sensitivity and 100% specificity for *EGFR* mutations overall, with sensitivity rising from 24.0% in stage I–IIIA disease to 65.0% in stage IIIB–IV disease and reaching 92.9% when malignant cells were seen on sputum cytology. Similarly, Isaka et al. [62] showed that sputum droplet digital PCR (ddPCR) had high sensitivity only in cytology-positive samples (80.0%) but extremely low sensitivity in cytology-negative samples (3.1%), despite 100% specificity in both groups. Diagnostic performance was superior in studies evaluating sputum supernatant cfDNA by NGS [25,63]. Overall, the evidence indicates that sputum can support *EGFR* mutation detection, particularly in advanced disease and in sputum samples with malignant cells or adequate tumor-derived DNA, but that sensitivity is inconsistent in unselected sputum. Additionally, most studies were limited by small size, selected populations, and moderate to high risk of bias.

Evidence for *BRAF* mutation and *EML4*-*ALK* fusion detection in sputum was sparse and was directed primarily toward molecular profiling of established NSCLC rather than screening or early diagnosis. For *BRAF*, the only sputum-related study identified was a preclinical method-development investigation by Emaus et al. [64], which used artificial sputum spiked with mutant DNA and showed that selective extraction with ion-tagged oligonucleotides and magnetic ionic liquids improved analytical sensitivity for low-abundance *BRAF* V600E detection to 0.1% of the mutant allele fraction. However, no patient-derived sputum samples were studied, so no clinical diagnostic inferences could be made. For *EML4*-*ALK*, clinical evidence was limited but more substantive: in a large, prospective, multicenter Japanese study, Soda et al. [65] demonstrated that multiplex RT-PCR could detect *EML4*-*ALK* fusion transcripts in sputum specimens from patients with confirmed NSCLC, with four positive sputum samples identified and concordance observed with paired tumor or effusion specimens, supporting the technical feasibility of sputum-based fusion testing. Wang et al. [66] further showed, in a retrospective cohort of 1274 patients with advanced NSCLC, that cytological specimens overall yielded *ALK* detection rates comparable to tissue analysis by RT-PCR, although the sputum subgroup comprised only two samples and therefore provided minimal sputum-specific evidence. Morikawa et al. [67,68] also reported on the feasibility and utility of the Lung Cancer Compact Panel^®^ (LCCP by DNA Chip Research, Inc.; Kanagawa, Japan) for detection of *EML4*-*ALK* gene fusions in cytologically positive sputum specimens. The LCCP LB test was approved by the Ministry of Health, Labor, and Welfare in Japan as a multi-companion diagnostic kit for LC in November 2022. Overall, the sputum literature for *BRAF* and *EML4*-*ALK* remains very limited, with clinical support confined mainly to proof-of-concept *ALK* fusion detection in confirmed NSCLC and no direct patient-based clinical validation for sputum *BRAF* mutation testing.

Across studies evaluating multi-gene sputum assays, panel-based approaches generally outperformed single-marker testing and conventional sputum cytology, although performance depended strongly on clinical setting, disease stage, and specimen adequacy. In early-stage disease, Jiang et al. [69] reported that a four-gene sputum mini-chip assay targeting *HYAL2*, *FHIT*, *p16*, and *SP-A* achieved 70% sensitivity and 92% specificity for stage I NSCLC; when combined with CT, the sensitivity increased to 91%, and the overall accuracy increased to 90%, with particularly favorable performance for central tumors. In a subsequent optimization study, the same group [70] developed a six-gene panel (*ENO1*, *FHIT*, *HYAL2*, *SKP2*, *p16*, and *14-3-3ζ*) that yielded sensitivities of 81.6% to 83.7% and a specificity of 93.9% for distinguishing stage I NSCLC from healthy smokers or COPD controls, markedly exceeding sputum cytology for early-stage disease (81.4% vs. 41.9% sensitivity). Within a CT-screening context, Carozzi et al. [71] applied a broader molecular profile including LOH/MSI at 12 loci, *K-RAS* and *TP53* mutations, and plasma DNA quantification, finding allelic imbalance in 84.2% of LC cases but also in 73.0% of participants with non-calcified nodules and 28.9% of negative controls, suggesting biologic signal but limited specificity when used alone. More recent multiplex genomic studies in advanced NSCLC extended this concept to larger mutation panels in sputum cfDNA: Qin et al. [24] showed that induced sputum supernatant outperformed sputum sediment for 168-gene profiling and achieved 69.2% concordance with tumor tissue, while Xie et al. [25] reported 77% concordance for sputum supernatant cfDNA using a 520-gene panel, increasing to 90% when combined with plasma. Likewise, in a sputum multi-driver panel study, Wang et al. [72] demonstrated that a 10-gene NGS assay detected hotspot alterations in sputum cfDNA with 81.3% overall sensitivity and 100% specificity relative to paired tumor samples, with sensitivity rising to 87.0% in stage IIIB–IV disease and 94.1% in advanced-stage patients with malignant sputum cytology. Overall, multi-gene sputum panels appear more informative than single-gene assays or cytology alone and may be especially useful when integrated with imaging or other LB sources; however, the evidence remains heterogeneous, spanning early case–control mini-chip studies to advanced-disease NGS profiling, with most studies carrying at least some concern for selection bias or limited generalizability.

**Table 2:** Overall summary of sputum-based genomics studies.

Domain	Representative Techniques	Representative Studies	Main Findings	Typical Setting	Key Limitations
*TP53* and *K-RAS* mutations	PCR-based mutation detection of *K-RAS* codon 12 and *TP53* alterations	Takeda et al. [52] Zhang et al. [54] Nakajima et al. [55] Chen et al. [56] Guo et al. [58] Anderson et al. [59]	Compared with cytology, molecular detection often improved sensitivity, but specificity was imperfect High-risk controls were mutation-positive (field cancerization)	Mainly high-risk smokers and patients with suspected or established LC; mostly feasibility, diagnostic, or case–control designs	Small sample sizes; older low-sensitivity platforms; heterogeneous pre-analytic methods; occasional tumor-sputum discordance; concern for field cancerization; overall risk of bias generally moderate to high
*EGFR* mutation	ARMS-PCR, ddPCR, sequencing-based techniques; sputum cell sediment or supernatant cfDNA	Su et al. [60] Wang et al. [73] Isaka et al. [62]	Sputum-based *EGFR* genotyping is feasible, especially in advanced NSCLC and in cytology-positive or tumor-enriched sputum Specificity was consistently high, whereas sensitivity varied markedly	Paired tumor-sputum samples from suspected or known NSCLC; often, advanced stage; cytology-positive sputum in some studies	Sensitivity inconsistent in unselected sputum; selected populations or exclusion of inadequate specimens; limited external clinical validation
*BRAF* mutation *EML4*-*ALK* fusion	*BRAF* V600E detection in virtual sputum; multiplex RT-PCR and LCCP for *EML4*-*ALK* fusion detection	Emaus et al. [64] Soda et al. [65] Wang et al. [66] Morikawa et al. [68]	Preclinical evidence of *BRAF* V600E mutation in artificial sputum Proof-of-concept clinical studies supported *EML4*-*ALK* fusion detection, but tiny sample size	Established NSCLC undergoing molecular profiling; mostly cytology-positive sputum	Extremely limited evidence base; findings mainly support feasibility
Multi-gene panels	Mini-chip and multiplex assays targeting combinations of genes or alterations	Jiang et al. [69] Jiang et al. [70] Carozzi et al. [71]	Panels consistently outperformed single-gene assays; favorable sensitivity and specificity in early-stage NSCLC, when combined with CT	Case–control and screening-enriched cohorts; several studies emphasized central tumors or CT-screening cohorts	Panel marker composition and thresholds varied considerably across studies; limiting comparability; selection bias; limited external validation
Broad NGS profiling of sputum cfDNA	Large targeted sequencing panels (e.g., 10-gene, 168-gene, or 520-gene panels)	Qin et al. [24] Xie et al. [25] Wang et al. [72]	Sputum cfDNA NGS demonstrated moderate to high concordance with paired tumor tissue Better performance in sputum supernatant than cell sediment Higher sensitivity in advanced disease	Advanced NSCLC; paired tissue comparator studies evaluating molecular profiling	Selected, advanced-disease cohorts; applicability to clinical settings uncertain; heterogeneity due to differences in panel size, specimen processing techniques, and adequacy criteria

Abbreviations: *ALK*, anaplastic lymphoma kinase; ARMS, amplification refractory mutation system; *BRAF*, v-raf murine sarcoma viral oncogene, homolog B; cfDNA, cell-free deoxyribonucleic acid; CT, computed tomography; ddPCR, droplet digital polymerase chain reaction; *EGFR*, epidermal growth factor receptor; *EML4*, echinoderm microtubule-associated protein-like 4; *K-RAS*, Kirsten rat sarcoma viral oncogene homolog; LC, lung cancer; LCCP, Lung Cancer Compact Panel^®^ (DNA Chip Research, Inc.; Kanagawa, Japan); LOH, loss of heterozygosity; NGS, next-generation sequencing; NSCLC, non–small cell lung cancer; p53, tumor protein 53; PCR, polymerase chain reaction; RT, reverse transcription.

#### 3.4.3. Methylomics and Epigenetics

Methylomics and epigenetics was the largest domain (57 studies) and one of the most prominent molecular areas in the sputum literature (see Appendix A.3), with a median publication year of 2012. This category contributed heavily to the screening and diagnosis evidence base and frequently evaluated multi-gene methylation panels rather than isolated loci. The prominence of this domain suggests that methylation-based assays have been especially attractive for sputum applications, likely owing to biological plausibility in early carcinogenesis and technical compatibility with low-input DNA extracted from sputum. An overall summary of the results for epigenetics and methylomics studies is provided in Table 3.

Sputum methylomics represents one of the most mature sputum biomarker domains, with studies spanning early biologic discovery, case-control diagnostic evaluation, prospective risk prediction, and post-resection surveillance. Early work established biologic plausibility by showing that p16 promoter methylation is detectable in sputum and increases across the histologic spectrum from basal cell hyperplasia and squamous metaplasia to carcinoma in situ and invasive squamous cell carcinoma, supporting methylation as an early event in lung carcinogenesis [74]. Subsequent studies demonstrated that methylation abnormalities are also frequent in smokers without LC, consistent with airway field cancerization; for example, *p16*, *DAPK*, *MGMT*, and *RASSF1A* methylation were detectable in bronchial epithelium and sputum from cancer-free smokers and persisted after smoking cessation, although this background prevalence limited specificity for cancer detection [75].

Multi-gene panels generally outperformed single-gene assays. In a cross-sectional risk study, increasing numbers of methylated genes in sputum were associated with higher LC risk, with greater than or equal to three methylated genes conferring an odds ratio of 6.2. The strongest prospective evidence came from a nested case-control analysis within the Colorado Sputum Screening Cohort [76], in which a six-gene panel (*p16*, *MGMT*, *DAPK*, *RASSF1A*, *PAX5b*, *GATA5*) predicted incident LC in a high-risk cohort up to 6 years before diagnosis; within 18 months of diagnosis, greater than or equal to three methylated genes yielded an adjusted odds ratio of 6.5, with 64% sensitivity and 64% specificity. In established NSCLC, sputum methylation also reflected tumor biology more closely than serum: in stage III disease, sputum methylation frequencies approximated tumor methylation frequencies, and a four-gene panel (*p16*, *DAPK*, *PAX5b*, *GATA5*) achieved a positive predictive value of 86% for tumor methylation status, whereas serum sensitivity was poor [77]. Broader methylation studies further suggested relevance to smoking-related risk phenotypes, including chronic mucous hypersecretion and recurrence after resection, although not all methylation markers were directly diagnostic for incident LC [78,79].

Novel approaches to methylomics and epigenetics have moved beyond locus-specific promoter methylation to assessment of global DNA methylation topology. Tajbaksh et al. [80] used three-dimensional DNA methylation imaging to differentiate normal respiratory epithelial cells from hypomethylated malignant cells in resected tumors and matching sputum based on in situ immunofluorescence (5-methylcytosine, 4′-6-diamidino-2-phenylindole co-localization). Likewise, Soukiasian et al. [81] utilized DNA methylation topology analysis in a sputum-based clinical assay that detected early-stage NSCLC with 95.8% sensitivity, albeit with limited specificity compared with high-risk benign lung disease. Additionally, Li et al. [82] used droplet digital MSP (methylation-specific PCR) to simultaneously quantify multiple miRNA and DNA methylation sites and develop an integromic signature for detection of LC.

A recent systematic review and meta-analysis by Wen et al. [27] synthesized 15 sputum cfDNA methylomics studies and found substantial heterogeneity in diagnostic performance, with reported sensitivities ranging from 10% to 93% and specificities from 8% to 100%; pooled across all genes, the summary sensitivity was 54.3%, and the specificity was 79.7%, with an HSROC AUC of 0.71, indicating moderate overall discriminatory ability. The review identified *RASSF1A*, *APC*, and *CYGB* as the most frequently studied genes, but their pooled sensitivities were modest, at 39%, 44%, and 47%, respectively, despite generally better specificities, especially for *RASSF1A*. Notably, two less frequently studied genes, *SOX17* and *TAC1*, showed sensitivities above 85% with specificities above 70% in the most recent studies, suggesting that newer targets and newer platforms such as digital PCR may outperform older methylation panels. Wen et al. [27] also emphasized major sources of variability, including tumor location and stage, spontaneous versus induced sputum collection, whether cellular pellet or supernatant was analyzed, DNA extraction methods, and assay platform, with most studies still using case–control designs and QMSP (Quantitative Methylation Specific PCR)-based methods. Taken together, the available evidence indicates that sputum methylomics is biologically robust and promising for risk stratification and adjunctive early detection, but current performance remains too heterogeneous for stand-alone clinical implementation, and methodological standardization plus validation of high-performing genes will be essential for routine clinical use.

**Table 3:** Overall summary of sputum-based methylomics and epigenetics studies.

Domain	Representative Techniques	Representative Studies	Main Findings	Typical Setting	Key Limitations
Proof-of-concept	Conventional MSP	Belinsky et al. [74]	Biologic plausibility of sputum methylation as an early event in lung carcinogenesis	Early biologic discovery and translational studies in smokers and LC patients	Primarily mechanistic and observational; limited immediate clinical validation for stand-alone diagnosis
Field cancerization in smokers	Nested MSP, MSRE-PCR	Belinsky et al. [75] Rosell et al. [83]	Methylation abnormalities are frequent, even in cancer-free smokers, and may persist after smoking cessation	High-risk smoking populations without known LC	Background prevalence reduces cancer specificity and complicates interpretation of isolated methylated loci
Multi-gene methylation panels	Multiplex MSP, MSRE-PCR	Belinsky et al. [76] Mohammed et al. [84]	Panel-based assays generally outperformed single-gene testing; increasing numbers of methylated genes correlated with higher lung cancer risk	High-risk smokers and known LC patients; case–control or cohort designs	Moderate sensitivity and specificity; predictive performance varied by time to diagnosis and cohort characteristics
Methylation markers linked to post-resection recurrence	Multiplex MSP, MSRE-PCR, CoBRA-MSP	Belinsky et al. [79] Tessema et al. [85]	Gene methylation in sputum post-resection was associated with odds of recurrence	High-risk screening or surveillance cohorts undergoing longitudinal follow-up	Few methylation markers were specific for recurrence
DNA methylation topology	3D quantitative DNA methylation imaging	Tajbakhsh et al. [80] Soukiasian et al. [81]	Sputum-based methylation topology can detect hypomethylated cancerous cells, potentially detecting early LC	High-risk smokers, LC patients, and COPD patients	Small sample size; limited prospective clinical validation; moderate to high-risk of bias
Systematic review of sputum cfDNA methylomics	QMSP and ddMSP	Wen et al. [27]	Sensitivity and specificity of sputum methylated tumor DNA for LC detection varied considerably; divergence relates to tumor site, sample acquisition, extraction methods, and methylation measurement techniques	Meta-analysis of 15 studies with substantial, but unquantified, heterogeneity	Substantial heterogeneity in study designs, sputum acquisition protocols, and methylation measurement techniques; however, no objective measure of heterogeneity reported

Abbreviations: CoBRA, combined bisulfite modification and restriction analysis; COPD, chronic obstructive pulmonary disease; ddMSP, droplet digital methylation-specific polymerase chain reaction; DNA, deoxyribonucleic acid; LC, lung cancer; MSP, methylation-specific polymerase chain reaction; MSRE, methylation-sensitive restriction enzyme; NSCLC, non–small cell lung cancer; PCR, polymerase chain reaction; QMSP, quantitative methylation-specific polymerase chain reaction.

#### 3.4.4. Proteomics

The proteomics category included 41 published studies (refer to Appendix A.5) with a median publication year of 2011. Proteomics studies of sputum were comparatively heterogeneous, ranging from early tumor-marker immunoassays and inflammatory protein panels to contemporary mass spectrometry discovery platforms and biomarker-enabled devices (see Table 4). Collectively, the available evidence suggests that sputum proteins may support LC detection, subtype discrimination, prognostication, surveillance, and possibly treatment-response assessment. The earlier literature mostly focused on single sputum-based biomarkers for diagnostic or classification purposes using immunohistochemistry or ELISA, while the more recent literature focused on high-dimensional quantitative proteomics for prognostication, response assessment, and surveillance.

Earlier work showed that conventional protein tumor markers in induced sputum could have diagnostic utility: CYFRA21-1 was approximately seven-fold higher in LC than COPD and achieved 86% sensitivity and 75% specificity, outperforming other measured markers such as CEA and NSE [86]. Other pilot studies suggested that combining inflammatory, angiogenic, autophagy, and adhesion-related proteins in induced sputum may improve case discrimination, as neutrophils, beclin-1, *VEGF*, *ICAM*, and *TNF*α differed significantly in LC versus COPD and healthy controls [87], although the derived combined score was exploratory and lacked full diagnostic calibration metrics. A separate immunocytochemical study of sputum cell blocks showed particularly strong diagnostic performance for proliferation-associated proteins, with *MCM2* yielding 80.3% sensitivity and 100% specificity and *MCM7* yielding 92.1% sensitivity and 100% specificity [88], suggesting that protein expression markers in exfoliated sputum cells may substantially augment conventional sputum cytology.

More recent proteomics literature employing high-dimensional quantitative proteomics (such as diaPASEF) demonstrated superior performance compared to traditional immunohistochemical methods. Arenas-De Larriva et al. [89] quantified 527 sputum proteins and identified inflammatory and immune-related differences between cancer and control samples, with CRP and *SERPINA1* among the most upregulated proteins; an internally cross-validated sPLS-DA model achieved an apparently excellent AUROC of 0.97 for cancer versus control and very high performance for SCLC, although the study was limited by small size and lack of external validation. In a different clinical role, Böttger et al. [90] identified 34 sputum-detectable secretome-derived proteins linked to cisplatin sensitivity, including *UGGT1*, *COL6A1*, and *MAP4*, supporting the feasibility of sputum proteomics for predictive biomarker development rather than diagnosis alone. Technology-driven approaches also showed promise: a portable sputum biosensor integrating CEA, NSE, and CA125 achieved a combined AUROC of 0.931, 87.0% sensitivity, and 86.5% specificity in a case–control cohort, while also demonstrating potential for longitudinal treatment monitoring [91]. Overall, sputum proteomics appears promising across several potential applications, but the evidence remains mostly early-phase, with many studies limited by case–control designs, modest sample sizes, incomplete external validation, or exploratory model development.

#### 3.4.5. Transcriptomics

Evidence in this domain comprised 29 published studies (Appendix A.4), with a later median year of publication, 2016, indicating a more contemporary wave of sputum biomarker research. These studies encompassed messenger RNA (mRNA) and non-coding RNA (ncRNA) approaches and were commonly framed as diagnostic or subtype-related investigations. Compared with DNA-based methods, transcriptomic assays may capture a broader and more dynamic biological signal, but they also appear to be more vulnerable to pre-analytic variability related to RNA integrity, cellular composition, and sputum handling (see Table 5).

Studies evaluating single mRNA, microRNA (miRNA), small nucleolar RNA (snoRNA), circular RNA (circRNA), and other RNA targets for LC diagnosis were small-scale and heterogeneous, although several showed meaningful incremental diagnostic value beyond conventional cytology. Among mRNA-based assays, survivin mRNA was commonly studied: Chen et al. [92] reported that adding sputum survivin RT-PCR to cytology increased diagnostic sensitivity from 47.1% to 80.2%, while Dong et al. [93] found survivin mRNA in 63.5% of sputum samples from LC patients and in none of the COPD controls, with combined survivin plus cytology improving sensitivity from 37.5% to 78.8% without loss of specificity. A large cross-sectional study by Chen et al. [94] further reported that template-ready PCR detection of *hTERT* mRNA in sputum achieved 84.2% sensitivity and approximately 96% specificity compared with non-malignant pulmonary disease controls, suggesting that assay innovation can substantially improve transcript-based detection. In the non-coding RNA space, Bagheri et al. [95] found that sputum miR-223 was markedly upregulated in NSCLC and yielded an AUROC of 0.90, with 82% sensitivity and 95% specificity, outperforming the other tested miRNAs and snoRNAs. Bai et al. [96] identified sputum circ_0006949 as a promising circRNA biomarker that was elevated in NSCLC compared with both healthy and lung infection controls and reportedly outperformed conventional serum tumor markers. By contrast, Lacroix et al. [97] showed only limited sputum positivity for preproGRP RT-PCR in SCLC (22%), indicating more modest clinical sensitivity despite strong analytical detection capability.

Across panel-based sputum transcriptomics studies, multi-marker RNA signatures generally outperformed single-analyte assays, although performance varied by platform, intended use case, and study design. Early miRNA panel studies showed promising diagnostic accuracy: Yu et al. [98] developed a four-miRNA panel (miR-486, miR-21, miR-200b, miR-375) that achieved 80.6% sensitivity and 91.7% specificity in the optimization cohort for stage I lung adenocarcinoma and retained moderate accuracy in an independent validation cohort (70.3% sensitivity, 80.0% specificity overall), with better performance for adenocarcinoma and peripheral tumors. For squamous cell carcinoma, Xing et al. [99] identified a three-miRNA panel (miR-205, miR-210, miR-708) with 73% sensitivity and 96% specificity in the optimization set and 72% sensitivity and 95% specificity in an independent validation cohort, with similar sensitivity across stages I–IV. Other sputum miRNA panels yielded comparable but somewhat lower performance, including a five-miRNA panel in Roa et al. [100] with 83.3% sensitivity and 100% specificity in a small double-blind validation cohort, a three-miRNA panel in Razzak et al. [101] with 67% sensitivity/90% specificity for early-stage NSCLC and 64%/100% for advanced disease, and a two-miRNA digital PCR panel in Li et al. [102] with 65.7% sensitivity and 85.0% specificity. Methodologically stronger integrative studies suggested that combining sputum RNA biomarkers with other modalities improved clinical utility. Shen et al. [103] showed that a sputum miR-31/miR-210 panel alone had modest sensitivity (~65%) but high specificity (~90%), and that combining with CT improved specificity from ~84% to ~92% while maintaining sensitivity above 92% across training and validation sets. Similarly, in smokers with CT-detected indeterminate solitary pulmonary nodules, Xing et al. [104] reported that a three-miRNA sputum panel (miR-21, miR-31, miR-210) achieved 82.9% sensitivity and 87.8% specificity in training, with stable performance in both internal and external testing cohorts (80.5–82.1% sensitivity, 86.1–88.4% specificity), indicating potential value for distinguishing malignant from benign nodules. Other panel approaches included a two-snoRNA panel (snoRD66, snoRD78) that yielded reproducible sensitivity and specificity of approximately 75% and 84% in both training and testing cohorts and improved the specificity of CT when combined with imaging [105,106]. A combined five-ncRNA sputum panel also reached 89.1% sensitivity and 89.1% specificity for stage I NSCLC [106], while a three-marker non-coding RNA panel (miR-145, miR-126, miR-7) reportedly achieved 90% sensitivity and 90% specificity [107], though these studies were more vulnerable to overfitting because validation was limited or internal only. Overall, panel-based sputum transcriptomics appeared more diagnostically informative than single-marker RNA assays and added value to LDCT or cytology. However, most included studies used case–control designs with hospital-based populations, and only a minority included robust external validation in clinically relevant nodule evaluation settings, which limits generalizability.

**Table 5:** Overall summary of sputum-based transcriptomics studies.

Transcriptomic Approach	Representative Biomarkers	Representative Studies	Main Findings	Key Limitations
Single-target mRNA assays	Survivin mRNA RT-PCR; *hTERT* mRNA template-ready PCR; preproGRP RT-PCR	Chen et al. [92] Chen et al. [94] Dong et al. [93] Lacroix et al. [97]	Single-gene mRNA assays improved diagnostic yield over sputum cytology; *hTERT* mRNA showed strong specificity compared with benign pulmonary controls; preproGRP showed limited sensitivity for SCLC	Sample size; heterogeneous studies; RNA integrity, sputum handling, and cellular composition likely influenced performance
Single ncRNA markers	miR-223; circ_0006949	Bagheri et al. [95] Bai et al. [96]	Promising discriminatory ability; miR-223 and circ_0006949 showed specificity for NSCLC	Small and exploratory studies; limited external validation; risk of overfitting
Histology-oriented miRNA panels	Four-miRNA adenocarcinoma panel (miR-486, miR-21, miR-200b, miR-375); three-miRNA squamous cell carcinoma panel (miR-205, miR-210, miR-708)	Yu et al. [98] Xing et al. [99]	Panels outperformed single target assays; consistent across training and validation cohorts; potential for sputum-based histologic subtyping	Hospital-based case–control cohorts with diagnostically enriched populations; broad applicability uncertain
General diagnostic miRNA panels	Five-miRNA, three-miRNA, and two-miRNA digital PCR panels	Roa et al. [100] Razzak et al. [101] Li et al. [102]	miRNA panels had high sensitivity and specificity for NSCLC; digital PCR more precise and feasible for sputum-based quantification	Performance varied across platforms, panel composition, and disease stage; small sample size; internal validation only
Integrated RNA plus imaging approaches	miR-31/miR-210 panel with CT; three-miRNA panel for indeterminate SPN	Shen et al. [103] Xing et al. [104]	Sputum RNA biomarkers combined with CT improved sensitivity and specificity for SPN triage and diagnosis	Independent, external validation awaited; impact on clinical outcomes remains to be demonstrated
snoRNA and broader ncRNA panels	snoRD66/snoRD78; five-ncRNA panel; miR-145/miR-126/miR-7 panel	Su et al. [106]	snoRNA and mixed ncRNA panels had moderate to strong diagnostic performance; improved specificity when combined with CT	Small sample size; limited external validation; risk of overfitting

Abbreviations: AUROC, area under the receiver operating characteristic curve; CT, computed tomography; *GRP*, gastrin-releasing peptide; *hTERT*, human telomerase reverse transcriptase; LC, lung cancer; LDCT, low-dose computed tomography; mRNA, messenger ribonucleic acid; miRNA, micro-ribonucleic acid; ncRNA, non-coding ribonucleic acid; NSCLC, non–small cell lung cancer; PCR, polymerase chain reaction; RNA, ribonucleic acid; RT-PCR, reverse transcription polymerase chain reaction; SCLC, small cell lung cancer; snoRNA, small nucleolar ribonucleic acid; SPN, solitary pulmonary nodule.

#### 3.4.6. Metabolomics

Sputum metabolomics studies, totaling 10 in number (see Appendix A.6), were predominantly exploratory case–control investigations using mass spectrometry (MS), nuclear magnetic resonance (NMR), or spectroscopy platforms to identify discriminatory metabolic signatures for LC detection, with most evidence still at the biomarker-discovery stage (see Table 6). Early feasibility work by Ahmed et al. [108] showed that metabolomic profiling of induced sputum and exhaled breath condensate (EBC) was technically feasible in advanced NSCLC, with reduced methanol in EBC and a possible signal from absent sputum glucose, although the study was very small and limited to stage III–IV disease. Later longitudinal work from the same group [109,110] examined metabolic shifts before and after surgical resection in early-stage NSCLC, identifying postoperative changes in sputum metabolites such as glucose, adenosine monophosphate, and N1,N12-diacetylspermine, thereby supporting biologic tumor-related metabolic reversibility, but these studies were not designed to establish diagnostic accuracy. Cross-sectional discovery studies suggested that sputum metabolic patterns can distinguish cancer from controls: Cameron et al. [111] found clear metabolomic separation between LC cases and healthy controls, with several metabolites, including ganglioside GM1, achieving AUROCs above 0.8. Similarly, Zhang et al. [112] reported altered sputum phospholipids in NSCLC, with lower dipalmitoyl phosphatidylcholine and higher phosphatidylglycerol and phosphatidylglycerol phosphate in cancer than in controls, while Gao et al. [113] also observed clear separation of cancer and healthy groups by ND-EESI-MS, with phosphatidylcholines as key discriminators. Among the more mature metabolomic studies, Zheng et al. [114] developed a five-metabolite sputum panel for lung adenocarcinoma using ND-EESI-MS and reported an AUROC of 0.917, with 90% sensitivity and 80% specificity, supported by internal hold-out validation and pathway analyses implicating sphingolipid, fatty acid, and glycolytic metabolism. Other studies were less directly diagnostic: Lewis et al. [115] showed proof-of-concept separation of LC and healthy controls by FTIR spectral features, while Ardatskaya et al. [116] found increased sputum short-chain fatty acids in LC and related respiratory disease states, suggesting altered airway microbial metabolism but without formal cancer diagnostic metrics. Overall, sputum metabolomics appears biologically informative and potentially capable of discriminating LC from non-cancer states, but the literature remains limited by small discovery cohorts, case–control enrichment, mixed control populations, variable platforms, and sparse external validation.

#### 3.4.7. Metagenomics and Microbiomics

Sputum microbiomic and metagenomic studies (total studies: 19) were generally exploratory and heterogeneous in purpose, spanning LC detection, histologic stratification, metastatic phenotyping, and treatment-response prediction (see Table 7 and Appendix A.8). In diagnostic case–control settings, several 16S-based studies showed that sputum microbial community structure differed between LC and non-cancer groups, although global diversity findings were inconsistent, and few studies reported clinically usable diagnostic metrics. Baranova et al. [117] found significant beta-diversity differences between squamous cell lung carcinoma and controls, with enrichment of Firmicutes, Streptococcus, Bacillus, Gemella, and Haemophilus in cancer sputum, and *Streptococcus agalactiae* emerged as the most prominent species-level signal, although no AUROC, sensitivity, or specificity estimates were provided. Earlier pilot metagenomic work by Cameron et al. [118] also suggested enrichment of *Streptococcus viridans* and *Granulicatella adiacens* in cancer-associated sputum, but interpretation was limited by the extremely small sample size. Druzhinin et al. [119] similarly reported increased Haemophilus and Bergeyella in LC sputum in one study and increased Streptococcus, Bacillus, Gemella, and Haemophilus in a larger follow-up cohort [120], alongside higher chromosomal aberration and micronucleus frequencies in peripheral lymphocytes, suggesting a possible link between airway dysbiosis and systemic genomic instability.

Some studies suggested that sputum microbiome patterns may vary by histology or disease extent rather than simply by cancer presence. Druzhinin et al. [121] reported that sputum microbiome alterations were more evident in squamous cell carcinoma than adenocarcinoma, with Streptococcus, Bacillus, Peptostreptococcus, Prevotella, Rothia, and Actinobacillus enriched in squamous cell carcinoma, whereas adenocarcinoma showed no significant differences from healthy donors. Likewise, Huang et al. [122] found that, in NSCLC, Granulicatella and Actinobacillus were enriched in early-stage disease, Actinomyces in advanced disease, Peptostreptococcus in intrathoracic metastasis, and Parvimonas in lymph node metastasis and *EGFR*-mutant adenocarcinoma, supporting a clinicopathologic association of sputum microbiota with tumor phenotype. Lu et al. [123] further showed that sputum microbiota was more strongly associated with NSCLC and distant metastasis than gut microbiota, with sputum-only random-forest models achieving AUROCs of 0.750 for control versus NSCLC, 0.850 for control versus stage I–III disease, and 0.720 for brain versus non-brain metastasis; Pseudomonas was particularly enriched in patients with brain metastases.

Evidence for clinically actionable sputum microbiome biomarkers was strongest when bacterial markers were integrated with other analytes or when microbiota were studied in relation to treatment outcomes. Dhilipkannah et al. [124] reported that combining sputum bacterial DNA markers with circulating plasma miRNAs produced an integrative panel with 87% sensitivity and 89% specificity for LC detection, outperforming the individual biomarker classes alone and maintaining performance in an independent validation cohort. In the immunotherapy setting, Zhang et al. [125] found that baseline sputum microbiota showed moderate ability to predict anti–PD-1 response in metastatic NSCLC, with Streptococcus yielding an AUROC of 0.77 and correlating with tumor CD8+ T-cell density, while Zapata-García et al. [126] observed that immune checkpoint inhibitor responders had higher airway alpha diversity, lower Firmicutes and Streptococcus, and greater abundance of Fusobacterium and Porphyromonas; in that cohort, Gemella predicted non-response, and Lachnoanaerobaculum predicted response. Overall, sputum microbiomics research suggests that airway dysbiosis is biologically linked to LC presence, histology, metastatic behavior, and possibly immunotherapy response, but the field remains largely hypothesis-generating, because most studies were single-center, cross-sectional, or case–control, often lacked external validation, and rarely produced standardized, transportable diagnostic models.

**Table 7:** Overall summary of sputum-based metagenomics and microbiomics studies.

Metagenomic Approaches	Representative Biomarkers	Representative Studies	Main Findings	Key Limitations
Exploratory diagnostic microbiome profiling	16S rRNA shotgun sequencing; microbial community composition and β-diversity analyses	Baranova et al. [127] Cameron et al. [118] Druzhinin et al. [119]	Sputum microbial composition differed between LC and non-cancer controls; airway dysbiosis accompanies LC	Small, single-center, and exploratory studies; global diversity findings were inconsistent; performance metrics missing in many studies
Histology-specific microbiome stratification	Subtype-focused sputum bacterial profiling in squamous cell carcinoma versus adenocarcinoma	Druzhinin et al. [121] Baranova et al. [117]	Microbiome alterations more pronounced in squamous cell carcinoma than in adenocarcinoma	Hypothesis-generating evidence based on cross-sectional study; external, independent validation needed
Stage, metastasis, and molecular phenotype associations	Association analyses linking sputum microbiota to early versus advanced stage, metastatic pattern, and *EGFR* mutation status	Huang et al. [122] Lu et al. [123]	Within NSCLC, sputum microbial patterns were associated with stage, metastasis, and molecular alterations	Biological feasibility studies; confounding bias possible; needs larger scale validation
Machine-learning diagnostic models	Random-forest models based on sputum microbial signatures	Lu et al. [123]	Machine-learning models afforded fair discriminatory performance (AUROC 0.75) for NSCLC	Limited external validation; uncertain reproducibility across populations and sequencing pipelines
Integrated multi-analyte biomarker panels	Sputum bacterial DNA markers combined with circulating plasma miRNAs	Dhilipkannah et al. [124]	Combined sputum DNA and plasma miRNA panel had 87% sensitivity and 89% specificity	Impact on patient outcomes uncertain; independent validation needed
Immunotherapy response prediction	Baseline sputum microbiota as predictors of anti-PD-1 ICI response	Zhang et al. [128] Zapata-Garcia et al. [126]	Higher airway α-diversity and enrichment of certain taxa associated with response to anti-PD-1 ICI therapy	Early-phase, retrospective, exploratory evidence; confounding bias possible; clinical utility remains to be demonstrated

Abbreviations: AUROC, area under the receiver operating characteristic curve; DNA, deoxyribonucleic acid; *EGFR*, epidermal growth factor receptor; ICI, immune checkpoint inhibitor; LC, lung cancer; mRNA, messenger ribonucleic acid; miRNA, micro-ribonucleic acid;;NSCLC, non–small cell lung cancer; PD-1, programmed cell death protein-1; rRNA, ribosomal ribonucleic acid.

#### 3.4.8. Integromics/Multi-Omics Approaches

Sputum integromics (multi-omics) studies consistently suggested that combining biomarker classes improves performance over single-analyte approaches, particularly for early detection and nodule triage (see Table 8 and Appendix A.7). Early integrative work combined genetic and epigenetic alterations in sputum, showing that multi-marker panels could detect tumor-related abnormalities even when cytology was negative. Hsu et al. [129] selected a seven-marker panel comprising microsatellite instability/loss of heterozygosity and promoter methylation (D9S942, D9S286, GATA49D12, D13S170, *p16*, *RARβ*) and reported 82% sensitivity and 75% specificity for identifying cancer cells in cytologically negative sputum, with one high-risk control later developing LC. In a prospective CT screening cohort of cancer-free heavy smokers, Baryshnikova et al. [130] found sputum molecular abnormalities in 6.9% of participants, most commonly p16^INK4A^ methylation, indicating that integrated molecular screening could detect preclinical airway field changes, although predictive value for future cancer remained limited, because few cancers developed during follow-up.

The strongest contemporary evidence came from quantitative multi-omics panels that integrated sputum biomarkers with either plasma analytes or radiologic variables. Su et al. [131] showed that combining sputum miRNAs (miR-31, miR-210) with sputum methylation markers (*RASSF1A*, *3OST2*) produced a four-biomarker panel with 87.3% sensitivity and 90.4% specificity in training and 87.5% sensitivity and 89.5% specificity in validation for stage I NSCLC among smokers with CT-detected benign versus malignant nodules, clearly outperforming sputum cytology. Li et al. [82] extended this concept across biospecimens using microplate ddPCR, integrating sputum miRNAs and methylated DNA with plasma miRNAs; their panel achieved about 92% sensitivity and 92% specificity in both development and validation cohorts. Similarly, Liao et al. [132] combined plasma miRNAs, sputum *RASSF1A* methylation, and clinical-radiologic predictors such as smoking pack-years and nodule diameter into an integromic signature for LDCT-detected pulmonary nodules, yielding an AUROC of 0.97 with 90% sensitivity and 94% specificity in validation. In the screening setting, Carozzi et al. [133] integrated sputum and plasma molecular biomarkers with LDCT and showed that adding the biomarker panel improved LDCT specificity from 74% to 89% and positive predictive value from 4.3% to 10.6% while maintaining 90% sensitivity, supporting a role for multi-omics triage of screen-positive individuals. Overall, sputum integromics appears to offer the most clinically promising performance among molecular approaches, particularly when multiple analyte classes and imaging features are combined, but most studies still used enriched case–control or nodule-clinic populations and require prospective validation in true screening workflows.

**Table 8:** Overall summary of sputum-based integromics/multi-omics studies.

Integromics Approach	Representative Biomarkers	Representative Studies	Main Findings	Key Limitations
Multimodality sputum assessment	Combined sputum cytopathology (DNA cytometry; FISH for LOH and MSI), genomics (*K-RAS* and *TP53* mutations), methylomics (*p16^INK4a^* and *RASSF1A* promoter hypermethylation), proteomics (*MAGE* A1-A6) and transcriptomics (miR-31, miR-210)	Kersting et al. (2000) [134] Baryshnikova et al. (2008) [130] Shin et al. (2012) [135] Su et al. (2016) [131]	Combining multiple sputum-based biomarkers improves upon the performance of conventional cytology; performance metrics suggest strong sensitivity, specificity, and overall accuracy in nested case–control studies	Although stronger than conventional approaches, assays relied on targeted biomarkers/candidates selected from earlier work; nested case–control designs with unclear applicability to real-world cohorts
Sputum LB combined with blood-based LB	Microplate ddPCR quantification of multiple sputum miRNAs, sputum DNA methylation, and plasma miRNAs	Li et al. (2021) [82]	A 96-well ddPCR workflow simultaneously quantified many candidate targets and identified an integrated biomarker panel spanning sputum and plasma that outperformed single biomarker classes for early LC diagnosis, with reproducible validation in an independent cohort	Retrospective case–control design; applicability to real-world screening cohorts remains unclear
Sputum LB in conjunction with imaging	ITALUNG biomarker panel (IBP) combined with sputum cytopathology and LDCT results	Carozzi et al. (2017) [133]	The IBP showed very high positivity among baseline screen-detected LC; when combined with LDCT, IBP improved specificity and positive predictive value relative to single-test screening	IBP alone had lower specificity when used alone; simulation-based extrapolation used for multimodal performance metrics; further validation needed in real-world screening cohorts
True multi-omics (clinical, imaging, blood, and sputum) approach	Clinical variables (e.g., smoking), imaging (e.g., SPN), sputum LB (e.g., microbial signals, methylomics) and plasma LB (e.g., ncRNA profile)	Liao et al. (2024) [132]	This mature “integromic” framework (molecular signals from plasma and sputum combined with radiologic and clinical predictors) could distinguish malignant from benign LDCT-detected nodules with sufficient specificity, outperforming other approaches	Low sensitivity for stage I disease; impact on patient outcomes needs to be validated in real-world screening cohorts

Abbreviations: ddPCR, droplet digital polymerase chain reaction; DNA, deoxyribonucleic acid; FISH, fluorescent in situ hybridization; IBP, ITALUNG biomarker panel; *K-RAS*, Kirsten rat sarcoma viral oncogene homolog; LB, liquid biopsy; LDCT, low-dose computed tomography; LOH, loss of heterozygosity; *MAGE-A*, melanoma-associated antigen A family; MSI, microsatellite instability; ncRNA, non-coding ribonucleic acid; *p16^INK4a^*, inhibitor of cyclin-dependent kinase–4 family, 16 kDa protein; p53, tumor protein 53; *RASSF1A*, Ras association domain family 1 isoform A; SPN, solitary pulmonary nodule.

#### 3.4.9. Other: Laboratory Techniques and Sputum Processing

Studies in this category (see Appendix A.9) were largely methodological and focused on improving specimen adequacy, preserving analytes, and enriching diagnostically relevant material rather than directly evaluating cancer discrimination. Early proof-of-concept cell enrichment studies showed that automated or flow-based approaches could substantially increase the proportion of target respiratory or malignant cells in sputum: Frost et al. [136] reported 7.8-fold enrichment of neoplastic cells using fluorescence parameters alone and 10.5-fold enrichment when fluorescence was combined with light scatter, while Kraemer et al. [137] increased diploid respiratory epithelial cell purity from 1.1% to 42%, corresponding to approximately 38-fold enrichment, thereby facilitating downstream molecular analysis. Pre-analytic handling was also shown to materially affect sputum assay quality. Gottschall et al. [138] demonstrated that nuclear morphometric measurements were highly sensitive to fixative choice and environmental exposure, with formalin-containing BD CytoRich Red^™^ (Becton Dickinson, Inc.; Franklin Lakes, NJ, USA) outperforming alcohol-based Saccomanno fixative by minimizing temperature- and sunlight-related artifacts; in contrast, sunlight-exposed Saccomanno-fixed samples often became unevaluable for computer-assisted image analysis. van der Drift et al. [139] further showed that, while cfDNA is detectable in all sputum samples, bulk cfDNA quantity is heavily confounded by neutrophilic airway inflammation and COPD, and therefore is not itself a useful discriminator of LC, underscoring the importance of separating tumor-specific signals from inflammatory background. More recent studies extended sputum processing advances to newer analyte classes: Bano et al. [140] showed that exosomes can be reproducibly isolated from sputum and that exosomes from LC patients were significantly larger and quantitatively different than those from smokers and healthy controls, supporting sputum as a technically feasible source for EV biomarker work. Likewise, Ma et al. [141] reported that optimized preservation of cytological supernatants enabled combined cfDNA/cfRNA analysis across several sample types, with sputum achieving a 100% test success rate and 95% sensitivity in the sputum subgroup, suggesting that appropriate mucolysis, fractionation, and nucleic acid stabilization can make sputum suitable for dual-analyte molecular testing. Overall, these studies indicate that sputum processing is a major determinant of downstream biomarker performance, with the most useful strategies being those that enrich epithelial/tumor-derived components, preserve nucleic acid integrity, and reduce artifactual effects from fixation and inflammatory contamination.

## 4. Discussion

This scoping review demonstrated that sputum LB for LC is a broad and evolving field with a substantial historical foundation and increasing molecular sophistication, but is not yet clinically mature. The included literature spans nearly five decades, beginning with cytopathologic approaches and progressing toward genomic, epigenomic, proteomic, transcriptomic, microbiomic, metabolomic, and integrative multi-omics strategies. This trajectory reflects both technological progress and a conceptual shift in how sputum is understood: it is no longer considered to be merely a cytologic specimen, but a complex and potentially informative biospecimen that may capture host, tumor, and microenvironmental biology in a noninvasive manner. However, most available data remain exploratory, retrospective, and diagnostically enriched; accordingly, encouraging biomarker signals should presently be interpreted as evidence of biologic potential rather than proof of stand-alone clinical readiness. An overall cross-domain summary of the key findings of this scoping review can be found in Supplementary Table S1.

### 4.1. Key Findings

A key finding of this review is that the field of sputum-based LB remains overwhelmingly centered on diagnosis and early detection. More than three-fourths of studies addressed diagnosis (78%), and more than a quarter were also relevant to screening (30%). A small proportion of studies were potentially relevant for prognostication (14%) and detection of AGAs (15%). By contrast, relatively few studies examined histologic subtyping (5%), surveillance (2%), or response monitoring (6%). This imbalance suggests that sputum LB research is still predominantly positioned in the discovery and early clinical validation phases rather than in mature implementation across the continuum of LC care. Given the low procedural burden of sputum collection and its potential suitability for repeated sampling, the limited literature on longitudinal monitoring represents an important gap.

The dominance of methylomics within the current evidence base is also notable. Among all molecular domains, methylation-based approaches appear to have gained the most traction. This likely reflects both biological and technical advantages. Epigenetic alterations may arise early in carcinogenesis, including in the setting of field cancerization, and are often detectable using relatively sensitive and scalable assays. In contrast, transcriptomic, proteomic, and metabolomic biomarkers may provide richer biological resolution but are highly susceptible to pre-analytic instability, matrix effects, and batch variation. The prominence of methylomics in sputum therefore likely reflects a combination of biological relevance and practical assay feasibility.

The overall translational readiness of sputum-based biomarkers for LC clinical care are summarized in Table 9. In line with NCI EDRN’s five-phase biomarker development framework, sputum-based cytopathologic markers (e.g., CyPath^®^; bioAffinity Technologies, Inc.; San Antonio, TX, USA), ddPCR gene panels, ddMSP methylomics panels, miRNA panels, and proteomic-based approaches appear to be the most mature translationally. Nevertheless, it should be noted that, for most domains, the available evidence still supports adjunctive clinical development rather than routine stand-alone implementation of sputum LB.

### 4.2. Research Gaps and Barriers to Translation

Our review identified several persistent barriers to translation. The first is methodological heterogeneity; included studies varied widely in sputum collection method, whether spontaneous or induced samples were used, which specimen fractions (whole sputum, cellular pellet, or supernatant cfDNA/EV-rich fractions) were analyzed, how samples were processed and stored, what analytes were measured, and how positivity thresholds were defined. This heterogeneity makes direct comparison difficult and complicates efforts to identify which biomarker classes are genuinely the most promising. It also raises the possibility that some variation in performance across studies may reflect differences in specimen handling and assay workflow rather than true biological differences. Future studies should operationalize standardization more explicitly by prespecifying at least the following elements: collection method; time from collection to stabilization; mucolysis, centrifugation, and storage conditions; fraction selection; specimen adequacy and tumor enrichment criteria; extraction chemistry and internal controls; and locked assay thresholds with predefined quality-failure rules. Without harmonization of these pre-analytic and analytic variables, there is no conclusive way to determine if apparent differences between biomarkers are related to workflow variability or true biologic superiority.

The second major barrier is the predominance of higher-risk study designs. Most studies were judged to be at moderate, moderate-high, or high risk of bias, while only a small minority were clearly low-risk. This pattern is reflected by the preponderance of retrospective case–control studies, often using known LC cases (usually advanced stage) and healthy selected controls. Such designs may be useful for identifying candidate signals, but they tend to overestimate performance relative to real-world clinical populations such as screening cohorts, patients with indeterminate pulmonary nodules, symptomatic individuals undergoing workup, and patients with benign inflammatory or smoking-related lung disease. Clinically relevant validation should therefore move away from healthy control comparisons and instead test sputum assays in populations that mirror actual decision points in practice, such as heavy smokers with COPD or smoking-related interstitial lung disease.

A third weakness is the incomplete reporting of diagnostic performance. Sensitivity, specificity, and AUROC were not reported uniformly in all studies, and negative or positive predictive values were selectively reported. Without consistent reporting of clinically meaningful performance metrics, it is difficult to benchmark sputum assays against established standards or to determine where they might fit in the diagnostic pathway. Future validation studies should therefore report not only discrimination metrics, but also intended-use populations, threshold pre-specification, calibration, validation methods, clinically meaningful endpoints, and comparator strategies relevant to clinical practice—such as incremental value beyond LDCT and clinical risk prediction, nodule triage accuracy, reduction in unnecessary invasive procedures, and reproducibility on repeat sampling.

### 4.3. Implications of Available Evidence

Despite the limitations and gaps identified by this review, the literature provides several reasons for cautious optimism. First, the cumulative volume of studies across multiple domains suggests that sputum does contain biologically relevant LC signals. Second, the progression from single-marker assays to panels and, more recently, to integrative multi-omics approaches indicates increasing recognition that no single analyte class is likely to be sufficient across all clinical contexts. Third, the emergence of microbiome/metagenomic and integromic studies suggests that the field is moving toward more systems-level models of disease detection and characterization, which may ultimately prove more informative than isolated biomarkers alone. At the same time, these observations should not be misconstrued as justification for universal simultaneous multi-omics testing in all patients; rather, they support rational, staged integration within clearly defined clinical pathways.

**Figure 8:**
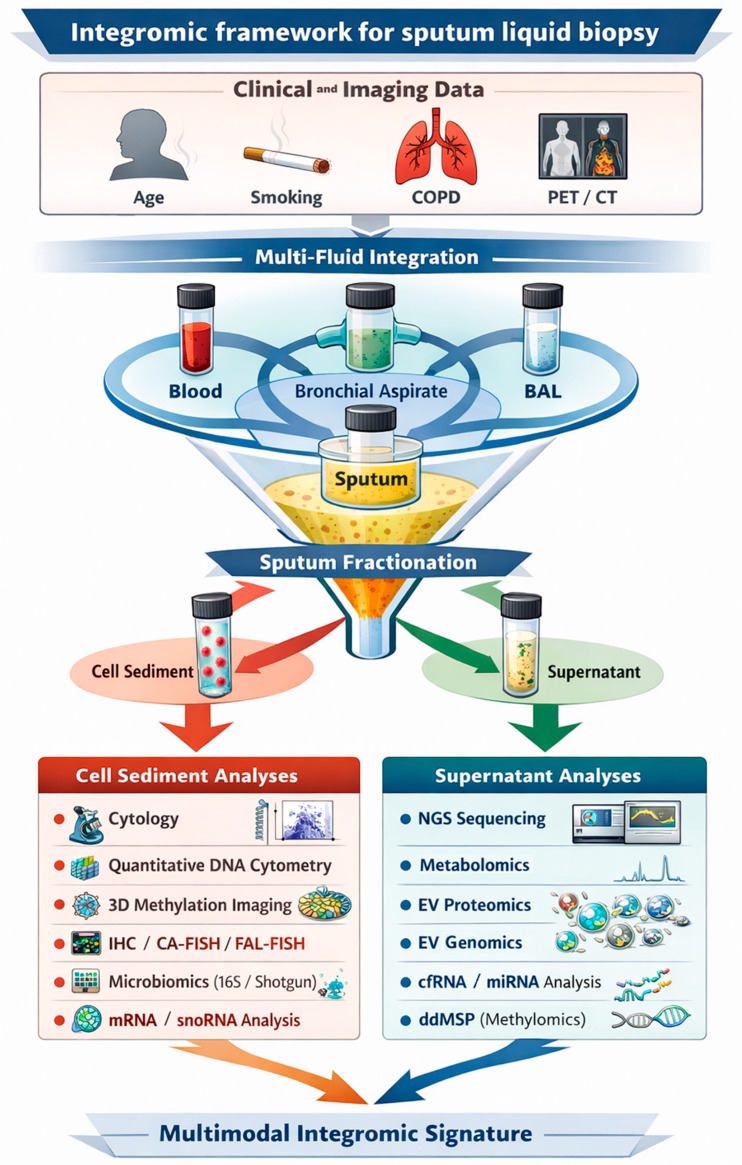
Integromic framework for sputum liquid biopsy. A schematic diagram showing the integromic framework of sputum liquid biopsy, which emphasizes multimodal sputum assessment combined with other biospecimen analyses (such as blood, bronchial aspirate, and/or bronchoalveolar lavage), imaging features, and clinical data to arrive at a personalized integromic signature. Such an integromic/multi-omics approach is unlikely to be practical or needed in every patient, but may be useful in selected, challenging cases where standard clinical workflows are deemed insufficient. Abbreviations: BAL, bronchoalveolar lavage; CA-FISH, chromosomal aneusomy–fluorescence in situ hybridization; COPD, chronic obstructive pulmonary disease; CT, computed tomography; cfDNA, cell-free deoxyribonucleic acid; cfRNA, cell-free ribonucleic acid; ddMSP, droplet digital methylation-specific polymerase chain reaction; DNA, deoxyribonucleic acid; EV, extracellular vesicles; FAL-FISH, fractional allele loss–fluorescence in situ hybridization; miRNA, micro-ribonucleic acid; mRNA, messenger ribonucleic acid; NGS, next-generation sequencing; PET, positron emission tomography; snoRNA, small nucleolar ribonucleic acid.

A putative framework for a comprehensive sputum LB is provided in Figure 8. Based on the 2026 NCCN guidelines, all patients with NSCLC require comprehensive biomarker profiling, including PD-L1 status, *EGFR* mutations, *ALK* gene fusion, *BRAF* V600E mutation, *K-RAS* G12C mutation, *ROS1* gene fusion, *HER2* (*ERBB2*) mutation, *NTRK* gene fusions, *MET* exon 14 skipping mutation, *RET* gene fusion, and *NRG1* gene fusion [142]. Sputum is a biologically proximal respiratory specimen that could potentially provide invaluable information regarding the tumor microenvironment. However, given the heavy biological noise in sputum from inflammatory cells and contamination from the upper aerodigestive tract, identifying relevant signals in this specimen is akin to finding a needle in a haystack. Such an elusive goal can only be achieved by standardization of sputum collection and processing methods, use of high-resolution molecular techniques, integration of molecular data from other specimens (if available), and incorporation of clinical and radiological data. In practical and realistic terms, a stepwise testing approach seems most plausible, rather than a universal simultaneous integration strategy. For instance, an initial clinical and radiologic risk assessment may identify a subgroup of patients where sputum LB may provide incremental information that either cannot be acquired by other means or is technically challenging to obtain. In such a stepwise approach, sputum can either serve as an initial medium for LB or be used as a last resort when blood, pleural fluid, or bronchial washings are not available.

From a clinical standpoint, the most plausible near-term role for sputum LB is likely an adjunctive decision support test rather than a stand-alone modality. A realistic implementation pathway could be a two-step or reflex model. Patients would first undergo standard clinical assessment and LDCT or diagnostic imaging; among those with indeterminate pulmonary nodules, discordant imaging–clinical features, or inadequate tissue for molecular workup, a limited sputum assay—for example, automated cytometry or a small prespecified methylation/miRNA panel performed on a defined sputum fraction—could be used to refine risk estimation and guide escalation to biopsy, short-interval imaging, or plasma/tissue genotyping. Such a strategy may be simpler and potentially more scalable than concurrent multi-omics testing across sputum, plasma, imaging, and clinical data in every patient. The inadequacy of current LDCT screening for LC was highlighted by the DELUGE (Detecting Early Lung Cancer in the Mississippi Delta Cohort) trial, which showed that a substantial proportion of patients diagnosed with early-stage LC were ineligible for LDCT screening per 2021 USPSTF criteria [11]. The Nodify Lung^®^ (Biodesix, Inc.; Louisville, CO, USA) nodule risk assessment strategy utilizes blood-based proteomics to reduce unnecessary testing and improve early detection of LC [21]. Additionally, the Percepta^®^ (Veracyte, Inc.; San Francisco, CA, USA) test uses nasal swab–based transcriptomics in conjunction with machine learning to facilitate accurate nodule triage [143]. Results of the NIGHTINGALE trial are anticipated in 2028 and will provide an objective assessment of the impact of Percepta^®^ (Veracyte, Inc.; San Francisco, CA, USA) testing on actual patient outcomes [144]. In screening settings, sputum-based assays may potentially complement clinical-, imaging-, and blood-based risk stratification to refine risk assessment and nodule triage and inform subsequent diagnostic steps. In diagnostic clinical pathways, sputum LB may provide additional noninvasive information when imaging is indeterminate or invasive tissue sampling is difficult, inadequate, or unfeasible. Currently, ongoing studies are assessing the role of novel sputum cytometry (such as CyPath^®^ [bioAffinity Technologies, Inc.; San Antonio, TX, USA]), sputum NGS (such as OncoScreen Plus^®^ [Burning Rock Dx; Guangzhou, China]), sputum miRNA panels, sputum DNA methylation panels, and EV-based or four-dimensional proteomic approaches [40,145,146].

Overall, clinically meaningful validation of sputum should focus on real-world populations and pragmatic endpoints such as better benign-versus-malignant discrimination in indeterminate nodules, improved confidence when tissue sampling is difficult, and reduction in unnecessary invasive procedures without sacrificing cancer detection. Complementary airway-based liquid biopsy approaches such as EBC also warrant a brief mention. Although EBC was outside the predefined scope of this scoping review, recent reviews have highlighted its promise as a noninvasive, organ-specific medium for cfDNA and miRNA-based profiling, while emphasizing the same translational barriers of low analyte yield and lack of standardization [147,148]. Pilot and proof-of-concept EBC studies, including genome-wide and NGS-based miRNA analyses, suggest that EBC may ultimately develop as a complementary airway liquid biopsy platform rather than as a direct substitute for sputum [149,150,151].

### 4.4. Priorities for Future Research

This review also highlights concrete priorities for future research. Prospective cohort-based study designs should be favored over retrospective case–control comparisons, and the utility of sputum LB should be assessed in clinically relevant populations, such as LDCT screening cohorts, patients with indeterminate pulmonary nodules, and patients with benign inflammatory lung disease. Healthy controls should be avoided as a comparator group, as they are not representative of real-world populations undergoing screening or testing. Moreover, cohorts of LC cancers should be representative of all stages of LC, since spectrum bias can lead to overestimation of diagnostic performance. Sputum collection and processing protocols should be standardized and reported in sufficient detail to support reproducibility. External validation across centers, populations, smoking exposures, histologic subtypes, and disease stages is essential. Studies should explicitly define their target clinical use case and benchmark performance against clinically relevant comparators rather than healthy controls alone. Greater attention is also needed for underexplored endpoints such as recurrence detection, serial disease monitoring, prediction of therapeutic response, and reduction in unnecessary invasive diagnostic procedures. Moreover, there are no sputum-based studies published to date that assess *HER2* overexpression or alterations of *MET*, *RET*, *ROS1*, and *NTRK*, which signifies an area of unmet clinical need.

## 5. Conclusions

Based on the current scoping review, available evidence supports sputum LB as a promising but still methodologically heterogeneous field, with its strongest signal in early detection (screening) and diagnostic applications, particularly utilizing methylomic, genomic, cytopathologic, proteomic, and transcriptomic approaches. However, the literature remains limited by inconsistent reporting, variable pre-analytic methods, and predominantly moderate to high risk of bias. Sputum collection and processing protocols should be standardized and reported in sufficient detail to support reproducibility. External validation across centers, populations, smoking exposures, histologic subtypes, and disease stages is also essential. Progress toward clinical translation will require a shift from exploratory biomarker discovery to standardized, prospective, and clinically anchored validation studies. In this regard, clinical studies should focus on integromic (multi-omic) stepwise approaches that incorporate clinical and imaging data with available molecular data from tissue biopsy or other specimens and then apply sputum LB in a well-defined clinical context to provide incremental value in a simple, scalable, and realistic fashion. Isolated sputum testing is unlikely to capture tumor and patient heterogeneity, which would preclude application across the full spectrum of lung cancer care pathways. Lastly, future clinical research should also focus on currently underexplored endpoints, such as prediction of response to targeted therapy, longitudinal response monitoring, and surveillance after treatment.

## Figures and Tables

**Table 4:** Overall summary of sputum-based proteomics studies.

Domain	Representative Techniques	Representative Studies	Main Findings	Typical Setting	Key Limitations
Single-marker tumor protein assays	Induced sputum ELISA for CYFRA21-1, CEA, NSE, etc.	Hillas et al. [86]	CYFRA21-1 was ~7× higher in LC than COPD (86% sensitivity, 75% specificity)	Diagnostic discrimination in symptomatic or clinically suspected LC	Older, single-analyte assays; modest sample size; limited external validation
Exploratory combined protein panels	ELISA for multiple proteins e.g., VEGF, ICAM-1, *TNF*α, etc.	Bar-Shai et al. [87]	Inflammatory and tumor-related proteins differed significantly among LC, COPD, and healthy controls; combined biomarker score improved case discrimination	Pilot, diagnostic enrichment, case–control studies	Combined biomarker score remains exploratory (calibration missing; external validation uncertain); risk of overfitting
Protein expression markers in exfoliated sputum cells	Cell-block IHC for *MCM2*, *MCM7*, etc.	Pankkal et al. [88]	*MCM2* (80.3% sensitivity and 100% specificity) and *MCM7* (92.1% sensitivity and 100% specificity) augmented conventional cytology	Cytology-linked diagnostic workup using exfoliated sputum cells in suspected LC	Single-study evidence (limited sample size); specialized cytologic processing required
High-dimensional discovery proteomics	diaPASEF (MS) profiling across 527 sputum proteins	Arenas-De Larriva et al. [89]	An internally cross-validated sPLS-DA model discriminated LC from controls, with an AUROC of 0.97	Established or suspected LC compared with controls (case–control designs)	Small sample size; case–control designs; limited external validation
Secretome (EV)-linked predictive proteomics	Proteome analysis of NSCLC cell-line secretomes integrated with patient sputum	Böttger et al. [90]	Feasibility of response prediction (34 sputum-detectable proteins associated with response to cisplatin)	Prospective cohort with established NSCLC undergoing chemotherapy	Early phase evidence; clinical utility uncertain
Multiplex biosensor devices	Portable sputum biosensor based on multichannel organic electrochemical transistor technology	Zhang et al. [91]	Excellent performance (AUROC 0.931) in case–control cohort; potential for longitudinal monitoring	Case–control designs (LC cases and heavy smokers at risk)	Case–control, diagnostically enriched populations; real-world effectiveness yet to be established

Abbreviations: AUROC, area under the receiver operating characteristic curve; CEA, carcinoembryonic antigen; COPD, chronic obstructive pulmonary disease; CRP, C-reactive protein; CYFRA21-1, cytokeratin fragment 19; diaPASEF, data-independent acquisition, parallel accumulation, serial fragmentation; ELISA, enzyme-linked immunosorbent assay; *ICAM*, intercellular adhesion molecule; IHC, immunohistochemistry; LC, lung cancer; *MCM*, minichromosome maintenance; NSE, neuron-specific enolase; sPLS-DA, sparse partial least squares discriminant analysis; SCLC, small cell lung cancer; *TNF*α, tumor necrosis factor-alpha; *VEGF*, vascular endothelial growth factor.

**Table 6:** Overall summary of sputum-based metabolomics studies.

Study	Sample Size	Techniques	Main Findings	Key Limitations
Lewis et al. (2010)	50 sputum samples (25 LC and 25 controls)	FTIR spectroscopy of sputum cell pellets; fingerprint-region spectral features linked to glycogen, proteins, and nucleic-acid-associated bands	FTIR-based sputum profiling separated LC from control using a small set of discriminatory wavenumbers (metabolic fingerprinting feasibility)	Case–control design with non-representative healthy controls; data-driven feature selection; absence of external validation
Ahmed et al. (2016)	20 sputum specimens (10 NSCLC and 10 benign lung conditions)	^1^H-MRS/NMR of sputum and EBC to assess low–molecular weight metabolites such as glucose, methanol, acetate, propionate, lysine, and formate	Relative absence of glucose in sputum and lower methanol in EBC noted in patients with NSCLC (biological feasibility)	Tiny sample size; predominantly advanced-stage NSCLC; hypothesis-generating findings
Ardatskaya et al. (2016)	147 patients (60 LC, 21 LC + CAP, and 38 COPD) and 30 healthy controls	Gas-liquid chromatographic analysis of sputum SCFA: acetate, propionate, and butyrate fractions plus anaerobic index	SCFA profiling showed systematic differences across healthy controls, COPD, LC, and LC complicated by CAP	Mixed disease cohorts; diagnostic metrics not reported; lack of external validation; reproducibility unclear
Cameron et al. (2016)	Sputum from 34 suspected LC cases (16 confirmed) and 33 healthy controls	FIE-MS and GC-MS of gangliosides, polyamines, and lipid metabolites	Untargeted MS profiling identified sputum metabolites that distinguished LC from both healthy controls and symptomatic non-cancer patients	Case–control design; non-representative controls; possible confounding bias
O’Shea et al. (2016)	Sputum from 23 LC cases, 11 symptomatic patients, and 33 healthy volunteers	FIE-MS features integrated with ANN classifiers	Secondary modeling of sputum metabolomic data discriminated LC from control with excellent internally cross-validated diagnostic performance	Tiny sample size; case–control design; lack of external validation; potential overfitting
Zhang et al. (2016)	307 sputum samples (167 NSCLC and 140 controls)	ND-EESI-MS lipid fingerprinting of DPPC, PG, PGP, and related phospholipid species	NSCLC sputum showed lower relative abundance of DPPC and higher PG and PGP compared with controls	No diagnostic metrics reported; lack of external validation; reproducibility unclear; potential overfitting
Gao et al. (2018)	100 sputum samples (50 NSCLC and 50 controls)	ND-EESI-MS analysis of spontaneous sputum without extensive pretreatment	ND-EESI-MS identified sputum fingerprints that could differentiate NSCLC patients from healthy controls by PCA	Case–control design with diagnostically enriched population; possible confounding bias
Zheng et al. (2021)	143 spontaneous sputum samples (76 adenocarcinoma and 67 controls)	ND-EESI-MS with PLS-DA or OPLS-DA to assess hydroxyphenyllactic acid, phytosphingosine, N-nonanoylglycine, sphinganine, and S-carboxymethyl-L-cysteine	A five-metabolite sputum panel discriminated lung adenocarcinoma from controls with high accuracy; pathway analysis implicated sphingolipid metabolism, fatty-acid metabolism, carnitine synthesis, and the Warburg effect	Case–control design with diagnostically enriched population; lack of external validation; potential overfitting
Ahmed et al. (2022)	15 sputum specimens (2 squamous cell carcinoma; 13 adenocarcinoma)	Pre- versus post-surgical resection NMR and LC-QTOF-MS of sputum and EBC: lipids, purines, carnitines, glucose, acetate, propionate, AMP, and diacetylspermine	Numerous sputum and EBC metabolites changed after resection; potential utility for treatment-response assessment and recurrence surveillance; sputum changes included glucose, adenosine monophosphate, and N1,N12-diacetylspermine	Tiny sample size; confounding bias from surgery-related physiologic stress; hypothesis-generating findings

Abbreviations: AMP, adenosine monophosphate; ANN, artificial neural network; AUROC, area under the receiver operating characteristic curve; CAP, community-acquired pneumonia; CID, collision-induced dissociation; DPPC, dipalmitoyl phosphatidylcholine; EBC, exhaled breath condensate; FIE-MS, flow infusion electrospray ion mass spectrometry; FTIR, Fourier transform infrared spectroscopy; GC-MS, gas chromatography–mass spectrometry; LC-QTOF-MS, liquid chromatography quadrupole time-of-flight mass spectrometry; MRS, magnetic resonance spectroscopy; ND-EESI-MS, neutral desorption extractive electrospray ionization mass spectrometry; NMR, nuclear magnetic resonance (spectroscopy); NSCLC, non–small cell lung cancer; OPLS-DA, orthogonal partial least squares discriminant analysis; PCA, principal component analysis; PG, phosphatidylglycerol; PGP, phosphatidylglycerol phosphate; PLS-DA, partial least squares discriminant analysis; SCFA, short-chain fatty acid.

**Table 9:** Translational readiness of various biomarkers for clinical use in LC care.

Biomarker Category	Potential Clinical Use Cases	Candidate Biomarkers and/or Techniques	Translational Maturity *	Next Steps
Cytopathology	Diagnosis Screening Histologic subtyping Actionable alterations Prognostication	Flow cytometry	Phase 4 (prospective validation phase)	Evaluation in large, multicenter, prospective cohorts
		MACS	Phase 2 (assay standardization phase)	Assay standardization and further validation
		Papanicolaou smear	Phase 5 (ready for clinical use)	Combine with other approaches for clinical use
		Quantitative microscopy (LungSign^®^; Perceptronix Medical Inc.; Vancouver, BC, Canada), automated DNA cytometry, 3D morphologic cytometry (LuCED^®^; Vision Gate Inc.; Pheonix, AZ, USA), PWS microscopy	Phase 3 (retrospective validation phase)	Clinical validation and use in prospective screening cohorts
		Porphyrin labeling (CyPath^®^; bioAffinity Technologies, Inc.; San Antonio, TX, USA)	Phase 4–5 (prospective validation and early clinical use phase)	Follow results of NCT07168993
		FISH for MSI	Phase 3 (retrospective validation phase)	Assess performance in prospective cohorts
		CA-FISH panel	Phase 3 (retrospective validation phase)	
		*EGFR* copy number assessment	Phase 4 (prospective validation phase)	
		FAL-FISH panel	Phase 2–3 (assay development and retrospective validation phase)	Assay development and clinical validation
		TRAP	Phase 2 (assay development phase)	Assay development and clinical validation
Genomics	Diagnosis Screening Actionable alterations Monitoring response Prognostication	*EGFR* mutations	Phase 2–4 (assay development and clinical validation phase)	Assay standardization and clinical validation
		*BRAF* mutation	Phase 2 (assay development phase)	Assay development, standardization, and development
		*K-RAS* mutation	Phase 2–4 (assay development and clinical validation phase)	Assay standardization and clinical validation
		*TP53* mutation	Phase 2–4 (assay development and clinical validation phase)	Assay standardization and clinical validation
		*EML4*-*ALK* fusion	Phase 2–3 (assay development and clinical validation phase)	Assay development, standardization, and validation
		PD-L1 status	Phase 2 (assay development phase)	Assay development and standardization; clinical validation
		*HER2*, *ROS1*, *RET*, *MET*, *NTRK*, and *NRG* gene alterations	No sputum-based data	Discovery of sputum-based methods of detection
		Multiplex ddPCR panels	Phase 2 (assay development phase)	Assay development and standardization; clinical validation
		NGS profiling		
Methylomics	Diagnosis Screening Prognostication	ddMSP panels (*p16^INK4a^*, *RASSF1A*, *SOX17*, *TAC1*, etc.)	Phase 3–4 (clinical validation phase)	Clinical validation, evaluation in prospective cohorts, and impact on patient outcomes
		3D quantitative DNA topology imaging	Phase 2 (assay development phase)	Assay development, standardization, and calibration; clinical validation
Transcriptomics	Diagnosis Screening Subtyping Monitoring response Prognostication	Survivin mRNA *hTERT* mRNA	Phase 2 (assay development phase)	Assay development, standardization, and calibration
		Adenocarcinoma four-miRNA panel Squamous cell carcinoma three-miRNA panel	Phase 3 (clinical validation phase)	Clinical validation and evaluation in prospective cohorts
		snoRNA panel	Phase 2 (assay development phase)	Assay development, standardization, and calibration
Proteomics	Diagnosis Screening Histologic subtyping Actionable alterations Monitoring response Surveillance Prognostication	Cell block IHC for tumor markers	Phase 2–3 (assay development and clinical validation phase)	Clinical validation and evaluation in prospective cohorts
		Single and multiple protein panels (SELDI-TOF/MALDI-TOF and ELISA)	Phase 2–3 (assay development and clinical validation phase)	Assay development and standardization; clinical validation
		diaPASEF (MS) profiling	Phase 2 (assay development phase)	Assay development, standardization, and calibration
		EV-derived proteome profiling, similar to ExoDx™ Lung(ALK) [Exosome Diagnostics Inc.; Waltham, MA, USA]		
		Portable biosensors (multichannel electrochemical transistor technology)	Phase 3–4 (assay development and clinical validation phase)	Evaluation in prospective cohorts and assessment of impact on patient outcomes
Metabolomics	Diagnosis Screening Monitoring response Surveillance	FTIR spectroscopy, Raman spectroscopy, GC-MS, FIE-MS, and ND-EESI-MS for lipid fingerprinting, and glucose and glycolytic metabolites	Phase 2 (assay development phase)	Assay development, standardization, and calibration followed by clinical validation
Microbiomics	Diagnosis Histologic subtyping Response prediction Monitoring response Prognostication	16S rRNA sequencing for specific taxa (Gemella, Firmicutes, Bacillus, Granulicatella, etc.)	Phase 2 (assay development phase)	Assay development and calibration, clinical validation, and assessment in prospective cohorts

* Translational maturity as reflected by the current phase of biomarker development, based on the National Cancer Institute Early Detection Research Network’s five-phase framework. Abbreviations: *ALK*, anaplastic lymphoma kinase; *BRAF*, v-raf murine sarcoma viral oncogene, homolog B; CA-FISH, chromosomal aneusomy–fluorescence in situ hybridization; ddPCR, droplet digital polymerase chain reaction; diaPASEF, data-independent acquisition, parallel accumulation, serial fragmentation; DNA, deoxyribonucleic acid; *EGFR*, epidermal growth factor receptor; *EML4*, echinoderm microtubule-associated protein-like 4; FAL-FISH, fractional allele loss–fluorescence in situ hybridization; FIE-MS, flow infusion electrospray ion-mass spectrometry; FISH, fluorescent in situ hybridization; FTIR, Fourier transform infrared (spectroscopy); GC-MS, gas chromatography–mass spectrometry; *HER2*, human epidermal growth factor receptor 2; MACS, magnetic-activated cell sorting; MALDI-TOF, matrix-assisted laser desorption/ionization time-of-flight; mRNA, messenger ribonucleic acid; miRNA, micro-ribonucleic acid; MSI, microsatellite instability; ND-EESI-MS, neutral desorption extractive electrospray ionization mass spectrometry; *NRG*, neuregulin; *NTRK*, neurotrophic tyrosine receptor kinase; PWS, pulsed wave spectrometry; *RET*, rearranged during transfection proto-oncogene; rRNA, ribosomal ribonucleic acid; SELDI-TOF, surface-enhanced laser desorption/ionization time-of-flight; TRAP, telomerase repeat amplification protocol.

## Data Availability

The original contributions presented in this review are included in the article/Supplementary Materials. Further inquiries can be directed to the corresponding author.

## Supplementary Materials

The following supporting information can be downloaded at: https://www.mdpi.com/article/10.3390/medsci14020231/s1, Table S1. Overall summary and translational readiness of sputum biomarkers across all domains included in the scoping review.

## Abbreviations

The following abbreviations are used in this manuscript:

AGA	Actionable genomic alteration
AI	Artificial intelligence
ALK	Anaplastic lymphoma kinase
AMP	Adenosine monophosphate
ANN	Artificial neural network
APC	Adenomatous polyposis coli
ARMS	Amplification-refractory mutation system
AUC	Area under the curve
AUROC	Area under the receiver operating characteristic curve
BIOCROSS	Biomarker-based cross-sectional study
*BRAF*	v-raf murine sarcoma viral oncogene, homolog B
CA125	Cancer antigen 125
CA-FISH	Chromosomal aneusomy–fluorescence in situ hybridization
CAP	Community-acquired pneumonia
CD	Cluster of differentiation
CEA	Carcinoembryonic antigen
cfDNA	Cell-free deoxyribonucleic acid
cfRNA	Cell-free ribonucleic acid
CID	Collision-induced dissociation
circRNA	Circular ribonucleic acid
CoBRA	Combined bisulfite modification and restriction analysis
COPD	Chronic obstructive pulmonary disease
CRP	C-reactive protein
CT	Computed tomography
CTCs	Circulating tumor cells
ctDNA	Circulating tumor deoxyribonucleic acid
CYFRA21-1	Cytokeratin fragment 19
*CYGB*	Cytoglobin
ddMSP	Droplet digital methylation-specific polymerase chain reaction
ddPCR	Droplet digital polymerase chain reaction
DELUGE	Detecting Early Lung Cancer in the Mississippi Delta Cohort
diaPASEF	Data-independent acquisition, parallel accumulation, serial fragmentation (proteomics)
DNA	Deoxyribonucleic acid
DPAK	Death-associated protein kinase
DPPC	Dipalmitoyl phosphatidylcholine
EBC	Exhaled breath condensate
EDRN	Early Detection Research Network
*EGFR*	Epidermal growth factor receptor
ELISA	Enzyme-linked immunosorbent assay
*EML4*	Echinoderm microtubule-associated protein-like 4
*ENO1*	Enolase 1
EV	Extracellular vesicle
FAL-FISH	Fractional allele loss–fluorescence in situ hybridization
FDA	Food and Drug Administration
*FHIT*	Fragile histidine triad
FIE-MS	Flow infusion electrospray ion mass spectrometry
FISH	Fluorescence in situ hybridization
FTIR	Fourier transform infrared (spectroscopy)
GC-MS	Gas chromatography–mass spectrometry
*GRP*	Gastrin-releasing peptide
GLOBOCAN	Global Cancer Observatory
*HER2*	Human epidermal growth factor receptor 2
HSROC	Hierarchical summary receiver operating characteristic
*hTERT*	Human telomerase reverse transcriptase
*HYAL2*	Hyaluronidase 2
IBP	ITALUNG biomarker panel
*ICAM-1*	Intercellular adhesion molecule 1
ICI	Immune checkpoint inhibitor
JBI	Joanna Briggs Institute
*K-RAS*	Kirsten rat sarcoma viral oncogene homolog
LB	Liquid biopsy
LC	Lung cancer
LCCP	Lung Cancer Compact Panel^®^
LC-QTOF-MS	Liquid chromatography quadrupole time-of-flight mass spectrometry
LDCT	Low-dose computed tomography
LOH	Loss of heterozygosity
MACS	Magnetic-activated cell sorting
*MAGE-A*	Melanoma-associated antigen A family
MALDI-TOF	Matrix-assisted laser desorption/ionization time-of-flight
*MAP4*	Microtubule-associated protein 4
*MCM*	Minichromosome maintenance
*MGMT*	O^6^-methylguanine DNA methyltransferase
miRNA	Micro-ribonucleic acid
mRNA	Messenger ribonucleic acid
MRS	Magnetic resonance spectroscopy
MS	Mass spectrometry
MSI	Microsatellite instability
MSP	Methylation-specific polymerase chain reaction
MSRE	Methylation-specific restriction enzyme
NCCN	National Comprehensive Cancer Network
NCI	National Cancer Institute
ncRNA	Non-coding ribonucleic acid
ND-EESI-MS	Neutral desorption extractive electrospray ionization mass spectrometry
NGS	Next-generation sequencing
NLST	National Lung Screening Trial
NMR	Nuclear magnetic resonance
*NRG*	Neuregulin
NSCLC	Non–small cell lung cancer
NSE	Neuron-specific enolase
*NTRK*	Neurotrophic tyrosine receptor kinase
OPLS-DA	Orthogonal partial least squares discriminant analysis
p16^INK4a^	Inhibitor of cyclin-dependent kinase–4 family, 16 kDa protein
p53	Tumor protein 53
*PAX5*	Paired box 5
PCA	Principal component analysis
PCR	Polymerase chain reaction
PD1	Programmed cell death protein 1
PG	Phosphatidylglycerol
PGP	Phosphatidylglycerol phosphate
PLS-DA	Partial least squares discriminant analysis
PRISMA-ScR	Preferred Reporting Items for Systematic Reviews and Meta-Analyses—extension for Scoping Reviews
PWS	Pulsed wave spectrometry
QMSP	Quantitative methylation-specific polymerase chain reaction
QUADAS-2	Quality Assessment of Diagnostic Accuracy Studies
QUIPS	Quality in Prognostic Studies
*RAR*	Retinoic acid receptor
*RASSF1A*	Ras association domain family 1 isoform A
*RET*	Rearranged during transfection proto-oncogene
RNA	Ribonucleic acid
ROC	Receiver operating characteristic
RT-PCR	Reverse transcription polymerase chain reaction
SCFA	Short-chain fatty acids
SCLC	Small cell lung cancer
SELDI-TOF	Surface-enhanced laser desorption/ionization time-of-flight
*SERPINA1*	Serpin family A member 1
*SKP2*	S-phase kinase-associated protein 2
snoRNA	Small nucleolar ribonucleic acid
*SOX*	SRY-box transcription factor
sPLS-DA	Sparse partial least squares discriminant analysis
SPN	Solitary pulmonary nodule
*TAC1*	Tachykinin precursor 1
*TNF*	Tumor necrosis factor
TRAP	Telomerase repeat amplification protocol
*UGGT1*	UDP-glucose:glycoprotein glucosyltransferase 1
*VEGF*	Vascular endothelial growth factor

## Appendix A

A full list of research studies included in the final synthesis of this scoping review is provided here. There were 270 studies in total, which are categorized under one of the following headings:

### Appendix A.1. Cytopathology

- bioAffinity Technologies Inc. Detection of early-stage lung cancer in sputum using flow cytometry and an automated analysis pipeline. Available online: https://clinicaltrials.gov/study/NCT07168993 (accessed on 20 March 2026)
- Adonis, M.I.; Diaz, J.; Miranda, V.R.; Chahuan, M.; Zambrano, A.; Benitez, H.C.; Campos, M.; Avaria, P.; Urzua, U.; Marin, P.; et al. Biomarkers for screening of lung cancer and pre-neoplastic lesions in a high risk Chilean population. Biol Res 2014, 47, 62, doi:10.1186/0717-6287-47-62.
- Arvanitis, D.A.; Papadakis, E.; Zafiropoulos, A.; Spandidos, D.A. Fractional allele loss is a valuable marker for human lung cancer detection in sputum. Lung Cancer 2003, 40, 55–66, doi:10.1016/s0169-5002(02)00531-7.
- Auffermann, W.; Bocking, A. Early detection of precancerous lesions in dysplasias of the lung by rapid DNA image cytometry. Anal Quant Cytol Histol 1985, 7, 218–226.
- Baron, A.E.; Kako, S.; Feser, W.J.; Malinowski, H.; Merrick, D.; Garg, K.; Malkoski, S.; Pretzel, S.; Siegfried, J.M.; Franklin, W.A.; et al. Clinical Utility of Chromosomal Aneusomy in Individuals at High Risk of Lung Cancer. J Thorac Oncol 2017, 12, 1512–1523, doi:10.1016/j.jtho.2017.06.008.
- Bauta, W.; Grayson, M.; Titone, R.; Rebeles, J.; Rebel, V.I. Porphyrin-modified beads for use as compensation controls in flow cytometry. J Vis Exp 2023, doi:10.3791/65294.
- Bederka, L.H.; Sanchez, J.R.; Rebeles, J.; Araujo, P.R.; Grayson, M.H.; Lai, S.C.; DePalo, L.R.; Habib, S.A.; Hill, D.G.; Lopez, K.; et al. Sputum analysis by flow cytometry; an effective platform to analyze the lung environment. PLoS One 2022, 17, e0272069, doi:10.1371/journal.pone.0272069.
- Cao, L.; Zhai, Z.; Li, J.; Xu, M.; Xia, H.; Xu, X.; Ma, D.; Mei, X.; Cui, W. [The diagnostic value of flow cytometric DNA analysis of sputum in lung cancer]. Zhongguo Fei Ai Za Zhi 2004, 7, 202–205, doi:10.3779/j.issn.1009-3419.2004.03.05.
- Castagnaro, A.; Marangio, E.; Verduri, A.; Chetta, A.; D’Ippolito, R.; Del Donno, M.; Olivieri, D.; Di Cola, G. Microsatellite analysis of induced sputum DNA in patients with lung cancer in heavy smokers and in healthy subjects. Exp Lung Res 2007, 33, 289–301, doi:10.1080/01902140701539687.
- Cui, J.; Wang, X.; Rao, Y.; Ji, T.; Li, L. A new method for the sputum cytology test without direct contact to specimens during COVID-19 pandemic. Front Med (Lausanne) 2021, 8, 746731, doi:10.3389/fmed.2021.746731.
- Eerola, A.K.; Soini, Y.; Lehto, V.P.; Paakko, P. Increased numbers of alveolar macrophages with apoptotic bodies predict lung carcinoma. Apoptosis 1998, 3, 261–266, doi:10.1023/a:1009661209006.
- Fafin-Lefevre, M.; Morlais, F.; Guittet, L.; Clin, B.; Launoy, G.; Galateau-Salle, F.; Plancoulaine, B.; Herlin, P.; Letourneux, M. Nuclear morphology for the detection of alterations in bronchial cells from lung cancer: an attempt to improve sensitivity and specificity. Anal Quant Cytol Histol 2011, 33, 183–195.
- Fan, Y.G.; Hu, P.; Jiang, Y.; Chang, R.S.; Yao, S.X.; Wang, W.; He, J.; Prorok, P.; Qiao, Y.L. Association between sputum atypia and lung cancer risk in an occupational cohort in Yunnan, China. Chest 2009, 135, 778–785, doi:10.1378/chest.08-1469.
- Grainger, J.M.; Husain, O.A. Use of a mucolytic agent (Cytoclair) in the preparation of cell material for the detection of malignant cells in sputum. J Clin Pathol 1978, 31, 585–590, doi:10.1136/jcp.31.6.585.
- Guber, A.; Greif, J.; Rona, R.; Fireman, E.; Madi, L.; Kaplan, T.; Yemini, Z.; Gottfried, M.; Katz, R.L.; Daniely, M. Computerized analysis of cytology and fluorescence in situ hybridization (FISH) in induced sputum for lung cancer detection. Cancer Cytopathol 2010, 118, 269–277, doi:10.1002/cncy.20094.
- Jia, D.; Zhang, Z.; Liu, S. [Application of fluorescence in situ hybridization (FISH) in sputum cytologic diagnosis of lung cancer]. Zhonghua Zhong Liu Za Zhi 2000, 22, 477–479.
- Kang, J.U.; Koo, S.H.; Kwon, K.C.; Park, J.W.; Jung, S.S. Gain of the EGFR gene located on 7p12 is a frequent and early event in squamous cell carcinoma of the lung. Cancer Genet Cytogenet 2008, 184, 31–37, doi:10.1016/j.cancergencyto.2008.03.002.
- Katz, R.L.; Zaidi, T.M.; Fernandez, R.L.; Zhang, J.; He, W.; Acosta, C.; Daniely, M.; Madi, L.; Vargas, M.A.; Dong, Q.; et al. Automated detection of genetic abnormalities combined with cytology in sputum is a sensitive predictor of lung cancer. Mod Pathol 2008, 21, 950–960, doi:10.1038/modpathol.2008.71.
- Kemp, R.A.; Reinders, D.M.; Turic, B. Detection of lung cancer by automated sputum cytometry. J Thorac Oncol 2007, 2, 993–1000, doi:10.1097/JTO.0b013e318158d488.
- Kennedy, T.C.; Franklin, W.A.; Prindiville, S.A.; Cook, R.; Dempsey, E.C.; Keith, R.L.; Hirsch, F.R.; Merrick, T.A.; Shroyer, K.R.; Petty, T.L.; et al. High prevalence of occult endobronchial malignancy in high risk patients with moderate sputum atypia. Lung Cancer 2005, 49, 187–191, doi:10.1016/j.lungcan.2005.02.009.
- Kennedy, T.C.; Proudfoot, S.P.; Franklin, W.A.; Merrick, T.A.; Saccomanno, G.; Corkill, M.E.; Mumma, D.L.; Sirgi, K.E.; Miller, Y.E.; Archer, P.G.; et al. Cytopathological analysis of sputum in patients with airflow obstruction and significant smoking histories. Cancer Res 1996, 56, 4673–4678.
- Krivokuca, Z.; Tatomirovic, Z.; Cvetkovic, G.; Dzambas, J.; Skuletic, V.; Ristic, S. Validity of cytology in the diagnosis of small cell lung carcinoma. Vojnosanitetski pregled 2020, 77, 954–961, doi:10.2298/vsp180813172k.
- Li, G.; Guillaud, M.; LeRiche, J.; McWilliams, A.; Gazdar, A.; Lam, S.; MacAulay, C. Automated sputum cytometry for detection of intraepithelial neoplasias in the lung. Anal Cell Pathol (Amst) 2012, 35, 187–201, doi:10.3233/ACP-2012-0053.
- Lin, P.; Chen, Y.; Xu, J.; Huang, X.; Wen, W.; Zhang, L.; Kong, W.; Zhao, Z.; Ye, Y.; Bao, Z.; et al. A multicenter-retrospective cohort study of chromosome instability in lung cancer: clinical characteristics and prognosis of patients harboring chromosomal instability detected by metagenomic next-generation sequencing. J Thorac Dis 2023, 15, 112–122, doi:10.21037/jtd-22-1732.
- Mao, L.; Lee, D.J.; Tockman, M.S.; Erozan, Y.S.; Askin, F.; Sidransky, D. Microsatellite alterations as clonal markers for the detection of human cancer. Proc Natl Acad Sci U S A 1994, 91, 9871–9875, doi:10.1073/pnas.91.21.9871.
- McWilliams, A.; Mayo, J.; MacDonald, S.; leRiche, J.C.; Palcic, B.; Szabo, E.; Lam, S. Lung cancer screening. American Journal of Respiratory and Critical Care Medicine 2003, 168, 1167–1173, doi:10.1164/rccm.200301-144OC.
- Miozzo, M.; Sozzi, G.; Musso, K.; Pilotti, S.; Incarbone, M.; Pastorino, U.; Pierotti, M.A. Microsatellite alterations in bronchial and sputum specimens of lung cancer patients. Cancer Res 1996, 56, 2285–2288.
- Mondal, N.K.; Roychoudhury, S.; Ray, M.R. Higher AgNOR expression in metaplastic and dysplastic airway epithelial cells predicts the risk of developing lung cancer in women chronically exposed to biomass smoke. J Environ Pathol Toxicol Oncol 2015, 34, 35–51, doi:10.1615/jenvironpatholtoxicoloncol.2015010708.
- Palcic, B.; Garner, D.M.; Beveridge, J.; Sun, X.R.; Doudkine, A.; MacAulay, C.; Lam, S.; Payne, P.W. Increase of sensitivity of sputum cytology using high-resolution image cytometry: field study results. Cytometry 2002, 50, 168–176, doi:10.1002/cyto.10065.
- Pasrija, T.; Srinivasan, R.; Behera, D.; Majumdar, S. Telomerase activity in sputum and telomerase and its components in biopsies of advanced lung cancer. Eur J Cancer 2007, 43, 1476–1482, doi:10.1016/j.ejca.2007.04.003.
- Patriquin, L.; Merrick, D.T.; Hill, D.; Holcomb, R.G.; Lemieux, M.E.; Bennett, G.; Karia, B.; Rebel, V.I.; Bauer, T., 2nd. Early detection of lung cancer with meso tetra (4-carboxyphenyl) porphyrin-labeled sputum. J Thorac Oncol 2015, 10, 1311–1318, doi:10.1097/JTO.0000000000000627.
- Payne, P.W.; Sebo, T.J.; Doudkine, A.; Garner, D.; MacAulay, C.; Lam, S.; LeRichie, J.C.; Palcic, B. Sputum screening by quantitative microscopy: a reexamination of a portion of the National Cancer Institute Cooperative Early Lung Cancer Study. Mayo Clin Proc 1997, 72, 697–704, doi:10.4065/72.8.697.
- Prindiville, S.A.; Byers, T.; Hirsch, F.R.; Franklin, W.A.; Miller, Y.E.; Vu, K.O.; Wolf, H.J.; Baron, A.E.; Shroyer, K.R.; Zeng, C.; et al. Sputum cytological atypia as a predictor of incident lung cancer in a cohort of heavy smokers with airflow obstruction. Cancer Epidemiol Biomarkers Prev 2003, 12, 987–993.
- Qiu, Q.; Todd, N.W.; Li, R.; Peng, H.; Liu, Z.; Yfantis, H.G.; Katz, R.L.; Stass, S.A.; Jiang, F. Magnetic enrichment of bronchial epithelial cells from sputum for lung cancer diagnosis. Cancer 2008, 114, 275–283, doi:10.1002/cncr.23596.
- Rebel, V.; Bederka, L.; Araujo, P.; Sanchez, J.; Grayson, M.; Lemieux, M.; Rebeles, J.; Lai, S.; Reveles, X. Automated flow cytometry test distinguishes cancer from non-cancer in sputum with high sensitivity and specificity. In: Proceedings of the Journal of Thoracic Oncology, 2021; pp. S279-S280.
- Romeo, M.S.; Sokolova, I.A.; Morrison, L.E.; Zeng, C.; Baron, A.E.; Hirsch, F.R.; Miller, Y.E.; Franklin, W.A.; Varella-Garcia, M. Chromosomal abnormalities in non-small cell lung carcinomas and in bronchial epithelia of high-risk smokers detected by multi-target interphase fluorescence in situ hybridization. J Mol Diagn 2003, 5, 103–112, doi:10.1016/s1525-1578(10)60459-x.
- Saraswathy Veena, V.; Sara George, P.; Jayasree, K.; Sujathan, K. Comparative analysis of cell morphology in sputum samples homogenized with dithiothreitol, N-acetyl-L cysteine, Cytorich((R)) red preservative and in cellblock preparations to enhance the sensitivity of sputum cytology for the diagnosis of lung cancer. Diagn Cytopathol 2015, 43, 551–558, doi:10.1002/dc.23266.
- Sen, S.; Reddy, V.G.; Guleria, R.; Jain, S.K.; Kapila, K.; Singh, N. Telomerase--a potential molecular marker of lung and cervical cancer. Clin Chem Lab Med 2002, 40, 994–1001, doi:10.1515/CCLM.2002.173.
- Sen, S.; Reddy, V.G.; Khanna, N.; Guleria, R.; Kapila, K.; Singh, N. A comparative study of telomerase activity in sputum, bronchial washing and biopsy specimens of lung cancer. Lung Cancer 2001, 33, 41–49, doi:10.1016/s0169-5002(01)00193-3.
- Shlomi, D.; Peled, N.; Schwarz, Y.A.; Soo Hoo, G.W.; Batra, R.K.; Fink, G.; Kaplan, T.; Cohen, L.; Mollan, S.; Burfeind, W.R., Jr. Non-invasive early detection of malignant pulmonary nodules by FISH-based sputum test. Cancer Genet 2018, 226–227, 1–10, doi:10.1016/j.cancergen.2018.04.118.
- Tao, W.; Li, J.; Cheng, L.L. [Application of DNA-image cytometry in the diagnosis of lung cancer]. Zhonghua Zhong Liu Za Zhi 2016, 38, 113–117, doi:10.3760/cma.j.issn.0253-3766.2016.02.007.
- Tercelj, M.; Ales, A.; Rott, T.; Sever, N.; Prodnik, L.; Erzen, J.; Primic-Zakelj, M.; Rylander, R. DNA-based sputum cell image analysis for lung cancer in a clinical setting. Acta Cytol 2008, 52, 584–590, doi:10.1159/000325602.
- Varella-Garcia, M.; Kittelson, J.; Schulte, A.P.; Vu, K.O.; Wolf, H.J.; Zeng, C.; Hirsch, F.R.; Byers, T.; Kennedy, T.; Miller, Y.E.; et al. Multi-target interphase fluorescence in situ hybridization assay increases sensitivity of sputum cytology as a predictor of lung cancer. Cancer Detect Prev 2004, 28, 244–251, doi:10.1016/j.cdp.2004.04.007.
- Varella-Garcia, M.; Schulte, A.P.; Wolf, H.J.; Feser, W.J.; Zeng, C.; Braudrick, S.; Yin, X.; Hirsch, F.R.; Kennedy, T.C.; Keith, R.L.; et al. The detection of chromosomal aneusomy by fluorescence in situ hybridization in sputum predicts lung cancer incidence. Cancer Prev Res (Phila) 2010, 3, 447–453, doi:10.1158/1940-6207.CAPR-09-0165.
- Wang, F.; Zhang, Y.; Feng, Y.C.; Huang, Y.C. Comparison between FISH and cytology of sputum samples in detection of lung cancer. Journal of Practical Oncology 2013, 28, 197–199.
- Wang, X.; Cao, A.; Peng, M.; Hu, C.; Liu, D.; Gu, T.; Liu, H. The value of chest CT scan and tumor markers detection in sputum for early diagnosis of peripheral lung cancer. Zhongguo Fei Ai Za Zhi 2004, 7, 58–63, doi:10.3779/j.issn.1009-3419.2004.01.15.
- Xing, S.; Khanavkar, B.; Nakhosteen, J.A.; Atay, Z.; Jockel, K.H.; Marek, W.; Group, R.L.S. Predictive value of image cytometry for diagnosis of lung cancer in heavy smokers. Eur Respir J 2005, 25, 956–963, doi:10.1183/09031936.05.00118903.
- Yang, J.; Zhou, Y. Detection of DNA aneuploidy in exfoliated airway epithelia cells of sputum specimens by the automated image cytometry and its clinical value in the identification of lung cancer. J Huazhong Univ Sci Technolog Med Sci 2004, 24, 407–410, doi:10.1007/BF02861880.
- Zlobec, I.; Raineri, I.; Schneider, S.; Schoenegg, R.; Grilli, B.; Herzog, M.; Savic, S.; Bubendorf, L. Assessment of mean EGFR gene copy number is a highly reproducible method for evaluating FISH in histological and cytological cancer specimens. Lung Cancer 2010, 68, 192–197, doi:10.1016/j.lungcan.2009.06.019.

### Appendix A.2. Genomics

- Anderson, M.; Sladon, S.; Michels, R.; Davidson, L.; Conwell, K., 2nd; Lechner, J.; Franklin, W.; Saccomanno, G.; Wiest, J. Examination of p53 alterations and cytokeratin expression in sputa collected from patients prior to histological diagnosis of squamous cell carcinoma. J Cell Biochem Suppl 1996, 25, 185–190.
- Besaratinia, A.; Maas, L.M.; Van Breda, S.G.; Curfs, D.M.; Kleinjans, J.C.; Wouters, E.F.; Van Schooten, F.J. Applicability of induced sputum for molecular dosimetry of exposure to inhalatory carcinogens: 32P-postlabeling of lipophilic DNA adducts in smokers and nonsmokers. Cancer Epidemiol Biomarkers Prev 2000, 9, 367–372.
- Carozzi, F.M.; Bisanzi, S.; Falini, P.; Sani, C.; Venturini, G.; Lopes Pegna, A.; Bianchi, R.; Ronchi, C.; Picozzi, G.; Mascalchi, M.; et al. Molecular profile in body fluids in subjects enrolled in a randomised trial for lung cancer screening: Perspectives of integrated strategies for early diagnosis. Lung Cancer 2010, 68, 216–221, doi:10.1016/j.lungcan.2009.06.015.
- Chen, J.T.; Chen, Y.C.; Wang, Y.C.; Tseng, R.C.; Chen, C.Y.; Wang, Y.C. Alterations of the p16(ink4a) gene in resected nonsmall cell lung tumors and exfoliated cells within sputum. Int J Cancer 2002, 98, 724–731, doi:10.1002/ijc.10262.
- Chen, J.T.; Ho, W.L.; Cheng, Y.W.; Lee, H. Detection of p53 mutations in sputum smears precedes diagnosis of non-small cell lung carcinoma. Anticancer Res 2000, 20, 2687–2690.
- Destro, A.; Bianchi, P.; Alloisio, M.; Laghi, L.; Di Gioia, S.; Malesci, A.; Cariboni, U.; Gribaudi, G.; Bulfamante, G.; Marchetti, A.; et al. K-ras and p16(INK4A)alterations in sputum of NSCLC patients and in heavy asymptomatic chronic smokers. Lung Cancer 2004, 44, 23–32, doi:10.1016/j.lungcan.2003.10.002.
- Emaus, M.N.; Anderson, J.L. Selective extraction of low-abundance BRAF V600E mutation from plasma, urine, and sputum using ion-tagged oligonucleotides and magnetic ionic liquids. Anal Bioanal Chem 2022, 414, 277–286, doi:10.1007/s00216-021-03216-8.
- Feng, Z.; Tian, D.; Lan, Q.; Mumford, J.L. A sensitive immunofluorescence assay for detection of p53 protein accumulation in sputum. Anticancer Res 1999, 19, 3847–3852.
- Guo, X.-j.; Ni, P.-h.; Li, L.; Deng, W.-w.; Wan, H.-y.; Shi, G.-c. Detection of p53 gene mutation of bronchoscopic samples in the patients suspected to lung cancer. Chinese Journal of Cancer Research 2000, 12, 282–285, doi:10.1007/bf02983507.
- Hackner, K.; Buder, A.; Hochmair, M.J.; Strieder, M.; Grech, C.; Fabikan, H.; Burghuber, O.C.; Errhalt, P.; Filipits, M. Detection of EGFR activating and resistance mutations by droplet digital PCR in sputum of EGFR-mutated NSCLC patients. Clin Med Insights Oncol 2021, 15, 1179554921993072, doi:10.1177/1179554921993072.
- Han, B.; Liao, M.; Bao, G.; Feng, J.; Dong, Q. [The relationship between smoking and p53 and Ki-ras gene mutations in sputum cells of patients with lung cancer]. Zhongguo Fei Ai Za Zhi 2001, 4, 41–43, doi:10.3779/j.issn.1009-3419.2001.01.11.
- He, C.; Wei, C.; Wen, J.; Chen, S.; Chen, L.; Wu, Y.; Shen, Y.; Bai, H.; Zhang, Y.; Chen, X.; et al. Comprehensive analysis of NGS and ARMS-PCR for detecting EGFR mutations based on 4467 cases of NSCLC patients. J Cancer Res Clin Oncol 2022, 148, 321–330, doi:10.1007/s00432-021-03818-w.
- Hubers, A.J.; Heideman, D.A.; Yatabe, Y.; Wood, M.D.; Tull, J.; Taron, M.; Molina, M.A.; Mayo, C.; Bertran-Alamillo, J.; Herder, G.J.; et al. EGFR mutation analysis in sputum of lung cancer patients: a multitechnique study. Lung Cancer 2013, 82, 38–43, doi:10.1016/j.lungcan.2013.07.011.
- Isaka, T.; Yokose, T.; Ito, H.; Nakayama, H.; Miyagi, Y.; Saito, H.; Masuda, M. Detection of EGFR mutation of pulmonary adenocarcinoma in sputum using droplet digital PCR. BMC Pulm Med 2021, 21, 100, doi:10.1186/s12890-021-01468-9.
- Jakupciak, J.P.; Maragh, S.; Markowitz, M.E.; Greenberg, A.K.; Hoque, M.O.; Maitra, A.; Barker, P.E.; Wagner, P.D.; Rom, W.N.; Srivastava, S.; et al. Performance of mitochondrial DNA mutations detecting early stage cancer. BMC Cancer 2008, 8, 285, doi:10.1186/1471-2407-8-285.
- Jiang, F.; Todd, N.W.; Li, R.; Zhang, H.; Fang, H.; Stass, S.A. A panel of sputum-based genomic marker for early detection of lung cancer. Cancer Prev Res (Phila) 2010, 3, 1571–1578, doi:10.1158/1940-6207.CAPR-10-0128.
- Jiang, F.; Todd, N.W.; Qiu, Q.; Liu, Z.; Katz, R.L.; Stass, S.A. Combined genetic analysis of sputum and computed tomography for noninvasive diagnosis of non-small-cell lung cancer. Lung Cancer 2009, 66, 58–63, doi:10.1016/j.lungcan.2009.01.004.
- Keohavong, P.; Gao, W.M.; Zheng, K.C.; Mady, H.; Lan, Q.; Melhem, M.; Mumford, J. Detection of K-ras and p53 mutations in sputum samples of lung cancer patients using laser capture microdissection microscope and mutation analysis. Anal Biochem 2004, 324, 92–99, doi:10.1016/j.ab.2003.09.030.
- Keohavong, P.; Lan, Q.; Gao, W.M.; DeMarini, D.M.; Mass, M.J.; Li, X.M.; Roop, B.C.; Weissfeld, J.; Tian, D.; Mumford, J.L. K-ras mutations in lung carcinomas from nonsmoking women exposed to unvented coal smoke in China. Lung Cancer 2003, 41, 21–27, doi:10.1016/s0169-5002(03)00125-9.
- Keohavong, P.; Lan, Q.; Gao, W.M.; Zheng, K.C.; Mady, H.H.; Melhem, M.F.; Mumford, J.L. Detection of p53 and K-ras mutations in sputum of individuals exposed to smoky coal emissions in Xuan Wei County, China. Carcinogenesis 2005, 26, 303–308, doi:10.1093/carcin/bgh328.
- Kim, I.A.; Hur, J.Y.; Kim, H.J.; Kim, W.S.; Lee, K.Y. Extracellular vesicle-based bronchoalveolar lavage fluid liquid biopsy for EGFR mutation testing in advanced non-squamous NSCLC. Cancers (Basel) 2022, 14, doi:10.3390/cancers14112744.
- Lan, Q.; Feng, Z.; Tian, D.; He, X.; Rothman, N.; Tian, L.; Lu, X.; Terry, M.B.; Mumford, J.L. p53 gene expression in relation to indoor exposure to unvented coal smoke in Xuan Wei, China. J Occup Environ Med 2001, 43, 226–230, doi:10.1097/00043764-200103000-00010.
- Li, R.; Todd, N.W.; Qiu, Q.; Fan, T.; Zhao, R.Y.; Rodgers, W.H.; Fang, H.B.; Katz, R.L.; Stass, S.A.; Jiang, F. Genetic deletions in sputum as diagnostic markers for early detection of stage I non-small cell lung cancer. Clin Cancer Res 2007, 13, 482–487, doi:10.1158/1078-0432.CCR-06-1593.
- Mao, L.; Hruban, R.H.; Boyle, J.O.; Tockman, M.; Sidransky, D. Detection of oncogene mutations in sputum precedes diagnosis of lung cancer. Cancer Res 1994, 54, 1634–1637.
- Marchetti, A.; Buttitta, F.; Carnicelli, V.; Pellegrini, S.; Bertacca, G.; Merlo, G.; Bevilacqua, G. Enriched SSCP: a highly sensitive method for the detection of unknown mutations. Application to the molecular diagnosis of lung cancer in sputum samples. Diagn Mol Pathol 1997, 6, 185–191, doi:10.1097/00019606-199708000-00002.
- Morikawa, K.; Kinoshita, K.; Kida, H.; Inoue, T.; Mineshita, M. Preliminary results of NGS gene panel test using NSCLC sputum cytology and therapeutic effect using corresponding molecular-targeted drugs. Genes (Basel) 2022, 13, doi:10.3390/genes13050812.
- Morikawa, K.; Kinoshita, K.; Matsuzawa, S.; Kida, H.; Handa, H.; Inoue, T.; Nakamura, S.; Sato, Y.; Mineshita, M. EML4-ALK gene mutation detected with new NGS lung cancer panel CDx using sputum cytology in a case of advanced NSCLC. Diagnostics (Basel) 2023, 13, 2327, doi:10.3390/diagnostics13142327.
- Nakajima, E.; Hirano, T.; Konaka, C.; Ikeda, N.; Kawate, N.; Ebihara, Y.; Kato, H. K-ras mutation in sputum of primary lung cancer patients does not always reflect that of cancerous cells. Int J Oncol 2001, doi:10.3892/ijo.18.1.105.
- Nishikawa, T.; Fujii, T.; Tatsumi, S.; Sugimoto, A.; Sekita-Hatakeyama, Y.; Shimada, K.; Yamazaki, M.; Hatakeyama, K.; Ohbayashi, C. Molecular analysis of liquid-based cytological specimen using virtually positive sputum with adenocarcinoma cells. Diagnostics (Basel) 2020, 10, doi:10.3390/diagnostics10020084.
- Oh, S.Y.; Lee, H.T. Efficiency of EGFR mutation analysis for small microdissected cytological specimens using multitech DNA extraction solution. Cancer Cytopathol 2015, 123, 401–412, doi:10.1002/cncy.21550.
- Papadakis, E.D.; Soulitzis, N.; Spandidos, D.A. Association of p53 codon 72 polymorphism with advanced lung cancer: the Arg allele is preferentially retained in tumours arising in Arg/Pro germline heterozygotes. Br J Cancer 2002, 87, 1013–1018, doi:10.1038/sj.bjc.6600595.
- Qin, L.; Guo, T.; Yang, H.; Deng, P.; Gu, Q.; Liu, C.; Wu, M.; Lizaso, A.; Li, B.; Zhang, S.; et al. The utility of sputum supernatant as an alternative liquid biopsy specimen for next-generation sequencing-based somatic variation profiling. Ann Transl Med 2022, 10, 462, doi:10.21037/atm-22-1297.
- Ronai, Z.; Yabubovskaya, M.S.; Zhang, E.; Belitsky, G.A. K-ras mutation in sputum of patients with or without lung cancer. J Cell Biochem Suppl 1996, 25, 172–176.
- Soda, M.; Isobe, K.; Inoue, A.; Maemondo, M.; Oizumi, S.; Fujita, Y.; Gemma, A.; Yamashita, Y.; Ueno, T.; Takeuchi, K.; et al. A prospective PCR-based screening for the EML4-ALK oncogene in non-small cell lung cancer. Clin Cancer Res 2012, 18, 5682–5689, doi:10.1158/1078-0432.CCR-11-2947.
- Somers, V.A.; Pietersen, A.M.; Theunissen, P.H.; Thunnissen, F.B. Detection of K-ras point mutations in sputum from patients with adenocarcinoma of the lung by point-EXACCT. J Clin Oncol 1998, 16, 3061–3068, doi:10.1200/JCO.1998.16.9.3061.
- Su, F.; Fu, Y.; Wu, Q.; Zheng, K.; Tang, Y.; Su, X.; Wang, Y.; Jiang, L. High concordance of EGFR mutation status between sputum and corresponding tissue specimens of late-stage lung cancers using amplification refractory mutation system-PCR. Int J Clin Exp Pathol 2018, 11, 2683–2690.
- Takeda, S.; Ichii, S.; Nakamura, Y. Detection of K-ras mutation in sputum by mutant-allele-specific amplification (MASA). Hum Mutat 1993, 2, 112–117, doi:10.1002/humu.1380020209.
- Tanaka, T.; Nagai, Y.; Miyazawa, H.; Koyama, N.; Matsuoka, S.; Sutani, A.; Huqun; Udagawa, K.; Murayama, Y.; Nagata, M.; et al. Reliability of the peptide nucleic acid-locked nucleic acid polymerase chain reaction clamp-based test for epidermal growth factor receptor mutations integrated into the clinical practice for non-small cell lung cancers. Cancer Sci 2007, 98, 246–252, doi:10.1111/j.1349-7006.2006.00377.x.
- Wang, B.; Li, L.; Yao, L.; Liu, L.; Zhu, Y. [Detection of p53 gene alteration in sputum sample and its implications in early diagnosis of lung cancer]. Zhonghua Nei Ke Za Zhi 2001, 40, 101–104.
- Wang, L.; Wang, G.; Xia, X. [Detection of oncogenic mutations in sputum and its diagnostic value of lung cancer]. Zhonghua Jie He He Hu Xi Za Zhi 1998, 21, 236–239.
- Wang, Y.; Liu, Y.; Zhao, C.; Li, X.; Wu, C.; Hou, L.; Zhang, S.; Jiang, T.; Chen, X.; Su, C.; et al. Feasibility of cytological specimens for ALK fusion detection in patients with advanced NSCLC using the method of RT-PCR. Lung Cancer 2016, 94, 28–34, doi:10.1016/j.lungcan.2016.01.014.
- Wang, Z.; Li, L.; Wang, Y.; Li, X.; Xu, Y.; Wang, M.; Liang, L.; Wu, X.; Tang, M.; Li, Y.; et al. Sputum cell-free DNA for detection of alterations of multiple driver genes in lung adenocarcinoma. Cancer Cytopathol 2023, 131, 110–116, doi:10.1002/cncy.22644.
- Wang, Z.; Li, X.; Zhang, L.; Xu, Y.; Wang, M.; Liang, L.; Jiao, P.; Li, Y.; He, S.; Du, J.; et al. Sputum cell-free DNA: Valued surrogate sample for the detection of EGFR exon 20 p.T790M mutation in patients with advanced lung adenocarcinoma and acquired resistance to EGFR-TKIs. Cancer Med 2021, 10, 3323–3331, doi:10.1002/cam4.3817.
- Wang, Z.; Zhang, L.; Li, L.; Li, X.; Xu, Y.; Wang, M.; Liang, L.; Jiao, P.; Li, Y.; He, S.; et al. Sputum cell-free DNA: Valued surrogate sample for detection of EGFR mutation in patients with advanced lung adenocarcinoma. J Mol Diagn 2020, 22, 934–942, doi:10.1016/j.jmoldx.2020.04.208.
- Wu, Z.; Yang, Z.; Li, C.S.; Zhao, W.; Liang, Z.X.; Dai, Y.; Zhu, Q.; Miao, K.L.; Cui, D.H.; Chen, L.A. Differences in the genomic profiles of cell-free DNA between plasma, sputum, urine, and tumor tissue in advanced NSCLC. Cancer Med 2019, 8, 910–919, doi:10.1002/cam4.1935.
- Wu, Z.; Yang, Z.; Li, C.S.; Zhao, W.; Liang, Z.X.; Dai, Y.; Zeng, J.; Zhu, Q.; Miao, K.L.; Cui, D.H.; et al. Non-invasive detection of EGFR and TP53 mutations through the combination of plasma, urine and sputum in advanced non-small cell lung cancer. Oncol Lett 2019, 18, 3581–3590, doi:10.3892/ol.2019.10726.
- Xie, X.; Wu, J.; Guo, B.; Wang, L.; Deng, H.; Lin, X.; Liu, M.; Qin, Y.; Luo, W.; Yang, Y.; et al. Comprehensive characterization reveals sputum supernatant as a valuable alternative liquid biopsy for genome profiling in advanced non-small cell lung cancer. Respir Res 2022, 23, 175, doi:10.1186/s12931-022-02097-4.
- Yakubovskaya, M.S.; Spiegelman, V.; Luo, F.C.; Malaev, S.; Salnev, A.; Zborovskaya, I.; Gasparyan, A.; Polotsky, B.; Machaladze, Z.; Trachtenberg, A.C.; et al. High frequency of K-ras mutations in normal appearing lung tissues and sputum of patients with lung cancer. Int J Cancer 1995, 63, 810–814, doi:10.1002/ijc.2910630611.
- Zhang, L.F.; Gao, W.M.; Gealy, R.; Weissfeld, J.; Elder, E.; Whiteside, T.L.; Keohavong, P. Comparison of K-ras gene mutations in tumour and sputum DNA of patients with lung cancer. Biomarkers 2003, 8, 156–161, doi:10.1080/1354750021000046589.

### Appendix A.3. Epigenetics

- Belinsky, S.A.; Grimes, M.J.; Casas, E.; Stidley, C.A.; Franklin, W.A.; Bocklage, T.J.; Johnson, D.H.; Schiller, J.H. Predicting gene promoter methylation in non-small-cell lung cancer by evaluating sputum and serum. Br J Cancer 2007, 96, 1278–1283, doi:10.1038/sj.bjc.6603721.
- Belinsky, S.A.; Klinge, D.M.; Dekker, J.D.; Smith, M.W.; Bocklage, T.J.; Gilliland, F.D.; Crowell, R.E.; Karp, D.D.; Stidley, C.A.; Picchi, M.A. Gene promoter methylation in plasma and sputum increases with lung cancer risk. Clin Cancer Res 2005, 11, 6505–6511, doi:10.1158/1078-0432.CCR-05-0625.
- Belinsky, S.A.; Leng, S.; Wu, G.; Thomas, C.L.; Picchi, M.A.; Lee, S.J.; Aisner, S.; Ramalingam, S.; Khuri, F.R.; Karp, D.D. Gene methylation biomarkers in sputum and plasma as predictors for lung cancer recurrence. Cancer Prev Res (Phila) 2017, 10, 635–640, doi:10.1158/1940-6207.CAPR-17-0177.
- Belinsky, S.A.; Liechty, K.C.; Gentry, F.D.; Wolf, H.J.; Rogers, J.; Vu, K.; Haney, J.; Kennedy, T.C.; Hirsch, F.R.; Miller, Y.; et al. Promoter hypermethylation of multiple genes in sputum precedes lung cancer incidence in a high-risk cohort. Cancer Res 2006, 66, 3338–3344, doi:10.1158/0008-5472.CAN-05-3408.
- Belinsky, S.A.; Nikula, K.J.; Palmisano, W.A.; Michels, R.; Saccomanno, G.; Gabrielson, E.; Baylin, S.B.; Herman, J.G. Aberrant methylation of p16(INK4a) is an early event in lung cancer and a potential biomarker for early diagnosis. Proc Natl Acad Sci U S A 1998, 95, 11891–11896, doi:10.1073/pnas.95.20.11891.
- Belinsky, S.A.; Palmisano, W.A.; Gilliland, F.D.; Crooks, L.A.; Divine, K.K.; Winters, S.A.; Grimes, M.J.; Harms, H.J.; Tellez, C.S.; Smith, T.M.; et al. Aberrant promoter methylation in bronchial epithelium and sputum from current and former smokers. Cancer Res 2002, 62, 2370–2377.
- Bruse, S.; Petersen, H.; Weissfeld, J.; Picchi, M.; Willink, R.; Do, K.; Siegfried, J.; Belinsky, S.A.; Tesfaigzi, Y. Increased methylation of lung cancer-associated genes in sputum DNA of former smokers with chronic mucous hypersecretion. Respir Res 2014, 15, 2, doi:10.1186/1465-9921-15-2.
- Cirincione, R.; Lintas, C.; Conte, D.; Mariani, L.; Roz, L.; Vignola, A.M.; Pastorino, U.; Sozzi, G. Methylation profile in tumor and sputum samples of lung cancer patients detected by spiral computed tomography: a nested case-control study. Int J Cancer 2006, 118, 1248–1253, doi:10.1002/ijc.21473.
- Diaz-Lagares, A.; Mendez-Gonzalez, J.; Hervas, D.; Saigi, M.; Pajares, M.J.; Garcia, D.; Crujerias, A.B.; Pio, R.; Montuenga, L.M.; Zulueta, J.; et al. A novel epigenetic signature for early diagnosis in lung cancer. Clin Cancer Res 2016, 22, 3361–3371, doi:10.1158/1078-0432.CCR-15-2346.
- Flores, K.G.; Stidley, C.A.; Mackey, A.J.; Picchi, M.A.; Stabler, S.P.; Siegfried, J.M.; Byers, T.; Berwick, M.; Belinsky, S.A.; Leng, S. Sex-specific association of sequence variants in CBS and MTRR with risk for promoter hypermethylation in the lung epithelium of smokers. Carcinogenesis 2012, 33, 1542–1547, doi:10.1093/carcin/bgs194.
- Fumagalli, C.; Bianchi, F.; Raviele, P.R.; Vacirca, D.; Bertalot, G.; Rampinelli, C.; Lazzeroni, M.; Bonanni, B.; Veronesi, G.; Fusco, N.; et al. Circulating and tissue biomarkers in early-stage non-small cell lung cancer. Ecancermedicalscience 2017, 11, 717, doi:10.3332/ecancer.2017.717.
- Gaballah, I.F.; Helal, S.F.; Mourad, B.H. Early detection of lung cancer potential among Egyptian wood workers. Int J Occup Environ Health 2017, 23, 120–127, doi:10.1080/10773525.2018.1428265.
- Georgiou, E.; Valeri, R.; Tzimagiorgis, G.; Anzel, J.; Krikelis, D.; Tsilikas, C.; Sarikos, G.; Destouni, C.; Dimitriadou, A.; Kouidou, S. Aberrant p16 promoter methylation among Greek lung cancer patients and smokers: correlation with smoking. Eur J Cancer Prev 2007, 16, 396–402, doi:10.1097/01.cej.0000236260.26265.d6.
- Gilliland, F.D.; Harms, H.J.; Crowell, R.E.; Li, Y.F.; Willink, R.; Belinsky, S.A. Glutathione S-transferase P1 and NADPH quinone oxidoreductase polymorphisms are associated with aberrant promoter methylation of P16(INK4a) and O(6)-methylguanine-DNA methyltransferase in sputum. Cancer Res 2002, 62, 2248–2252.
- Guzman, L.; Depix, M.S.; Salinas, A.M.; Roldan, R.; Aguayo, F.; Silva, A.; Vinet, R. Analysis of aberrant methylation on promoter sequences of tumor suppressor genes and total DNA in sputum samples: a promising tool for early detection of COPD and lung cancer in smokers. Diagn Pathol 2012, 7, 87, doi:10.1186/1746-1596-7-87.
- Hubers, A.J.; Brinkman, P.; Boksem, R.J.; Rhodius, R.J.; Witte, B.I.; Zwinderman, A.H.; Heideman, D.A.; Duin, S.; Koning, R.; Steenbergen, R.D.; et al. Combined sputum hypermethylation and eNose analysis for lung cancer diagnosis. J Clin Pathol 2014, 67, 707–711, doi:10.1136/jclinpath-2014-202414.
- Hubers, A.J.; Heideman, D.A.; Burgers, S.A.; Herder, G.J.; Sterk, P.J.; Rhodius, R.J.; Smit, H.J.; Krouwels, F.; Welling, A.; Witte, B.I.; et al. DNA hypermethylation analysis in sputum for the diagnosis of lung cancer: training validation set approach. Br J Cancer 2015, 112, 1105–1113, doi:10.1038/bjc.2014.636.
- Hubers, A.J.; Heideman, D.A.; Duin, S.; Witte, B.I.; de Koning, H.J.; Groen, H.J.; Prinsen, C.F.; Bolijn, A.S.; Wouters, M.; van der Meer, S.E.; et al. DNA hypermethylation analysis in sputum of asymptomatic subjects at risk for lung cancer participating in the NELSON trial: argument for maximum screening interval of 2 years. J Clin Pathol 2017, 70, 250–254, doi:10.1136/jclinpath-2016-203734.
- Hubers, A.J.; Heideman, D.A.; Herder, G.J.; Burgers, S.A.; Sterk, P.J.; Kunst, P.W.; Smit, H.J.; Postmus, P.E.; Witte, B.I.; Duin, S.; et al. Prolonged sampling of spontaneous sputum improves sensitivity of hypermethylation analysis for lung cancer. J Clin Pathol 2012, 65, 541–545, doi:10.1136/jclinpath-2012-200712.
- Hubers, A.J.; van der Drift, M.A.; Prinsen, C.F.; Witte, B.I.; Wang, Y.; Shivapurkar, N.; Stastny, V.; Bolijn, A.S.; Hol, B.E.; Feng, Z.; et al. Methylation analysis in spontaneous sputum for lung cancer diagnosis. Lung Cancer 2014, 84, 127–133, doi:10.1016/j.lungcan.2014.01.019.
- Hulbert, A.; Jusue-Torres, I.; Stark, A.; Chen, C.; Rodgers, K.; Lee, B.; Griffin, C.; Yang, A.; Huang, P.; Wrangle, J.; et al. Early detection of lung cancer using DNA promoter hypermethylation in plasma and sputum. Clin Cancer Res 2017, 23, 1998–2005, doi:10.1158/1078-0432.CCR-16-1371.
- Hwang, S.H.; Kim, K.U.; Kim, J.E.; Kim, H.H.; Lee, M.K.; Lee, C.H.; Lee, S.Y.; Oh, T.; An, S. Detection of HOXA9 gene methylation in tumor tissues and induced sputum samples from primary lung cancer patients. Clin Chem Lab Med 2011, 49, 699–704, doi:10.1515/CCLM.2011.108.
- Kim, J.P.; Kim, K.M.; Kwon, S.S.; Kim, Y.K.; Kim, K.H.; Moon, H.S.; Song, J.S.; Park, S.H.; Ahn, J.H. p16INK4a Promoter hypermethylation in sputum, blood, and tissue from non-small cell lung cancer and pulmonary inflammation. Tuberc Respir Dis (Seoul) 2006, 60, 160–171, doi:10.4046/trd.2006.60.02.160.
- Konno, S.; Morishita, Y.; Fukasawa, M.; Shu, Y.; Wang, D.; Tanaka, R.; Minami, Y.; Iijima, T.; Noguchi, M. Anthracotic index and DNA methylation status of sputum contents can be used for identifying the population at risk of lung carcinoma. Cancer 2004, 102, 348–354, doi:10.1002/cncr.20643.
- Leng, S.; Bernauer, A.M.; Hong, C.; Do, K.C.; Yingling, C.M.; Flores, K.G.; Tessema, M.; Tellez, C.S.; Willink, R.P.; Burki, E.A.; et al. The A/G allele of rs16906252 predicts for MGMT methylation and is selectively silenced in premalignant lesions from smokers and in lung adenocarcinomas. Clin Cancer Res 2011, 17, 2014–2023, doi:10.1158/1078-0432.CCR-10-3026.
- Leng, S.; Do, K.; Yingling, C.M.; Picchi, M.A.; Wolf, H.J.; Kennedy, T.C.; Feser, W.J.; Baron, A.E.; Franklin, W.A.; Brock, M.V.; et al. Defining a gene promoter methylation signature in sputum for lung cancer risk assessment. Clin Cancer Res 2012, 18, 3387–3395, doi:10.1158/1078-0432.CCR-11-3049.
- Leng, S.; Liu, Y.; Thomas, C.L.; Gauderman, W.J.; Picchi, M.A.; Bruse, S.E.; Zhang, X.; Flores, K.G.; Van Den Berg, D.; Stidley, C.A.; et al. Native American ancestry affects the risk for gene methylation in the lungs of Hispanic smokers from New Mexico. Am J Respir Crit Care Med 2013, 188, 1110–1116, doi:10.1164/rccm.201305-0925OC.
- Leng, S.; Liu, Y.; Weissfeld, J.L.; Thomas, C.L.; Han, Y.; Picchi, M.A.; Edlund, C.K.; Willink, R.P.; Gaither Davis, A.L.; Do, K.C.; et al. 15q12 variants, sputum gene promoter hypermethylation, and lung cancer risk: a GWAS in smokers. J Natl Cancer Inst 2015, 107, doi:10.1093/jnci/djv035.
- Leng, S.; Picchi, M.A.; Kang, H.; Wu, G.; Filipczak, P.T.; Juri, D.E.; Zhang, X.; Gauderman, W.J.; Gilliland, F.D.; Belinsky, S.A. Dietary nutrient intake, ethnicity, and epigenetic silencing of lung cancer genes detected in sputum in New Mexican smokers. Cancer Prev Res (Phila) 2018, 11, 93–102, doi:10.1158/1940-6207.CAPR-17-0196.
- Leng, S.; Stidley, C.A.; Liu, Y.; Edlund, C.K.; Willink, R.P.; Han, Y.; Landi, M.T.; Thun, M.; Picchi, M.A.; Bruse, S.E.; et al. Genetic determinants for promoter hypermethylation in the lungs of smokers: a candidate gene-based study. Cancer Res 2012, 72, 707–715, doi:10.1158/0008-5472.CAN-11-3194.
- Leng, S.; Stidley, C.A.; Willink, R.; Bernauer, A.; Do, K.; Picchi, M.A.; Sheng, X.; Frasco, M.A.; Van Den Berg, D.; Gilliland, F.D.; et al. Double-strand break damage and associated DNA repair genes predispose smokers to gene methylation. Cancer Res 2008, 68, 3049–3056, doi:10.1158/0008-5472.CAN-07-6344.
- Leng, S.; Wu, G.; Collins, L.B.; Thomas, C.L.; Tellez, C.S.; Jauregui, A.R.; Picchi, M.A.; Zhang, X.; Juri, D.E.; Desai, D.; et al. Implication of a chromosome 15q15.2 locus in regulating UBR1 and predisposing smokers to MGMT methylation in lung. Cancer Res 2015, 75, 3108–3117, doi:10.1158/0008-5472.CAN-15-0243.
- Leng, S.; Wu, G.; Klinge, D.M.; Thomas, C.L.; Casas, E.; Picchi, M.A.; Stidley, C.A.; Lee, S.J.; Aisner, S.; Siegfried, J.M.; et al. Gene methylation biomarkers in sputum as a classifier for lung cancer risk. Oncotarget 2017, 8, 63978–63985, doi:10.18632/oncotarget.19255.
- Liu, J.Y.; An, Q.; Xu, G.D.; Lei, W.D.; Li, L.; Pan, Q.J.; Han, N.J.; Cheng, S.J.; Gao, Y.N. [Hypermethylation of p16 gene in clinical specimens of patients with lung cancer]. Zhonghua Zhong Liu Za Zhi 2004, 26, 75–77.
- Liu, W.B.; Han, F.; Huang, Y.S.; Chen, H.Q.; Chen, J.P.; Wang, D.D.; Jiang, X.; Yin, L.; Cao, J.; Liu, J.Y. TMEM196 hypermethylation as a novel diagnostic and prognostic biomarker for lung cancer. Mol Carcinog 2019, 58, 474–487, doi:10.1002/mc.22942.
- Liu, W.B.; Han, F.; Jiang, X.; Yin, L.; Chen, H.Q.; Li, Y.H.; Liu, Y.; Cao, J.; Liu, J.Y. Epigenetic regulation of ANKRD18B in lung cancer. Mol Carcinog 2015, 54, 312–321, doi:10.1002/mc.22101.
- Liu, Y.; An, Q.; Li, L.; Zhang, D.; Huang, J.; Feng, X.; Cheng, S.; Gao, Y. Hypermethylation of p16INK4a in Chinese lung cancer patients: biological and clinical implications. Carcinogenesis 2003, 24, 1897–1901, doi:10.1093/carcin/bgg169.
- Liu, Y.; Lan, Q.; Shen, M.; Jin, J.; Mumford, J.; Ren, D.; Keohavong, P. Aberrant gene promoter methylation in sputum from individuals exposed to smoky coal emissions. Anticancer Res 2008, 28, 2061–2066.
- Machida, E.O.; Brock, M.V.; Hooker, C.M.; Nakayama, J.; Ishida, A.; Amano, J.; Picchi, M.A.; Belinsky, S.A.; Herman, J.G.; Taniguchi, S.; et al. Hypermethylation of ASC/TMS1 is a sputum marker for late-stage lung cancer. Cancer Res 2006, 66, 6210–6218, doi:10.1158/0008-5472.CAN-05-4447.
- Millares, L.; Rosell, A.; Seto, L.; Sanz, J.; Andreo, F.; Monso, E. Variability in the measurement of the methylation status of lung cancer-related genes in bronchial secretions. Oncol Rep 2014, 32, 1435–1440, doi:10.3892/or.2014.3364.
- Mohammed, F.; Baydaa Abed Hussein, A.; Ahmed, T. Evaluation of methylation panel in the promoter region of p16(INK4a), RASSF1A, and MGMT as a biomarker in sputum for lung cancer. Arch Razi Inst 2022, 77, 1075–1081, doi:10.22092/ARI.2022.357985.2131.
- Olaussen, K.A.; Soria, J.C.; Park, Y.W.; Kim, H.J.; Kim, S.H.; Ro, J.Y.; Andre, F.; Jang, S.J. Assessing abnormal gene promoter methylation in paraffin-embedded sputum from patients with NSCLC. Eur J Cancer 2005, 41, 2112–2119, doi:10.1016/j.ejca.2005.06.013.
- Palmisano, W.A.; Divine, K.K.; Saccomanno, G.; Gilliland, F.D.; Baylin, S.B.; Herman, J.G.; Belinsky, S.A. Predicting lung cancer by detecting aberrant promoter methylation in sputum. Cancer Res 2000, 60, 5954–5958.
- Peng, Z.; Shan, C.; Wang, H. [Value of promoter methylation of RASSF1A, p16, and DAPK genes in induced sputum in diagnosing lung cancers]. Zhong Nan Da Xue Xue Bao Yi Xue Ban 2010, 35, 247–253, doi:10.3969/j.issn.1672-7347.2010.03.010.
- Rosell, A.; Rodriguez, N.; Monso, E.; Taron, M.; Millares, L.; Ramirez, J.L.; Lopez-Lisbona, R.; Cubero, N.; Andreo, F.; Sanz, J.; et al. Aberrant gene methylation and bronchial dysplasia in high risk lung cancer patients. Lung Cancer 2016, 94, 102–107, doi:10.1016/j.lungcan.2016.02.003.
- Shivapurkar, N.; Stastny, V.; Suzuki, M.; Wistuba, II; Li, L.; Zheng, Y.; Feng, Z.; Hol, B.; Prinsen, C.; Thunnissen, F.B.; et al. Application of a methylation gene panel by quantitative PCR for lung cancers. Cancer Lett 2007, 247, 56–71, doi:10.1016/j.canlet.2006.03.020.
- Shivapurkar, N.; Stastny, V.; Xie, Y.; Prinsen, C.; Frenkel, E.; Czerniak, B.; Thunnissen, F.B.; Minna, J.D.; Gazdar, A.F. Differential methylation of a short CpG-rich sequence within exon 1 of TCF21 gene: a promising cancer biomarker assay. Cancer Epidemiol Biomarkers Prev 2008, 17, 995–1000, doi:10.1158/1055-9965.EPI-07-2808.
- Soukiasian, H.J.; Leung, A.; Imai, T.; Bose, S.; Kim, S.; Mosenifar, Z.; Gupta, N.K.; Tajbakhsh, J. Highly sensitive noninvasive early lung cancer detection using DNA methylation topology in sputum-derived epithelial cells. JTCVS Open 2023, 13, 389–410, doi:10.1016/j.xjon.2022.11.018.
- Su, S.B.; Yang, L.J.; Zhang, W.; Jin, Y.L.; Nie, J.H.; Tong, J. [p16 and MGMT gene methylation in sputum cells of uranium workers]. Zhonghua Lao Dong Wei Sheng Zhi Ye Bing Za Zhi 2006, 24, 92–95.
- Su, Y.; Fang, H.B.; Jiang, F. An epigenetic classifier for early stage lung cancer. Clin Epigenetics 2018, 10, 68, doi:10.1186/s13148-018-0502-3.
- Tajbakhsh, J.; Mortazavi, F.; Gupta, N.K. DNA methylation topology differentiates between normal and malignant in cell models, resected human tissues, and exfoliated sputum cells of lung epithelium. Front Oncol 2022, 12, 991120, doi:10.3389/fonc.2022.991120.
- Tessema, M.; Klinge, D.M.; Yingling, C.M.; Do, K.; Van Neste, L.; Belinsky, S.A. Re-expression of CXCL14, a common target for epigenetic silencing in lung cancer, induces tumor necrosis. Oncogene 2010, 29, 5159–5170, doi:10.1038/onc.2010.255.
- Tessema, M.; Tassew, D.D.; Yingling, C.M.; Do, K.; Picchi, M.A.; Wu, G.; Petersen, H.; Randell, S.; Lin, Y.; Belinsky, S.A.; et al. Identification of novel epigenetic abnormalities as sputum biomarkers for lung cancer risk among smokers and COPD patients. Lung Cancer 2020, 146, 189–196, doi:10.1016/j.lungcan.2020.05.017.
- Tessema, M.; Yingling, C.M.; Picchi, M.A.; Wu, G.; Liu, Y.; Weissfeld, J.L.; Siegfried, J.M.; Tesfaigzi, Y.; Belinsky, S.A. Epigenetic repression of CCDC37 and MAP1B links chronic obstructive pulmonary disease to lung cancer. J Thorac Oncol 2015, 10, 1181–1188, doi:10.1097/JTO.0000000000000592.
- Wang, Y.C.; Lu, Y.P.; Tseng, R.C.; Lin, R.K.; Chang, J.W.; Chen, J.T.; Shih, C.M.; Chen, C.Y. Inactivation of hMLH1 and hMSH2 by promoter methylation in primary non-small cell lung tumors and matched sputum samples. J Clin Invest 2003, 111, 887–895, doi:10.1172/JCI15475.
- Zhang, W.; Sun, Y.; Lu, G. [The diagnostic value of determination of p16 methylation of sputum exfoliated cells for peripheral lung cancer]. Zhongguo Fei Ai Za Zhi 2004, 7, 46–49, doi:10.3779/j.issn.1009-3419.2004.01.12.
- Zochbauer-Muller, S.; Lam, S.; Toyooka, S.; Virmani, A.K.; Toyooka, K.O.; Seidl, S.; Minna, J.D.; Gazdar, A.F. Aberrant methylation of multiple genes in the upper aerodigestive tract epithelium of heavy smokers. Int J Cancer 2003, 107, 612–616, doi:10.1002/ijc.11458.

### Appendix A.4. Transcriptomics

- Bagheri, A.; Khorram Khorshid, H.R.; Mowla, S.J.; Mohebbi, H.A.; Mohammadian, A.; Yaseri, M.; Solaymani-Dodaran, M.; Sherafatian, M.; Tavallaie, M. Altered miR-223 expression in sputum for diagnosis of non-small cell lung cancer. Avicenna J Med Biotechnol 2017, 9, 189–195.
- Bagheri, A.; Khorshid, H.R.K.; Tavallaie, M.; Mowla, S.J.; Sherafatian, M.; Rashidi, M.; Zargari, M.; Boroujeni, M.E.; Hosseini, S.M. A panel of noncoding RNAs in non-small-cell lung cancer. J Cell Biochem 2019, 120, 8280–8290, doi:10.1002/jcb.28111.
- Bai, C.; Wang, C.; Hua, J.; Zhao, N.; Li, T.; Li, W.; Niu, W.; Zhong, B.; Yang, S.; Chen, C.; et al. Circ_0006949 as a potential non-invasive diagnosis biomarker promotes the proliferation of NSCLC cells via miR-4673/GLUL axis. Biochim Biophys Acta Mol Basis Dis 2024, 1870, doi:10.1016/j.bbadis.2024.167234.
- Chen, E.; Bao, Z.; Zhen, H.; Chen, Y.; Wu, C.; Zhang, J.; Xu, H.; Ding, Y.; Wang, Y.; Yu, F.; et al. Template-ready PCR method for detection of human telomerase reverse transcriptase mRNA in sputum. Anal Biochem 2019, 577, 34–41, doi:10.1016/j.ab.2019.04.008.
- Chen, Y.-q.; Li, D.-m.; Cai, Y.-y.; Liu, C.; Xia, X.-m.; Hu, J.-f. [The expression of survivin messenger RNA in sputum and cancerous tissue in human lung cancer]. Zhonghua Jie He He Hu Xi Za Zhi 2005, 28, 225–229.
- Dong, D.-q.; Yang, Y.-h.; Xue, D.-y.; Feng, X.-j. [Expression of survivin mRNA of sputum and pleural effusions in human lung cancer]. Zhong Nan Da Xue Xue Bao Yi Xue Ban 2006, 31, 848–852.
- Gupta, C.; Su, J.; Zhan, M.; Stass, S.A.; Jiang, F. Sputum long non-coding RNA biomarkers for diagnosis of lung cancer. Cancer Biomark 2019, 26, 219–227, doi:10.3233/cbm-190161.
- Kim, J.O.; Gazala, S.; Razzak, R.; Guo, L.; Ghosh, S.; Roa, W.H.; Bedard, E.L. Non-small cell lung cancer detection using microRNA expression profiling of bronchoalveolar lavage fluid and sputum. Anticancer Res 2015, 35, 1873–1880.
- Lacroix, J.; Becker, H.D.; Woerner, S.M.; Rittgen, W.; Drings, P.; von Knebel Doeberitz, M. Sensitive detection of rare cancer cells in sputum and peripheral blood samples of patients with lung cancer by preproGRP-specific RT-PCR. Int J Cancer 2001, 92, 1–8, doi:10.1002/1097-0215(200102)9999:9999<::Aid-ijc1159>3.0.Co;2-5.
- Lee, H.Y.; Kim, J.I.; Cho, S.H.; Ko, T.Y.; Kim, H.S.; Park, S.D.; Cho, S.R.; Chang, H.K.; Hwang, G.J.; Jung, S.B. Expression of the Brother of the Regulator of Imprinted Sites gene in the sputum of patients with lung cancer. Korean J Thorac Cardiovasc Surg 2014, 47, 378–383, doi:10.5090/kjtcs.2014.47.4.378.
- Li, N.; Ma, J.; Guarnera, M.A.; Fang, H.; Cai, L.; Jiang, F. Digital PCR quantification of miRNAs in sputum for diagnosis of lung cancer. J Cancer Res Clin Oncol 2013, 140, 145–150, doi:10.1007/s00432-013-1555-5.
- Liao, J.; Shen, J.; Leng, Q.; Qin, M.; Zhan, M.; Jiang, F. MicroRNA-based biomarkers for diagnosis of non-small cell lung cancer (NSCLC). Thorac Cancer 2020, 11, 762–768, doi:10.1111/1759-7714.13337.
- Lin, Y.; Holden, V.; Dhilipkannah, P.; Deepak, J.; Todd, N.W.; Jiang, F. A non-coding RNA landscape of bronchial epitheliums of lung cancer patients. Biomedicines 2020, 8, doi:10.3390/biomedicines8040088.
- Pottelberge, G.R.V.; Mestdagh, P.; Bracke, K.R.; Thas, O.; Durme, Y.M.T.A.v.; Joos, G.F.; Vandesompele, J.; Brusselle, G.G. MicroRNA expression in induced sputum of smokers and patients with chronic obstructive pulmonary disease. Am J Respir Crit Care Med 2011, 183, 898–906, doi:10.1164/rccm.201002-0304OC.
- Quin, C.; McClelland, A.; Zeng, T.H. Nano detection of miR-155 for early lung cancer diagnosis via surface-enhanced Raman spectroscopy. In: Proceedings of the 2024 IEEE 24th International Conference on Nanotechnology (NANO), 2024; pp. 311–316.
- Razzak, R.; Bédard, E.L.R.; Kim, J.O.; Gazala, S.; Guo, L.; Ghosh, S.; Joy, A.; Nijjar, T.; Wong, E.; Roa, W.H. MicroRNA expression profiling of sputum for the detection of early and locally advanced non-small-cell lung cancer: a prospective case–control study. Curr Oncol 2016, 23, 86–94, doi:10.3747/co.23.2830.
- Roa, W.H.; Kim, J.O.; Razzak, R.; Du, H.; Guo, L.; Singh, R.; Gazala, S.; Ghosh, S.; Wong, E.; Joy, A.A.; et al. Sputum microRNA profiling: A novel approach for the early detection of non-small cell lung cancer. Clin Invest Med 2012, 35, E271-E281, doi:10.25011/cim.v35i5.18700.
- Sheervalilou, R.; Khamaneh, A.M.; Sharifi, A.; Nazemiyeh, M.; Taghizadieh, A.; Ansarin, K.; Zarghami, N. Using miR-10b, miR-1 and miR-30a expression profiles of bronchoalveolar lavage and sputum for early detection of non-small cell lung cancer. Biomed Pharmacother 2017, 88, 1173–1182, doi:10.1016/j.biopha.2017.02.002.
- Shen, J.; Liao, J.; Guarnera, M.A.; Fang, H.; Cai, L.; Stass, S.A.; Jiang, F. Analysis of MicroRNAs in sputum to improve computed tomography for lung cancer diagnosis. J Thorac Oncol 2014, 9, 33–40, doi:10.1097/JTO.0000000000000025.
- Su, J.; Anjuman, N.; Guarnera, M.A.; Zhang, H.; Stass, S.A.; Jiang, F. Analysis of lung flute-collected sputum for lung cancer diagnosis. Biomark Insights 2015, 10, doi:10.4137/bmi.S26883.
- Su, J.; Leng, Q.; Lin, Y.; Ma, J.; Jiang, F.; Lee, C.-J.; Fang, H.; Jiang, F. Integrating circulating immunological and sputum biomarkers for the early detection of lung cancer. Biomark Cancer 2018, 10, doi:10.1177/1179299x18759297.
- Su, J.; Liao, J.; Gao, L.; Shen, J.; Guarnera, M.A.; Zhan, M.; Fang, H.; Stass, S.A.; Jiang, F. Analysis of small nucleolar RNAs in sputum for lung cancer diagnosis. Oncotarget 2016, 7, 5131–5142, doi:10.18632/oncotarget.4219.
- Su, Y.; Guarnera, M.A.; Fang, H.; Jiang, F. Small non-coding RNA biomarkers in sputum for lung cancer diagnosis. Mol Cancer 2016, 15, doi:10.1186/s12943-016-0520-8.
- Tellez, C.S.; Juri, D.E.; Do, K.; Picchi, M.A.; Wang, T.; Liu, G.; Spira, A.; Belinsky, S.A. miR-196b is epigenetically silenced during the premalignant stage of lung carcinogenesis. Cancer Res 2016, 76, 4741–4751, doi:10.1158/0008-5472.Can-15-3367.
- Xie, Y.; Todd, N.W.; Liu, Z.; Zhan, M.; Fang, H.; Peng, H.; Alattar, M.; Deepak, J.; Stass, S.A.; Jiang, F. Altered miRNA expression in sputum for diagnosis of non-small cell lung cancer. Lung Cancer 2010, 67, 170–176, doi:10.1016/j.lungcan.2009.04.004.
- Xing, L.; Su, J.; Guarnera, M.A.; Zhang, H.; Cai, L.; Zhou, R.; Stass, S.A.; Jiang, F. Sputum microRNA biomarkers for identifying lung cancer in indeterminate solitary pulmonary nodules. Clin Cancer Res 2015, 21, 484–489, doi:10.1158/1078-0432.Ccr-14-1873.
- Xing, L.; Todd, N.W.; Yu, L.; Fang, H.; Jiang, F. Early detection of squamous cell lung cancer in sputum by a panel of microRNA markers. Mod Pathol 2010, 23, 1157–1164, doi:10.1038/modpathol.2010.111.
- Yazdanpour, M.; Rahmani, S.; Bayat, H.; Mirtavoos-Mahyari, H.; Khosravi, A.; Mowla, S.J. Non-invasive discrimination of adenocarcinoma and squamous cell carcinoma based on differential expression of miR-944 and miR-326 in sputum samples of lung cancer patients. Hum Gene (Amst) 2024, 40, doi:10.1016/j.humgen.2024.201273.
- Yu, L.; Todd, N.W.; Xing, L.; Xie, Y.; Zhang, H.; Liu, Z.; Fang, H.; Zhang, J.; Katz, R.L.; Jiang, F. Early detection of lung adenocarcinoma in sputum by a panel of microRNA markers. Int J Cancer 2010, 127, 2870–2878, doi:10.1002/ijc.25289.

### Appendix A.5. Proteomics

- Ajona, D.; Razquin, C.; Pastor, M.D.; Pajares, M.J.; Garcia, J.; Cardenal, F.; Fleischhacker, M.; Lozano, M.D.; Zulueta, J.J.; Schmidt, B.; et al. Elevated levels of the complement activation product C4d in bronchial fluids for the diagnosis of lung cancer. PLoS One 2015, 10, e0119878, doi:10.1371/journal.pone.0119878.
- Ali-Labib, R.; Louka, M.L.; Galal, I.H.; Tarek, M. Evaluation of matrix metalloproteinase-2 in lung cancer. Proteomics Clin Appl 2014, 8, 251–257, doi:10.1002/prca.201300086.
- Arenas-De Larriva, M.D.S.; Fernandez-Vega, A.; Jurado-Gamez, B.; Ortea, I. diaPASEF proteomics and feature selection for the description of sputum proteome profiles in a cohort of different subtypes of lung cancer patients and controls. Int J Mol Sci 2022, 23, doi:10.3390/ijms23158737.
- Bar-Shai, A.; Shenhar-Tsarfaty, S.; Ahimor, A.; Ophir, N.; Rotem, M.; Alcalay, Y.; Fireman, E. A novel combined score of biomarkers in sputum may be an indicator for lung cancer: A pilot study. Clin Chim Acta 2018, 487, 139–144, doi:10.1016/j.cca.2018.09.027.
- Bottger, F.; Schaaij-Visser, T.B.; de Reus, I.; Piersma, S.R.; Pham, T.V.; Nagel, R.; Brakenhoff, R.H.; Thunnissen, E.; Smit, E.F.; Jimenez, C.R. Proteome analysis of non-small cell lung cancer cell line secretomes and patient sputum reveals biofluid biomarker candidates for cisplatin response prediction. J Proteomics 2019, 196, 106–119, doi:10.1016/j.jprot.2019.01.018.
- Carpagnano, G.E.; Palladino, G.P.; Lacedonia, D.; Koutelou, A.; Orlando, S.; Foschino-Barbaro, M.P. Neutrophilic airways inflammation in lung cancer: the role of exhaled LTB-4 and IL-8. BMC Cancer 2011, 11, 226, doi:10.1186/1471-2407-11-226.
- Carpagnano, G.E.; Spanevello, A.; Curci, C.; Salerno, F.; Palladino, G.P.; Resta, O.; Di Gioia, G.; Carpagnano, F.; Foschino Barbaro, M.P. IL-2, TNF-alpha, and leptin: local versus systemic concentrations in NSCLC patients. Oncol Res 2007, 16, 375–381, doi:10.3727/000000006783980900.
- Du, Y.J.; Cao, Y.X.; Duan, X.H. [Study on the correlation between the inflammatory factors in the serum and the induced sputum and the hypothalamic-pituitary-adrenal axis function in advanced lung adenocarcinoma of different syndromes]. Zhongguo Zhong Xi Yi Jie He Za Zhi 2012, 32, 896–901.
- Ganjali, M.; Kheirkhah, B.; Amini, K. Expression of miRNA-601 and PD-L1 among Iranian patients with lung cancer and their relationship with smoking and *Mycoplasma* infection. Cell J 2021, 23, 723–729, doi:10.22074/cellj.2021.7704.
- Hillas, G.; Moschos, C.; Dimakou, K.; Vlastos, F.; Avgeropoulou, S.; Christakopoulou, I.; Rasidakis, A.; Bakakos, P. Carcinoembryonic antigen, neuron-specific enolase and cytokeratin fragment 19 (CYFRA 21-1) levels in induced sputum of lung cancer patients. Scand J Clin Lab Invest 2008, 68, 542–547, doi:10.1080/00365510701883172.
- Jheon, S.; Hyun, D.S.; Lee, S.C.; Yoon, G.S.; Jeon, C.H.; Park, J.W.; Park, C.K.; Jung, M.H.; Lee, K.D.; Chang, H.K. Lung cancer detection by a RT-nested PCR using MAGE A1--6 common primers. Lung Cancer 2004, 43, 29–37, doi:10.1016/j.lungcan.2003.08.014.
- Josyula, A.B.; Poplin, G.S.; Kurzius-Spencer, M.; McClellen, H.E.; Kopplin, M.J.; Sturup, S.; Clark Lantz, R.; Burgess, J.L. Environmental arsenic exposure and sputum metalloproteinase concentrations. Environ Res 2006, 102, 283–290, doi:10.1016/j.envres.2006.01.003.
- Li, N.; Holden, V.K.; Deepak, J.; Todd, N.W.; Jiang, F. Autoantibodies against tumor-associated antigens in sputum as biomarkers for lung cancer. Transl Oncol 2021, 14, 100991, doi:10.1016/j.tranon.2020.100991.
- Masilamani, V.; Trinka, V.; Al Salhi, M.; Elangovan, M.; Raghavan, V.; Al Diab, A.R.; Hajjar, W.; Ainia, M.; Al-Mustafa, A.; Al-Nachawati, H. A new lung cancer biomarker--a preliminary report. Photomed Laser Surg 2011, 29, 161–170, doi:10.1089/pho.2009.2615.
- Mumford, J.L.; Tian, D.; Younes, M.; Hu, F.; Lan, Q.; Ostrowski, M.L.; He, X.; Feng, Z. Detection of p53 protein accumulation in sputum and lung adenocarcinoma associated with indoor exposure to unvented coal smoke in China. Anticancer Res 1999, 19, 951–958.
- Niklinski, J.; Chyczewski, L.; Chyczewska, E.; Laudanski, J.; Furman, M. A correlative study of bronchial cytology, bronchoalveolar lavage (BAL) and serum tumor markers in the diagnosis of lung carcinoma. Folia Histochem Cytobiol 1993, 31, 211–213.
- Panakkal, N.; Lekshmi, A.; Krishna, K.M.J.; Saraswathy, V.V.; Sujathan, K. Expression of minichromosome maintenance proteins in the exfoliated cells supplement sputum cytology in the diagnosis of lung cancer. Cytojournal 2024, 21, 81, doi:10.25259/Cytojournal_115_2024.
- Pio, R.; Garcia, J.; Corrales, L.; Ajona, D.; Fleischhacker, M.; Pajares, M.J.; Cardenal, F.; Seijo, L.; Zulueta, J.J.; Nadal, E.; et al. Complement factor H is elevated in bronchoalveolar lavage fluid and sputum from patients with lung cancer. Cancer Epidemiol Biomarkers Prev 2010, 19, 2665–2672, doi:10.1158/1055-9965.EPI-10-0467.
- Qiao, Y.L.; Tockman, M.S.; Li, L.; Erozan, Y.S.; Yao, S.X.; Barrett, M.J.; Zhou, W.H.; Giffen, C.A.; Luo, X.C.; Taylor, P.R. A case-cohort study of an early biomarker of lung cancer in a screening cohort of Yunnan tin miners in China. Cancer Epidemiol Biomarkers Prev 1997, 6, 893–900.
- Rangel, M.P.; de Sa, V.K.; Martins, V.; Martins, J.R.; Parra, E.R.; Mendes, A.; Andrade, P.C.; Reis, R.M.; Longatto-Filho, A.; Oliveira, C.Z.; et al. Tissue hyaluronan expression, as reflected in the sputum of lung cancer patients, is an indicator of malignancy. Braz J Med Biol Res 2015, 48, 557–567, doi:10.1590/1414-431X20144300.
- Rostila, A.M.; Anttila, S.L.; Lalowski, M.M.; Vuopala, K.S.; Toljamo, T.I.; Lindstrom, I.; Baumann, M.H.; Puustinen, A.M. Reactive oxygen species-regulating proteins peroxiredoxin 2 and thioredoxin, and glyceraldehyde-3-phosphate dehydrogenase are differentially abundant in induced sputum from smokers with lung cancer or asbestos exposure. Eur J Cancer Prev 2020, 29, 238–247, doi:10.1097/CEJ.0000000000000537.
- Roychoudhury, S.; Mondal, N.K.; Mukherjee, S.; Dutta, A.; Siddique, S.; Ray, M.R. Activation of protein kinase B (PKB/Akt) and risk of lung cancer among rural women in India who cook with biomass fuel. Toxicol Appl Pharmacol 2012, 259, 45–53, doi:10.1016/j.taap.2011.12.002.
- Setoguchi, Y.; Kato, M.; Shoji, M.; Honjoh, T.; Kamei, M.; Sugitani, M.; Sato, S.; Hashizume, S.; Hanagiri, T.; Yoshimatsu, T.; et al. Immunocytochemical detection of lung cancer cells with monoclonal antibodies to 14-3-3 proteins. Hum Antibodies Hybridomas 1995, 6, 137–144.
- Shaham, J.; Fireman, E.; Korenstein-Ilan, A.; Lerman, Y. Detection of p53 protein in induced sputum after occupational exposure to crystalline silica. J Occup Environ Med 2007, 49, 730–735, doi:10.1097/JOM.0b013e31805d0be4.
- Shivapurkar, N.; Stastny, V.; Okumura, N.; Girard, L.; Xie, Y.; Prinsen, C.; Thunnissen, F.B.; Wistuba, I.I.; Czerniak, B.; Frenkel, E.; Roth, J.A. Cytoglobin, the newest member of the globin family, functions as a tumor suppressor gene. Cancer Res 2008, 68, 7448–7456, doi:10.1158/0008-5472.CAN-08-0565.
- Shoji, M.; Kawamoto, S.; Seki, K.; Teruya, K.; Setoguchi, Y.; Mochizuki, K.; Kato, M.; Hashizume, S.; Hanagiri, T.; Yoshimatsu, T.; et al. Lung cancer-reacting human recombinant antibody AE6F4: potential usefulness in the sputum cytodiagnosis. Hum Antibodies Hybridomas 1996, 7, 27–36.
- Sueoka, E.; Sueoka, N.; Goto, Y.; Matsuyama, S.; Nishimura, H.; Sato, M.; Fujimura, S.; Chiba, H.; Fujiki, H. Heterogeneous nuclear ribonucleoprotein B1 as early cancer biomarker for occult cancer of human lungs and bronchial dysplasia. Cancer Res 2001, 61, 1896–1902.
- Sun, B.; Wang, H.; Wang, X.; Huang, H.; Ding, W.; Jing, R.; Shi, G.; Zhu, L. A proliferation-inducing ligand: a new biomarker for non-small cell lung cancer. Exp Lung Res 2009, 35, 486–500, doi:10.1080/01902140902759274.
- Tierney, L.A.; Neft, R.E.; Belinsky, S.A.; Lauer, F.T.; Gilliland, F.D.; Crowell, R.E.; Lechner, J.F. Double-label suspension immunohistochemistry for the detection of gene dysfunction in sputum. J Histotechnol 2013, 20, 13–17, doi:10.1179/his.1997.20.1.13.
- Tockman, M.S.; Gupta, P.K.; Myers, J.D.; Frost, J.K.; Baylin, S.B.; Gold, E.B.; Chase, A.M.; Wilkinson, P.H.; Mulshine, J.L. Sensitive and specific monoclonal antibody recognition of human lung cancer antigen on preserved sputum cells: a new approach to early lung cancer detection. J Clin Oncol 1988, 6, 1685–1693, doi:10.1200/JCO.1988.6.11.1685.
- Tockman, M.S.; Mulshine, J.L.; Piantadosi, S.; Erozan, Y.S.; Gupta, P.K.; Ruckdeschel, J.C.; Taylor, P.R.; Zhukov, T.; Zhou, W.H.; Qiao, Y.L.; et al. Prospective detection of preclinical lung cancer: results from two studies of heterogeneous nuclear ribonucleoprotein A2/B1 overexpression. Clin Cancer Res 1997, 3, 2237–2246.
- Trubnikov, G.A.; Sukharev, A.E.; Uklistaia, T.A.; Orlova, E.A. [Clinico-diagnostic role of ferritin and lactoferrin assays in benign and malignant affections of lungs and pleura]. Klin Med (Mosk) 1998, 76, 21–26.
- Veena, V.S.; Rajan, K.; Saritha, V.N.; George, P.S.; Chandramohan, K.; Jayasree, K.; Thara, S.; Sujathan, K. DNA replication licensing proteins for early detection of lung cancer. Asian Pac J Cancer Prev 2017, 18, 3041–3047, doi:10.22034/APJCP.2017.18.11.3041.
- Veena, V.S.; George, P.S.; Rajan, K.; Chandramohan, K.; Jayasree, K.; Sujathan, K. Immunocytochemistry on sputum samples predicts prognosis of lung cancer. J Cytol 2019, 36, 38–43, doi:10.4103/JOC.JOC_103_17.
- Veena, V.S.; Saritha, V.N.; George, P.S.; Rajan, K.; Jayasree, K.; Sujathan, K. Immunoexpression of TTF1 and p63 differentiates lung adenocarcinomas in sputum samples. J Cytol 2021, 38, 151–157, doi:10.4103/JOC.JOC_252_16.
- Wang, J.; Qiao, X.Y.; Lu, F.H.; Zhou, Z.G.; Song, Y.Z.; Huo, J.J.; Liu, X. TGF-beta1 in serum and induced sputum for predicting radiation pneumonitis in patients with non-small cell lung cancer after radiotherapy. Chin J Cancer 2010, 29, 325–329, doi:10.5732/cjc.009.10454.
- Wu, C.; Li, W.; Xu, D.; Chen, W.; Wei, D.; Zhou, Q. [Expression and value of hnRNP B1 in sputum of patients with lung cancer by immunohistochemistry method]. Zhongguo Fei Ai Za Zhi 2006, 9, 428–430, doi:10.3779/j.issn.1009-3419.2006.05.07.
- Yang, S.W.; Choi, P.R.; You, H.J.; Kim, J.G.; Oak, C.H.; Jang, T.W.; Jung, M.H. Relation between ERCC1 expression in sputum and survival after cisplatin-based chemotherapy in patients with non-small cell lung cancer. Tuberc Respir Dis (Seoul) 2006, 60, 151–159, doi:10.4046/trd.2006.60.2.151.
- Yook, D.S.; Shin, H.-S.; Choi, P.; Kim, J.-H.; Shin, S.-H.; Ok, C.-H.; Cho, H.-M.; Jang, T.-W.; Jung, M.-H.; Park, J.-W.; et al. Expression of MAGE in the induced sputum of lung cancer patients. Tuberc Respir Dis (Seoul) 2002, 53, 265–274, doi:10.4046/trd.2002.53.3.265.
- Yu, L.; Shen, J.; Mannoor, K.; Guarnera, M.; Jiang, F. Identification of ENO1 as a potential sputum biomarker for early-stage lung cancer by shotgun proteomics. Clin Lung Cancer 2014, 15, 372–378 e371, doi:10.1016/j.cllc.2014.05.003.
- Zhang, R.; Zhang, J.; Tan, F.; Yang, D.; Wang, B.; Dai, J.; Qi, Y.; Ran, L.; He, W.; Lv, Y.; et al. Multi-channel AgNWs-doped interdigitated organic electrochemical transistors enable sputum-based device towards noninvasive and portable diagnosis of lung cancer. Mater Today Bio 2022, 16, 100385, doi:10.1016/j.mtbio.2022.100385.

### Appendix A.6. Metabolomics

- Ahmed, N.; Bezabeh, T.; Ijare, O.B.; Myers, R.; Alomran, R.; Aliani, M.; Nugent, Z.; Banerji, S.; Kim, J.; Qing, G.; et al. Metabolic signatures of lung cancer in sputum and exhaled breath condensate detected by (1)H magnetic resonance spectroscopy: a feasibility study. Magn Reson Insights 2016, 9, 29–35, doi:10.4137/MRI.S40864.
- Ahmed, N.; Kidane, B.; Wang, L.; Nugent, Z.; Moldovan, N.; McElrea, A.; Shariati-Ievari, S.; Qing, G.; Tan, L.; Buduhan, G.; et al. Metabolic alterations in sputum and exhaled breath condensate of early stage non-small cell lung cancer patients after surgical resection: a pilot study. Front Oncol 2022, 12, 874964, doi:10.3389/fonc.2022.874964.
- Ahmed, N.; Kidane, B.; Wang, L.; Qing, G.; Tan, L.; Buduhan, G.; Srinathan, S.; Aliani, M. Non-invasive exploration of metabolic profile of lung cancer with magnetic resonance spectroscopy and mass spectrometry. Contemp Clin Trials Commun 2019, 16, 100445, doi:10.1016/j.conctc.2019.100445.
- Ardatskaya, M.D.; Ponomareva, E.V.; Shevtsov, V.V.; Evdokimova, S.A.; Odintsov, S.V. Diagnostic and tactical importance of studying short chain fatty acids in different biological substrates took place in patients with chronic obstructive pulmonary disease, lung cancer and community-acquired pneumonia developed after anticancer therapy. Eksp Klin Gastroenterol 2016, 17–25.
- Cameron, S.J.; Lewis, K.E.; Beckmann, M.; Allison, G.G.; Ghosal, R.; Lewis, P.D.; Mur, L.A. The metabolomic detection of lung cancer biomarkers in sputum. Lung Cancer 2016, 94, 88–95, doi:10.1016/j.lungcan.2016.02.006.
- Gao, X.F.; Xiao, Y.; Dai, Y. Direct analysis of human sputum for differentiating non-small cell lung cancer by neutral desorption extractive electrospray ionization mass spectrometry. Anal Sci 2018, 34, 1067–1071, doi:10.2116/analsci.18P008.
- Lewis, P.D.; Lewis, K.E.; Ghosal, R.; Bayliss, S.; Lloyd, A.J.; Wills, J.; Godfrey, R.; Kloer, P.; Mur, L.A. Evaluation of FTIR spectroscopy as a diagnostic tool for lung cancer using sputum. BMC Cancer 2010, 10, 640, doi:10.1186/1471-2407-10-640.
- O’Shea, K.; Cameron, S.J.; Lewis, K.E.; Lu, C.; Mur, L.A. Metabolomic-based biomarker discovery for non-invasive lung cancer screening: A case study. Biochim Biophys Acta 2016, 1860, 2682–2687, doi:10.1016/j.bbagen.2016.07.007.
- Zhang, J.; Xu, J.; Lu, H.; Ding, J.; Yu, D.; Li, P.; Xiong, J.; Liu, X.; Chen, H.; Wei, Y. Altered phosphatidylcholines expression in sputum for diagnosis of non-small cell lung cancer. Oncotarget 2016, 7, 63158–63165, doi:10.18632/oncotarget.11283.
- Zheng, Q.; Zhang, J.; Wang, X.; Zhang, W.; Xiao, Y.; Hu, S.; Xu, J. Neutral desorption extractive electrospray ionization mass spectrometry analysis sputum for non-invasive lung adenocarcinoma detection. Onco Targets Ther 2021, 14, 469–479, doi:10.2147/OTT.S269300.

### Appendix A.7. Integromics (Integrative Multi-Omics)

- Baryshnikova, E.; Destro, A.; Infante, M.V.; Cavuto, S.; Cariboni, U.; Alloisio, M.; Ceresoli, G.L.; Lutman, R.; Brambilla, G.; Chiesa, G.; et al. Molecular alterations in spontaneous sputum of cancer-free heavy smokers: results from a large screening program. Clin Cancer Res 2008, 14, 1913–1919, doi:10.1158/1078-0432.CCR-07-1741.
- Carozzi, F.M.; Bisanzi, S.; Carrozzi, L.; Falaschi, F.; Lopes Pegna, A.; Mascalchi, M.; Picozzi, G.; Peluso, M.; Sani, C.; Greco, L.; et al. Multimodal lung cancer screening using the ITALUNG biomarker panel and low dose computed tomography. Results of the ITALUNG biomarker study. Int J Cancer 2017, 141, 94–101, doi:10.1002/ijc.30727.
- Hsu, H.S.; Chen, T.P.; Wen, C.K.; Hung, C.H.; Chen, C.Y.; Chen, J.T.; Wang, Y.C. Multiple genetic and epigenetic biomarkers for lung cancer detection in cytologically negative sputum and a nested case-control study for risk assessment. J Pathol 2007, 213, 412–419, doi:10.1002/path.2246.
- Kersting, M.; Friedl, C.; Kraus, A.; Behn, M.; Pankow, W.; Schuermann, M. Differential frequencies of p16(INK4a) promoter hypermethylation, p53 mutation, and K-ras mutation in exfoliative material mark the development of lung cancer in symptomatic chronic smokers. J Clin Oncol 2000, 18, 3221–3229, doi:10.1200/JCO.2000.18.18.3221.
- Li, N.; Dhilipkannah, P.; Jiang, F. High-throughput detection of multiple miRNAs and methylated DNA by droplet digital PCR. J Pers Med 2021, 11, doi:10.3390/jpm11050359.
- Liao, J.; Dhilipkannah, P.; Jiang, F. Improving CT scan for lung cancer diagnosis with an integromic signature. J Biol Methods 2024, 11, e99010023, doi:10.14440/jbm.2024.0028.
- Shin, K.C.; Lee, K.H.; Lee, C.H.; Shin, I.H.; Suh, H.S.; Jeon, C.H. MAGE A1-A6 RT-PCR and MAGE A3 and p16 methylation analysis in induced sputum from patients with lung cancer and non-malignant lung diseases. Oncol Rep 2012, 27, 911–916, doi:10.3892/or.2011.1566.
- Su, Y.; Fang, H.; Jiang, F. Integrating DNA methylation and microRNA biomarkers in sputum for lung cancer detection. Clin Epigenetics 2016, 8, 109, doi:10.1186/s13148-016-0275-5.
- Wang, Y.C.; Hsu, H.S.; Chen, T.P.; Chen, J.T. Molecular diagnostic markers for lung cancer in sputum and plasma. Ann N Y Acad Sci 2006, 1075, 179–184, doi:10.1196/annals.1368.024.

### Appendix A.8. Metagenomics and Microbiome

- Baranova, E.; Druzhinin, V.; Matskova, L.; Demenkov, P.; Volobaev, V.; Larionov, A. Comparison of sputum and oropharyngeal microbiome compositions in patients with non-small cell lung cancer. OBM Genetics 2022, 06, 1–23, doi:10.21926/obm.genet.2204169.
- Baranova, E.; Druzhinin, V.; Matskova, L.; Demenkov, P.; Volobaev, V.; Minina, V.; Larionov, A.; Titov, V. Sputum microbiome composition in patients with squamous cell lung carcinoma. Life (Basel) 2022, 12, doi:10.3390/life12091365.
- Cameron, S.J.S.; Lewis, K.E.; Huws, S.A.; Hegarty, M.J.; Lewis, P.D.; Pachebat, J.A.; Mur, L.A.J. A pilot study using metagenomic sequencing of the sputum microbiome suggests potential bacterial biomarkers for lung cancer. PLoS One 2017, 12, e0177062, doi:10.1371/journal.pone.0177062.
- Dhilipkannah, P.; Sachdeva, A.; Holden, V.K.; Jiang, F. Integrative biomarker panel for improved lung cancer diagnosis using plasma microRNAs and sputum bacterial DNA. Curr Oncol 2024, 31, 5949–5959, doi:10.3390/curroncol31100444.
- Druzhinin, V.G.; Baranova, E.D.; Demenkov, P.S.; Matskova, L.V.; Larionov, A.V. Composition of the sputum bacterial microbiome of patients with different pathomorphological forms of non-small-cell lung cancer. Vavilovskii Zhurnal Genet Selektsii 2024, 28, 204–214, doi:10.18699/vjgb-24-25.
- Druzhinin, V.G.; Matskova, L.V.; Demenkov, P.S.; Baranova, E.D.; Volobaev, V.P.; Minina, V.I.; Apalko, S.V.; Churina, M.A.; Romanyuk, S.A.; Shcherbak, S.G.; et al. Taxonomic diversity of sputum microbiome in lung cancer patients and its relationship with chromosomal aberrations in blood lymphocytes. Sci Rep 2020, 10, 9681, doi:10.1038/s41598-020-66654-x.
- Druzhinin, V.G.; Matskova, L.V.; Demenkov, P.S.; Baranova, E.D.; Volobaev, V.P.; Minina, V.I.; Larionov, A.V.; Titov, V.A.; Fucic, A. Genetic damage in lymphocytes of lung cancer patients is correlated to the composition of the respiratory tract microbiome. Mutagenesis 2021, 36, 143–153, doi:10.1093/mutage/geab004.
- Druzhinin, V.G.; Baranova, E.D.; Demenkov, P.S.; Matskova, L.V.; Larionov, A.V.; Yuzhalin, A.E. Lower respiratory tract microbiome signatures of health and lung cancer across different smoking statuses. Cancers (Basel) 2025, 17, 2643, doi:10.3390/cancers17162643.
- He, J.Q.; Chen, Q.; Wu, S.J.; Wang, D.Q.; Zhang, S.Y.; Zhang, S.Z.; Chen, R.L.; Wang, J.F.; Wang, Z.; Yu, C.H. Potential implications of the lung microbiota in patients with chronic obstruction pulmonary disease and non-small cell lung cancer. Front Cell Infect Microbiol 2022, 12, 937864, doi:10.3389/fcimb.2022.937864.
- Hosgood, H.D., 3rd; Mongodin, E.F.; Wan, Y.; Hua, X.; Rothman, N.; Hu, W.; Vermeulen, R.; Seow, W.J.; Rohan, T.; Xu, J.; et al. The respiratory tract microbiome and its relationship to lung cancer and environmental exposures found in rural China. Environ Mol Mutagen 2019, 60, 617–623, doi:10.1002/em.22291.
- Hosgood, H.D., 3rd; Sapkota, A.R.; Rothman, N.; Rohan, T.; Hu, W.; Xu, J.; Vermeulen, R.; He, X.; White, J.R.; Wu, G.; et al. The potential role of lung microbiota in lung cancer attributed to household coal burning exposures. Environ Mol Mutagen 2014, 55, 643–651, doi:10.1002/em.21878.
- Huang, D.; Ren, Q.; Xie, L.; Chen, Y.; Li, C.; Su, X.; Lin, L.; Liu, L.; Zhao, H.; Luo, T.; et al. Association between airway microbiota and systemic inflammation markers in non-small cell lung cancer patients. Sci Rep 2025, 15, 3539, doi:10.1038/s41598-025-86231-4.
- Huang, D.; Su, X.; Yuan, M.; Zhang, S.; He, J.; Deng, Q.; Qiu, W.; Dong, H.; Cai, S. The characterization of lung microbiome in lung cancer patients with different clinicopathology. Am J Cancer Res 2019, 9, 2047–2063.
- Huang, D.H.; He, J.; Su, X.F.; Wen, Y.N.; Zhang, S.J.; Liu, L.Y.; Zhao, H.; Ye, C.P.; Wu, J.H.; Cai, S.; et al. The airway microbiota of non-small cell lung cancer patients and its relationship to tumor stage and EGFR gene mutation. Thorac Cancer 2022, 13, 858–869, doi:10.1111/1759-7714.14340.
- Li, S.; Zhan, Y.; Wang, Y.; Li, W.; Wang, X.; Wang, H.; Sun, W.; Cao, X.; Li, Z.; Ye, F. One-step diagnosis of infection and lung cancer using metagenomic sequencing. Respir Res 2025, 26, 48, doi:10.1186/s12931-025-03127-7.
- Lu, H.; Gao, N.L.; Tong, F.; Wang, J.; Li, H.; Zhang, R.; Ma, H.; Yang, N.; Zhang, Y.; Wang, Y.; et al. Alterations of the human lung and gut microbiomes in non-small cell lung carcinomas and distant metastasis. Microbiol Spectr 2021, 9, e0080221, doi:10.1128/Spectrum.00802-21.
- Zapata-García, M.; Moratiel-Pellitero, A.; Isla, D.; Gálvez, E.; Gascón-Ruiz, M.; Sesma, A.; Barbero, R.; Galeano, J.; Del Campo, R.; Ocáriz, M.; Quílez, E. Impact of antibiotics, corticosteroids, and microbiota on immunotherapy efficacy in patients with non-small cell lung cancer. Heliyon 2024, 10, e33684, doi:10.1016/j.heliyon.2024.e33684.
- Zhang, C.; Wang, J.; Sun, Z.; Cao, Y.; Mu, Z.; Ji, X. Commensal microbiota contributes to predicting the response to immune checkpoint inhibitors in non-small-cell lung cancer patients. Cancer Sci 2021, 112, 3005–3017, doi:10.1111/cas.14979.
- Zhang, L., Li, M.J., Li, X.P., Yang, B., Xiao, T., Wang, P., Zhang, W.D. Respiratory microbiota diversity as a predictive biomarker for the efficacy of PD-1 blockades in patients with advanced non-small cell lung cancer: A retrospective exploratory study. Oncol Lett 2025, 29, 251, doi:10.3892/ol.2025.14997.

### Appendix A.9. Sputum Collection, Storage, and Processing

- Bano, A.; Yadav, P.; Sharma, M.; Verma, D.; Vats, R.; Chaudhry, D.; Kumar, P.; Bhardwaj, R. Extraction and characterization of exosomes from the exhaled breath condensate and sputum of lung cancer patients and vulnerable tobacco consumers-potential noninvasive diagnostic biomarker source. J Breath Res 2024, 18, doi:10.1088/1752-7163/ad5eae.
- Frost, J.K.; Tyrer, H.W.; Pressman, N.J.; Albright, C.D.; Vansickel, M.H.; Gill, G.W. Automatic cell identification and enrichment in lung cancer. I. Light scatter and fluorescence parameters. J Histochem Cytochem 1979, 27, 545–551, doi:10.1177/27.1.86575.
- Gottschall, E.B.; McGinley, J.N.; Spoelstra, N.; Knott, K.; Wolfe, P.; Rose, C.; Singh, M.; Thompson, H.J. Effect of cytological fixative and environmental conditions on nuclear morphometric characteristics of squamous epithelial cells in sputum. Cytometry B Clin Cytom 2005, 67, 19–26, doi:10.1002/cyto.b.20060.
- Grayson, M.; Lai, S.C.; Bederka, L.H.; Araujo, P.; Sanchez, J.; Reveles, X.T.; Rebel, V.I.; Rebeles, J. Quality-controlled sputum analysis by flow cytometry. J Vis Exp 2021, 9, e62785, doi:10.3791/62785.
- Kraemer, P.S.; Sanchez, C.A.; Goodman, G.E.; Jett, J.; Rabinovitch, P.S.; Reid, B.J. Flow cytometric enrichment for respiratory epithelial cells in sputum. Cytometry A 2004, 60, 1–7, doi:10.1002/cyto.a.20041.
- Ma, Y.; Wang, Y.; He, L.; Du, J.; Li, L.; Bie, Z.; Li, Y.; Xu, X.; Zhou, W.; Wu, X.; et al. Preservation of cfRNA in cytological supernatants for cfDNA & cfRNA double detection in non-small cell lung cancer patients. Cancer Med 2024, 13, e70197, doi:10.1002/cam4.70197.
- van der Drift, M.A.; Prinsen, C.F.; Hol, B.E.; Bolijn, A.S.; Jeunink, M.A.; Dekhuijzen, P.N.; Thunnissen, F.B. Can free DNA be detected in sputum of lung cancer patients? Lung Cancer 2008, 61, 385–390, doi:10.1016/j.lungcan.2008.01.007.
