# Supplementary material for: Sputum Liquid Biopsy for Lung Cancer Screening, Diagnosis, Subtyping, Surveillance, Response Prediction, and Prognostication: A Scoping Review
Source: Med Sci (Basel). 2026 Apr 30;14(2):231. doi: 10.3390/medsci14020231 (PMC13214846; doi:10.3390/medsci14020231)
Supplement: Supplementary file 1 [file medsci-14-00231-s001.zip › medsci-4263487-supplementary.pdf]

Supplementary Table S1. Overall summary and translational readiness of sputum biomarkers across all domains included in the scoping review.

Domain	Number of studies	Potential clinical use cases	Candidate biomarkers and/or techniques	Summary of findings	Translational maturity*
Cytopathology	49	Diagnosis<br>Screening<br>Histologic subtyping<br>Actionable alterations<br>Prognostication	Flow cytometry<br>MACS<br>Papanicolaou smear<br>Quantitative microscopy (LungSign®, Perceptronix Medical Inc.; Vancouver, BC, Canada), automated DNA cytometry, 3D morphologic cytometry (LuCED®, VisionGate Inc.; Phoenix, AZ, USA), PWS microscopy<br>Porphyrin-labeling (CyPath®, bioAffinity Technologies, Inc.; San Antonio, TX, USA)<br>FISH for MSI<br>CA-FISH panel<br>*EGFR* copy number assessment<br>FAL-FISH panel<br>TRAP	Foundational domain with the longest track record. FISH- and cytometry-based approaches generally outperform conventional cytology in enriched populations, and automated flow/cytometric approaches are among the most mature sputum assays; however, performance remains variable across platforms and settings	Phase 2–5 (most mature: Papanicolaou smear, Phase 5; porphyrin-labeling/flow cytometry Phase 4–5)
Genomics	49	Diagnosis<br>Screening<br>Actionable alterations<br>Monitoring response<br>Prognostication	*EGFR* mutations<br>*BRAF* mutation<br>*K-RAS* mutation<br>*TP53* mutation<br>*EML4-ALK* fusion<br>PD-L1 status<br>*HER2*, *ROS1*, *RET*, *MET*, *NTRK* and *NRG* gene alterations<br>Multiplex ddPCR panels	Tumor-related genomic alterations can be detected noninvasively in sputum. Performance is strongest in advanced disease, cytology-positive samples, and sputum supernatant cfDNA; multi-gene panels outperform single-gene assays, but real-world validation is still limited.	Phase 2–4

NGS profiling					
Methylomics and epigenetics	57	Diagnosis<br>Screening<br>Prognostication	ddMSP panels (p16INK4a, *RASSF1A*, *SOX17*, *TAC1*, etc.)<br>3D quantitative DNA topology imaging	Largest and one of the most mature molecular domains. Multi-gene methylation panels generally outperform single loci and can identify cancer risk before diagnosis, but specificity is constrained by airway field cancerization and methodological heterogeneity.	Phase 2–4 (most promising ddMSP panels: Phase 3–4)
Proteomics	41	Diagnosis<br>Screening<br>Histologic subtyping<br>Actionable alterations<br>Monitoring response<br>Surveillance<br>Prognostication	Cell-block IHC for tumor markers<br>Single and multiple protein panels (SELDI-TOF/MALDI-TOF and ELISA)<br>diaPASEF (MS) profiling<br>EV-derived proteome profiling, similar to ExoDx™ Lung(ALK) [Exosome Diagnostics Inc.; Waltham, MA, USA]<br>Portable biosensors (multichannel electrochemical transistor technology)	Protein biomarkers show signal across diagnosis, subtyping, and treatment-response assessment. Cell-block IHC and multiplex biosensors appear promising, while discovery proteomics and secretome approaches remain early-phase and need external validation.	Phase 2–4
Transcriptomics	29	Diagnosis<br>Screening<br>Subtyping<br>Monitoring response<br>Prognostication	Survivin mRNA<br>*hTERT* mRNA<br>Adenocarcinoma 4-miRNA panel<br>Squamous cell carcinoma 3-miRNA panel<br>snoRNA panel	RNA-based panels generally outperform single targets and can complement CT or nodule triage workflows. Histology-oriented miRNA panels are promising, but RNA integrity, sputum handling, and limited external validation remain major constraints.	Phase 2–3
Metabolomics	10	Diagnosis<br>Screening	FTIR spectroscopy, Raman spectroscopy, GC-MS, FIE-MS	Metabolomic profiles are biologically informative and can	Phase 2

		Monitoring response Surveillance	and ND-EESI-MS for lipid finger-printing, and glucose and glycolytic metabolites	distinguish cancer from controls in exploratory cohorts, but the field remains discovery-stage because of small studies, mixed platforms, and sparse external validation.	
Metagenomics / microbiomics	19	Diagnosis Histologic subtyping Response prediction Monitoring response Prognostication	16S rRNA sequencing for specific taxa (Gemella, Firmicutes, Bacillus, Granulicatella, etc.)	Airway dysbiosis is associated with cancer presence, squamous histology, stage/metastatic phenotype, *EGFR* status, and possibly immunotherapy response. Most models remain exploratory and lack standardized pipelines or robust external validation.	Phase 2
Integromics / multi-omics	9	Screening; diagnosis; nodule triage; risk stratification; early-stage detection	Combined sputum miRNAs plus methylated DNA; sputum-plus-plasma panels; sputum combined with LDCT and clinical predictors; ITALUNG biomarker panel	This domain shows some of the strongest apparent diagnostic performance, especially for early detection and pulmonary nodule triage. Integrated models consistently outperform single-domain approaches, but feasibility, cost, workflow complexity, and prospective validation remain major barriers.	Phase 3 (selected integrated models approaching Phase 4 i.e. prospective clinical validation)
Sputum processing / laboratory techniques	7	Pre-analytic optimization for diagnosis, molecular profiling, and longitudinal monitoring	Cell enrichment methods; fixation/preservation workflows; mucolysis/fractionation protocols; cfDNA/cfRNA preservation; exosome isolation	Processing choices materially affect specimen adequacy, inflammatory contamination, nucleic-acid integrity, and downstream assay performance, making standardization of sputum handling central to translation of the field.	Enabling methods; not directly phase-classified

* Translational maturity as reflected by the current phase of biomarker development, based on the National Cancer Institute Early Detection Research Network's five-phase framework.

Abbreviations: *ALK*, anaplastic lymphoma kinase; *BRAF*, v-raf murine sarcoma viral oncogene, homolog B; CA-FISH, chromosomal aneusomy-fluorescent in situ hybridization; cfDNA, cell-free deoxyribonucleic acid; cfRNA, cell-free ribonucleic acid; CT, computed tomography; ddMSP, droplet digital methylation specific polymerase chain reaction; ddPCR, droplet digital polymerase chain reaction; diaPASEF, data-independent acquisition, parallel accumulation, serial fragmentation; DNA, deoxyribonucleic acid; *EGFR*, epidermal growth factor receptor; ELISA, enzyme linked immunosorbent assay; *EML4*, echinoderm microtubule-associated protein-like 4; EV, extracellular vesicles; FAL-FISH, fractional allele loss-fluorescent in situ hybridization; FIE-MS, flow infusion electrospray ion-mass spectrometry; FISH, fluorescent in situ hybridization; FTIR, Fourier transform infrared (spectroscopy); GC-MS, gas chromatography-mass spectrometry; *HER2*, human epidermal growth factor receptor 2; *hTERT*, human telomerase reverse transcriptase; IHC, immunohistochemistry; *K-RAS*, Kirsten rat sarcoma viral oncogene homologue; LDCT, low-dose computed tomography; MACS, magnetic activated cell sorting; MALDI-TOF, matrix-assisted laser desorption/ionization time-of-flight; mRNA, messenger ribonucleic acid; miRNA, micro-ribonucleic acid; MSI, microsatellite instability; ND-EESI-MS, neutral desorption-extractive electrospray ionization-mass spectrometry; NGS, next generation sequencing; *NRG*, neuregulin; *NTRK*, neurotrophic tyrosine receptor kinase; $p16^{INK4a}$, inhibitor of cyclin dependent kinase-4 family, 16 kDa protein; *TP53*, tumor protein 53; PD-L1, programmed cell death protein 1; PWS, pulsed wave spectrometry; *RASSF1A*, Ras association domain family 1 isoform A; *RET*, rearranged during transfection proto-oncogene; RNA, ribonucleic acid; rRNA, ribosomal ribonucleic acid; SELDI-TOF, surface-enhanced laser desorption/ionization time-of-flight; snoRNA, small nucleolar ribonucleic acid; *SOX*, SRY-box transcription factor; *TAC1*, tachykinin precursor 1; TRAP, telomerase repeat amplification protocol.
